# Effects of High-Fructose Diet *vs*. Teklad Diet in the MNU-Induced Rat Mammary Cancer Model: Altered Tumorigenesis, Metabolomics and Tumor RNA Expression

**Published:** 2021-01-11

**Authors:** Amit Kumar, Ronald A. Lubet, Jennifer T. Fox, William G. Nelson, Harold Seifried, Clinton J. Grubbs, Mark Steven Miller

**Affiliations:** 1 Nutritional Science Research Group, Division of Cancer Prevention, National Cancer Institute, Rockville, MD; 2 Chemopreventive Agent Development Research Group, Division of Cancer Prevention, Rockville, MD; 3 Department of Oncology, Sidney Kimmel Comprehensive Cancer Center at Johns Hopkins University, Baltimore, MD; 4 Department of Surgery, University of Alabama at Birmingham, Birmingham, AL

**Keywords:** Mammary cancer, High-fructose diet, Prevention

## Abstract

Epidemiology, clinical and experimental animal studies suggest high fructose diets are detrimental to metabolic status and may contribute to tumor development. This due to increased obesity and metabolic syndrome, known risk factors for many types of cancer. We compared tumor development in N-methyl-N-nitrosourea (MNU)-treated rats fed either a high (60%)-fructose diet (HFD) or a standard diet (SD). Female Sprague-Dawley rats at 43 days of age (DOA) were fed a SD or HFD followed by administration of MNU at 50 DOA. Rats were palpated weekly and sacrificed at 190 DOA. MNU-treated rats on HFD exhibited decreased tumor latency and roughly a two-fold increase in tumor multiplicity. RNA-Seq on frozen tumors (SD *vs.* HFD rats) showed altered expression of approximately 10% of genes (P < 0.05). When examined by Ingenuity Pathway Analysis, multiple highly significant pathways were identified including A) mechanisms of cancer, B) Wnt pathway, C) immune response (*e.g.*, “Th1 and Th2 activation” and “antigen presentation”) and D) LXR/RXR nuclear receptor. These generalized pathways were indirectly confirmed by alterations of various interrelated disease pathways (epithelial cancers, T cell numbers and apoptosis). In a second study, serum was collected from rats on the HFD or SD pre-MNU and at the time of sacrifice. Metabolomics revealed that the HFD yielded: A) increased levels of fructose, B) increases of various monoglycerols, C) reduced levels of various diacylglycerols and oxygenated inflammatory lipids (9 and 13 HODE and 12,13 DHOME) and D) increased levels of secondary bile acids (hyodeoxycholate and 6-oxolithocholate), which may reflect microbiome changes. These metabolomic changes, which are distinct from those on a high-fat diet, may prove relevant when examining individuals who consume higher levels of fructose.

## Introduction

Studies suggest high fructose diets are detrimental to metabolic status and tumor development [1]. This is due to increased obesity and metabolic syndrome, known risk factors for many types of cancer. The breast cancer model in which mammary tumors are induced in 50-day-old Sprague-Dawley rats by treatment with the carcinogens 7,12-dimethylbenz(a) anthracene (DMBA) or N-methyl-N-nitrosourea (MNU) has been used for many decades [2, 3]. The model has routinely employed a standard natural ingredient Teklad diet (SD), which has relatively low levels of fat (4% fat by weight, 8% fat by calories) and contains corn, soybean meal, wheat, oats, barley, alfalfa meal and dry skim milk as the carbohydrate source and soy as the primary protein source. MNU-treated rats fed a SD develop estrogen receptor-positive (ER^+^) cancers that are similar by gene expression array analyses to highly differentiated ER^+^ human breast cancer [4]. These tumors respond both in a prevention setting and in a therapeutic setting to treatments that are effective in modulating human ER^+^ tumors, including selective estrogen receptor modulators (SERMs; e.g., tamoxifen, toremifene and arzoxifenene), aromatase inhibitors *(*e.g., vorozole and letrozole) and ovariectomy [5, 6]. However, the question has always arisen whether one might obtain different results if one employed other diets. We recently published data comparing tumor results for rats fed a SD to those fed a high fat “western diet”. The Western diet, which more closely resembles the human diet and routinely consumed in the United States [7], contains high fat, high sucrose and low levels of calcium, whereas the SD is low in fat, high in calcium and has a high soy content. Rats fed a Western diet exhibited reduced tumor latency, increased tumor multiplicity and demonstrated a similar response to a variety of preventive agents. However, these rats also displayed a striking increase in body weight.

One dietary component that may contribute to a higher risk of breast cancer is the relative intake of sugars, specifically fructose, because it results in increased obesity, changes in glycemic index, and the associated sequelae [8]. Several studies in the early 2000s pointed to increased breast cancer risk with increased glycemic index and glycemic load [9–12]. The focus on sugars, obesity and disease, primarily non-alcoholic fatty liver disease, has been active for decades as well. This topic has been examined by the World Health Organization, which linked increased “sweet foods” to increased mammary density; high fructose corn syrup was the suspected causal factor [12]. Recently, epidemiologic studies further investigated the association between glycemic index and breast cancer susceptibility in the EPIC study [13]. Furthermore, Dong et al. reported that dietary glycemic index was linked to breast cancer in a large meta-analysis of 10 prospective studies [14]. It should be noted that breast cancer risk in many publications is linked to relative glycemic index and overall sugar intake and not specifically to the intake of high fructose. However, reviews have discussed the biochemical mechanistic effects of increased fructose in foods compared to sucrose and glucose and highlight why the emphasis should focus on fructose and other related diseases [15, 16]. Strober and Brady [8] have explored the links between increased dietary fructose and the incidence of triple-negative breast cancer from a mechanistic standpoint. More specifically, there have been two recent animal model papers showing that employing fructose as a primary sugar source enhances mammary cancer and that a sucrose- or fructose-enriched diet promotes tumorigenesis in the mammary gland through elevation of 12 lipoxygenase [17]. A second study showed that high fructose corn syrup in water, which resulted in a significant increase in body weight, enhances intestinal tumor growth in preclinical mouse models [18]. Based on the heightened interest on obesity and dietary sugars, primarily the shift to high fructose sweeteners as a sweeter and cheaper alternative to sucrose [19], we wished to expand our observations regarding the high-fat Western diet to a diet employing fructose as the primary sugar source. Thus, in the current studies, we employed a defined isocaloric diet containing high levels (60%) of fructose (HFD) as a primary energy source.

Investigators have commonly analyzed RNA expression in tumors to gain mechanistic insights regarding specific agents and to compare animal tumors employed in therapeutic or chemoprevention studies to the human tumors they are intended to model. For example, Chan et al. [4] showed that MNU-induced ER^+^ rat mammary tumors are similar to highly differentiated estrogen receptor- positive/progesterone receptor-positive (ER^+^/PR^+^) tumors in humans. Lu et al. demonstrated that N-butyl-N-(4-hydroxybutyl)nitrosamine (OH-BBN)-induced rat bladder tumors [20] appear similar to basal bladder cancers and lung squamous cell cancers induced by N-nitrosotris-(2-chloroethyl)urea (NTCU) in mice and have significant overlap with human squamous cell carcinoma (SCC) [21]. We have also shown that the determination of RNA expression following the exposure of breast lesions to specific agents such as tipifarnib (a farnesyltransferase inhibitor) and vorozole (an aromatase inhibitor) allows one to determine both pathways and specific gene sets that are modulated by these agents [22, 23]. Breast cancers developed more rapidly and had a higher multiplicity in animals on the HFD in the present studies. Therefore, we examined whether there were differences in RNA expression between the tumors that developed in animals on the two different diets. We initially investigated the effects on differential expression of individual genes with or without a Benjamini-Hochberg (BH) correction for multiple comparisons using transcriptome analysis pipeline in Partek Flow. We then examined the effects on various pathways and disease/function by employing a “core analysis module” in the ingenuity pathway analysis (IPA) application (Qiagen). The results revealed alterations in pathways related to Molecular Mechanisms of Cancer, the Wnt Pathway, Th1/Th2 immune pathways, and the LXR/RXR nuclear receptor pathway.

Finally, we performed metabolomics in animals on the two diets. The reason for doing so was two-fold. First, we wished to determine whether there are metabolite changes associated with exposure to a HFD *vs.* SD that might prove applicable as pharmacodynamic biomarkers of humans consuming high levels of fructose. This is particularly important since it has been proposed that a HFD induces a variety of metabolic changes that may contribute to the development of diabetes or heart disease. Second, our prior studies [7, 24] had shown that the use of the high-fat diet, which also enhances tumor development in the MNU-induced model, altered a variety of metabolic pathways, and the question arose whether exposure to a HFD might elicit some of the same metabolic changes.

## Materials and Methods

### Animal treatments

The MNU-induced model of ER^+^ rat mammary cancer [6, 7], which has routinely been employed in screening chemo preventive agents, was used to evaluate the effects of altered diet. We utilized both the standard protocol, which calls for an injection of MNU into Sprague-Dawley rats at 50–57 days of age (DOA, “early exposure”) and a modified protocol, whereby MNU was injected at 100 DOA (“late exposure”). The rationale for the late exposure was that it allowed for longer exposure to the modified diet (standard exposure HFD DOA 50–200; late exposure HFD DOA 43–300). In these experiments, the MNU dose was reduced from 75 mg/kg to 50 mg/kg due to the high toxicity we observed in preliminary studies when the rats were injected at 100 days. All animal studies were conducted under a protocol approved by the University of Alabama at Birmingham Institutional Animal Care and Use Committee.

For the early exposure protocol, twenty-eight-day-old female Sprague-Dawley rats were obtained from Harlan Sprague-Dawley, Inc. and placed on a standard 4% Teklad diet (SD; catalog# 7001; 3.0 kcal/gm, Envigo, Madison, WI; Table S1) upon arrival. At 50 DOA, half of the rats were switched to an isocaloric Teklad 60% high-fructose diet (HFD; catalog# TD.89247; 3.6 kcal/gm; Table S1). At 57 DOA, all of the rats were injected with a single dose of MNU as described above. Rats were palpated 2x/week for mammary tumors. The rats were sacrificed at approximately 200 DOA. The euthanasia of all study groups was carried out by CO_2_ asphyxiation. The rats were sacrificed at approximately 200 DOA. The euthanasia of all study groups was carried out by CO_2_ asphyxiation. At the termination of the studies, all of the mammary tumors were weighed. Ten tumors from each group were fixed in 10% formalin for histology, stained with Hematoxylin and Eosin (H&E), and histologically classified by a board-certified pathologist (MMJ). All tumors were diagnosed as minimally invasive adenocarcinomas. Ten additional tumors were snap-frozen for subsequent RNA studies. For the late exposure protocol, rats were placed on SD or HFD at 43 DOA. At l00 DOA, they were injected *via* the jugular vein with 50 mg/kg of MNU dissolved in saline. Rats were palpated weekly for mammary tumors. The rats were sacrificed at approximately 300 DOA. At day 98 (2 days prior to MNU treatment) and at the end of the study, serum was collected from rats on the SD or HF diet to be employed in the metabolomics studies.

### Statistical methods for prevention studies

For comparisons between groups, the Mann-Whitney Rank test was used in the analyses of final mammary tumor weights and tumor multiplicity as previously described [7]. Log-rank analysis was employed to determine tumor latency [6].

### RNA library preparation and sequencing

Total RNA from 50 µg mammary tissue was extracted using the mRNeasy mini kit from Qiagen (Cat# 217004, Redwood City, CA, USA) according to the manufacturer’s recommendation. The RNA quality was analyzed using the agilent RNA 6000 nano kit (Cat# 5067–1511) and agilent 2100 bioanalyzer system (2100 bioanalyzer instrument, cat# G2939BA, 2100 expert software, Santa Clara, CA) [25]. Only samples with high RNA quality (RNA integrity number of 9 or higher) were processed further. 500 ng of total RNA was used for the library preparation using the Illumina TrueSeq stranded mRNA library prep kit and standard protocol (Cat # 149 RS122–2101, Illumina Inc. San Diego, CA). The fragment size of the library was analyzed using the agilent DNA 1000 Kit, and the concentration of DNA in the library was measured using the Qubit 1xdsDNA HS assay kit (Cat# Q33230) and qubit fluorometer (Cat# Q33226, ThermoFisher Scientific, Waltham, MA) using the protocol suggested by the manufacturer. An equal amount of the library from samples was pooled (4 µl) and sequenced on an Illumina NextSeq 550 using the NextSeq 500/550 High Output v2 kit (75 cycles, Cat# FC-404-2005, Illumina Inc. San Diego, CA). The steps and program for sequencing and data collection were followed. Sequence files were generated in the FASTQ format at Illumina BaseSpace.

### Differential expression and pathway analysis

FASTQ files were imported and analyzed using the following pipeline in the PartekFlow genomic analysis software (Partek, St. Louis, MO, USA). The 75 bp sequence was trimmed to 73 bp based on a Phred score of 30 or higher. The reads (~ 35–50 million/sample) were aligned to the Rattus norvegecus reference genome (rn6) using STAR aligner (2.5.3a). A prebuild R. norvegecus index (rn6-RefSeq Transcript 82-2017-08-02) was used as the reference for transcript build and count. These counts were normalized using the counts per million method. Differential gene expression (p-value and fold-change) was calculated using the LIMA (Lognormal with shrinkage) model.

The threshold for identifying differences between the control and treatment group is reported in the Results section. Datasets containing gene identifiers and corresponding expression values (fold change) generated from the control and treatment conditions were uploaded into IPA software (Ingenuity Pathway Analysis (IPA®, QIAGEN Redwood City, http://www.qiagen.com/ingenuity, Version 60467501, dated 1/19/2020 [26]). The information in the ingenuity knowledge base (Genes Only) as a reference set that considers both direct and indirect relationships and all species (human, rat, mouse) was applied towards analysis. The gene list was then subjected to disease and function (GO) analysis to evaluate over-represented signaling pathways, biological processes and functions using expression “core analysis” workflow. IPA Core analysis module was also run to cluster the genes based on biological functions and IPA compare function was used to identify the genes that were common among different pathways and disease conditions. Furthermore, my pathway and pathway designer tool was applied to create a visual representation of genes that were shared or unique among different signaling pathways. The “grow” tool in IPA was applied to overlay the disease and signaling pathways.

### Metabolomic analysis of the effects of diet on mammary tumor metabolism

The serum was stored at ‒80 ℃ until processed. The processing and metabolomics analytical procedures were carried out as described recently [6, 22, 25] and are based on analytic methods developed by Metabolon [26, 27]. Specific dietary control animals not administered MNU (5 rats per group) were run in parallel to the 100-day-old study. The groups were: control SD 98 DOA, HFD 98 DOA, control SD 300 DOA, and HFD 300 DOA. At the indicated time points, animals were sacrificed, and sera was collected and stored in liquid nitrogen.

### Mass spectrometry analysis

Non-targeted MS analysis was performed at Metabolon, Inc. Extracts were subjected to either GC184 MS or UPLC-MS/MS [27, 28]. The chromatography was standardized and once the method was validated, no further changes were made. Each vacuum-dried sample was dissolved in injection solvent containing eight or more injection standards at fixed concentrations, depending on the platform. Samples to be analysed by GC-MS were dried under vacuum desiccation for a minimum of 18 h prior to being derivatized under dried nitrogen using bistrimethyl-silyltrifluoroacetamide. Derivatized samples were separated on a 5% diphenyl/95% dimethyl polysiloxane fused silica column (20 m × 0.18 mm ID; 0.18 um film thickness). Samples were analyzed on a Thermo-Finnigan Trace DSQ fast-scanning single-quadrupole MS using electron impact ionization (EI) and operated at unit mass resolving power. The scan range was from 50–750 m/z. Metabolites were identified by automated comparison of the ion features in the experimental samples to a reference library of chemical standard entries that included retention time, molecular weight (m/z), preferred adducts and in-source fragments, as well as associated MS spectra. They were curated by visual inspection for quality control using software developed at Metabolon [27].

## Results

### Effects of diet on mammary tumor development

**Figure 1:**
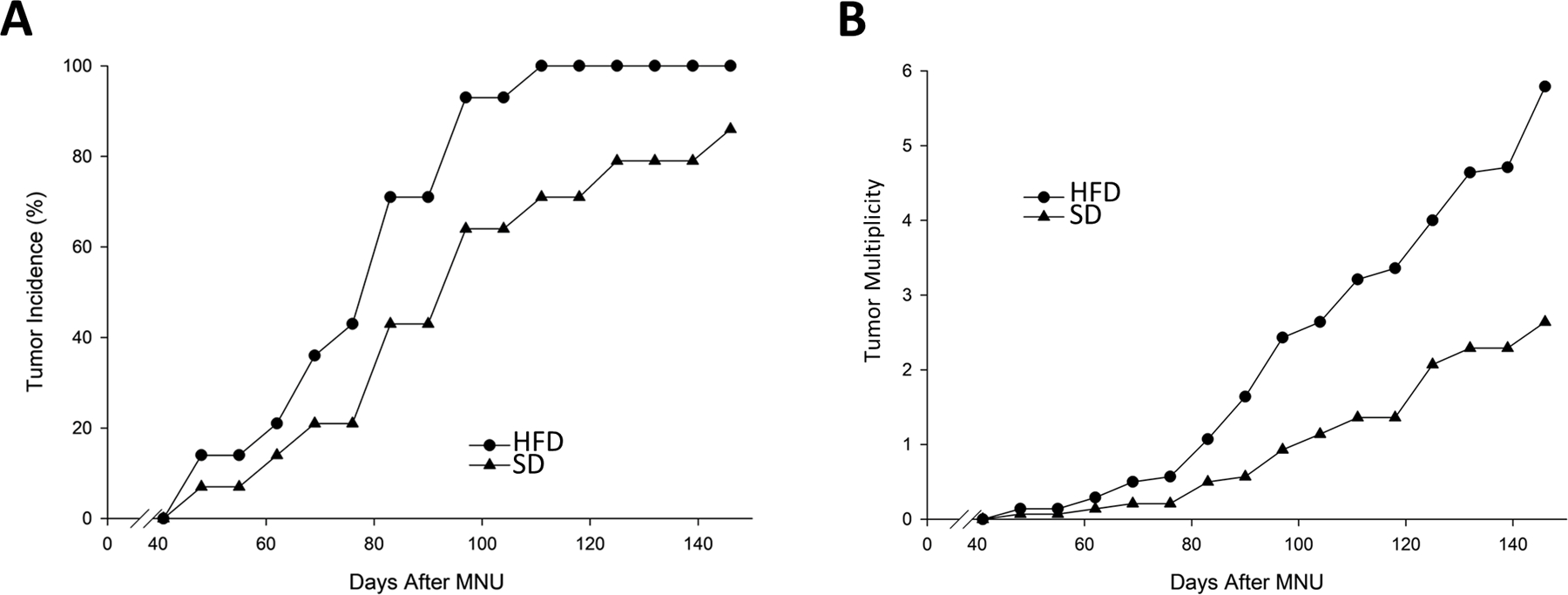
Effect of HFD on tumor incidence and multiplicity in the early exposure protocol. Rats were placed on the HFD at 50 DOA and treated with MNU (50 mg/kg BW) at 57 DOA. **(A)**- Tumor incidence and **(B)**- multiplicity were determined twice per week by palpation.

To examine the effects of diet on tumor development, metabolism, and tumor RNA expression, we evaluated the effects of SD and HFD (Table S1) in the MNU-induced rat model of mammary cancer. Carcinogen treatment with MNU was performed at two different time points. In the “early exposure” protocol, rats were injected intravenously (IV) with a single dose of 50 mg/kg MNU *via* the jugular vein at 57 DOA and followed until 200 DOA (approximately 150 days after initiating MNU treatment). In the “late exposure” protocol, rats were placed on a SD or HFD at 50 DOA and treated with 50 mg/kg of MNU at 100 DOA. Rats were palpated weekly and followed until approximately 300 DOA (approximately 250 days post-MNU). In the early exposure study, the HFD group did not exhibit any significant changes in body weights relative to the SD group. This was expected since the two diets were isocaloric. The difference in tumor incidence (Figure 1A) between the two dietary groups, although not striking, did achieve statistical significance when employing a log-rank analysis. The difference in tumor multiplicity (Figure 1B) was more pronounced yielding roughly a two-fold increase in tumor multiplicity (P < 0.05 Mann Whitney nonparametric rank test). We also observed an increase in final tumor weights of approximately 35% when comparing tumors from rats on a HFD diet to those on a SD, although it did not achieve statistical significance (data not shown).

In the late exposure study, in which MNU was administered at 100 DOA and the animals were sacrificed 200 days post-MNU, the HFD group similarly did not exhibit any significant changes in body weight relative to the SD group despite having been on the HFD for almost 8.5 months. Although there appears to be a marginal difference in tumor incidence (Figure S1A) and more clearly, in final tumor multiplicity (Figure S1B) when comparing the HFD and SD groups, neither of these effects were statistically significant. The rats on the HFD exhibited an approximately 2-fold increase in final tumor weights relative to those on the SD (2.94 g in the HFD group *vs.* 1.44 g in the SD group), but this value did not reach statistical significance (0.1 > P > 0.05).

### Effects of diet on serum metabolites

**Figure 2:**
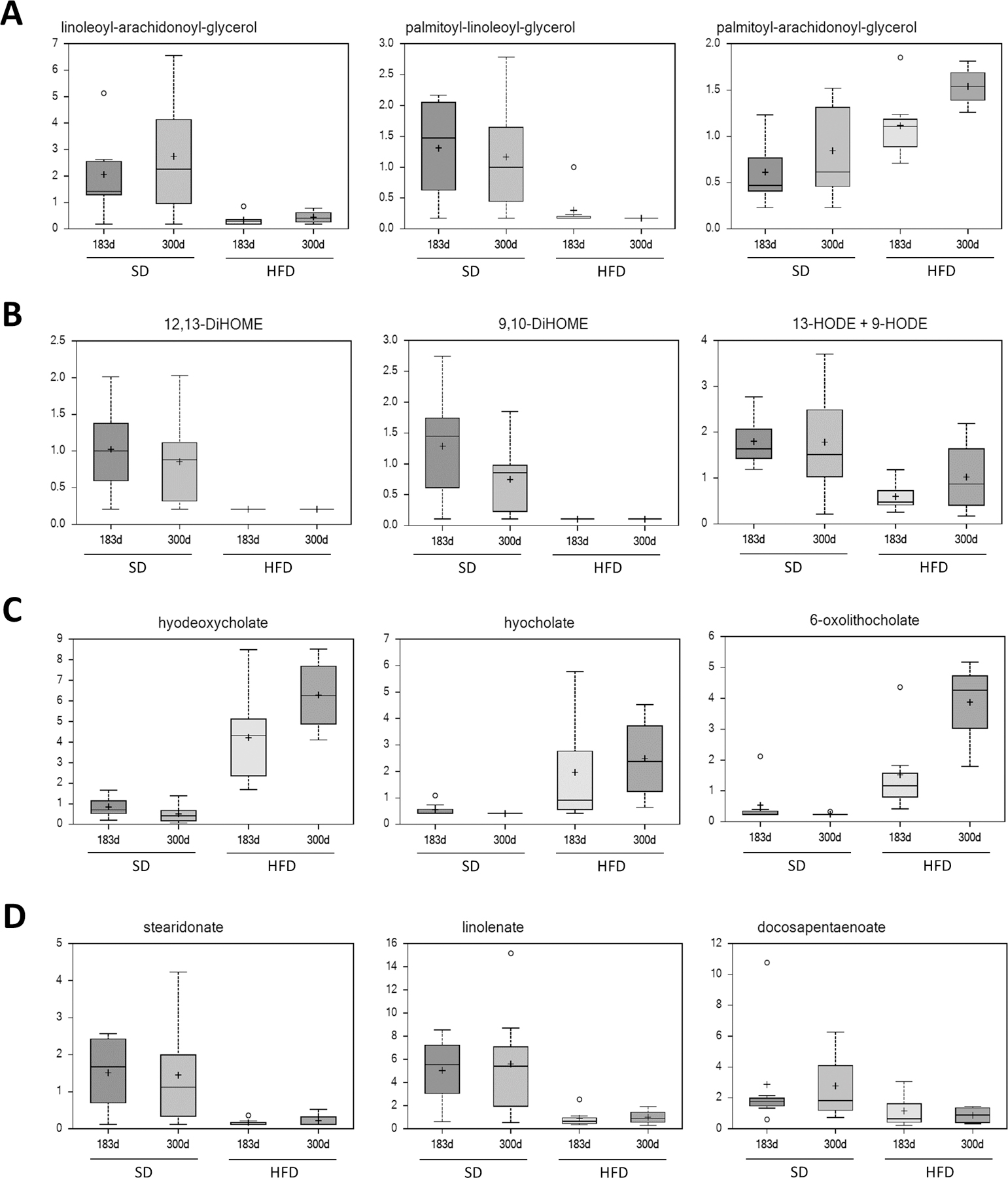
Relative levels of metabolites in sera from rats on an HFD or SD at 183 DOA and 300 DOA. Rats were placed on the respective diets at 43 days of age. **(A)**- Serum levels of diacylglycerols. **(B)**- Serum levels of mono- and dihydroxy-fatty acids, which are potentially immunomodulatory. **(C)**- Serum levels of secondary bile acids. **(D)**- Serum levels of polyunsaturated fatty acids.

Five rats each from the late exposure SD and HFD groups were euthanized at 98 DOA and sera from 5 rats per group were also obtained at the end of the study (300 DOA). Sera from both groups of rats were collected and quick-frozen for metabolomics analyses. Serum metabolite levels were markedly different in rats fed a HFD compared to those fed a SD (Figure 2A–D, Table S2). The only changes presented are those that were consistent at both time points (98 and 300 DOA). As expected, based on the fact that the SD employs a soy mixture as a protein source, a variety of metabolites specifically associated with soy (e.g., genistein and daidzein) were high in the SD group (Table S2). Not unexpectedly, higher levels of fructose (2 to 2.5-fold) were observed in rats on the HFD (data not shown). In addition, there were a variety of metabolic pathways altered in animals fed an HFD. Altered expression of various diacylglycerols (e.g., decreases in linoleoyl-arachidonyl-glycerol and palmitoyl-linoleoylglycerol and increases in palmitoyl arachidonly-glycerol) were observed in serum (Figure 2A and Table S2) and livers from the same rats (data not shown). A surprising decrease in certain mono or dihydroxy fatty acids that are mediators of inflammation, including 9 and 13 HODE, 12,13 DHOME, and 5,6 DiHete, was also observed (Figure 2B). One additional class of metabolites that is greatly increased in the HFD rats and which may be related to gut microbes, is that containing the various primary and secondary bile acids (deoxycholate, hydrodeoxycholate, and 6-oxolithocholate) (Figure 2C and Table S2). There were reductions in the levels of various sphingolipids, polyunsaturated fatty acids (n3 and n6) (Figure 2D) and several metabolites associated with benzoate metabolism (Table S2). Interestingly, the levels of various tocopherol metabolites were also significantly altered.

### Effects of diet on RNA expression in tumors (early protocol)

**Figure 3:**
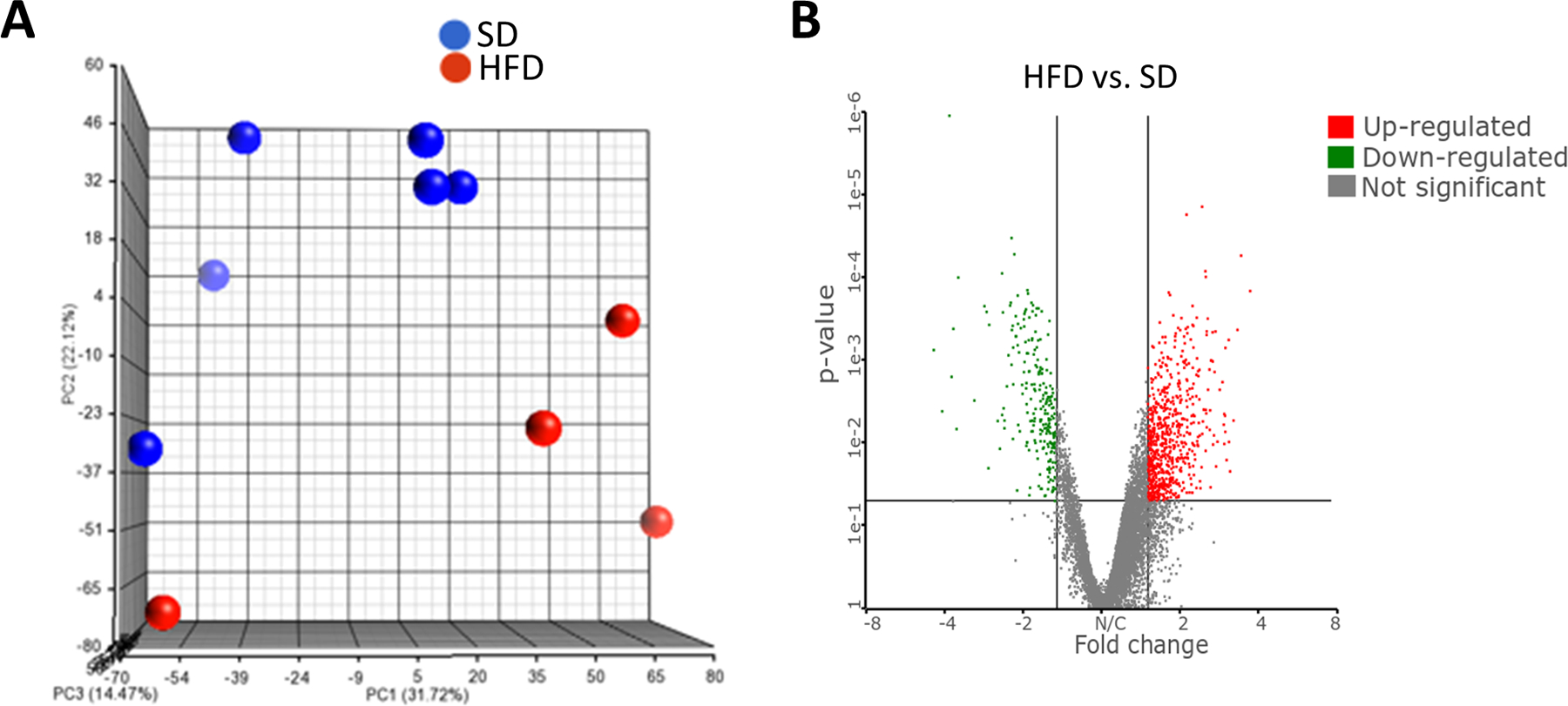
A global overview of gene expression levels among all of the controls (n = 6) and high fructose-treated animals (n = 4). **(A)**- Principal component analysis (PCA) of gene expression of animals were evaluated. Th x^‒^, y^‒^ and z-axes of the three-dimensional space define the three components PC1, PC2 and PC3 respectively. The distance between the points reflects the variance in gene expression among them. PC1, PC2 and PC3 accounted for 31.7%, 22.1%, and 14.4%, respectively, of the contribution to the variance. **(B)**- Volcano plots for differentially expressed genes between the control group and animals fed an HFD are presented. Each gene is marked as a dot (p < 0.007, FDR < 0.2, up-or down-regulated > 1.5-fold). Those genes that are up-regulated are shown in red, and the down-regulated genes are represented by green dots. The grey horizontal lines denote the p-value threshold (p < 0.007) and the vertical lines the fold-change cutoff (> 1.5).

**Figure 4:**
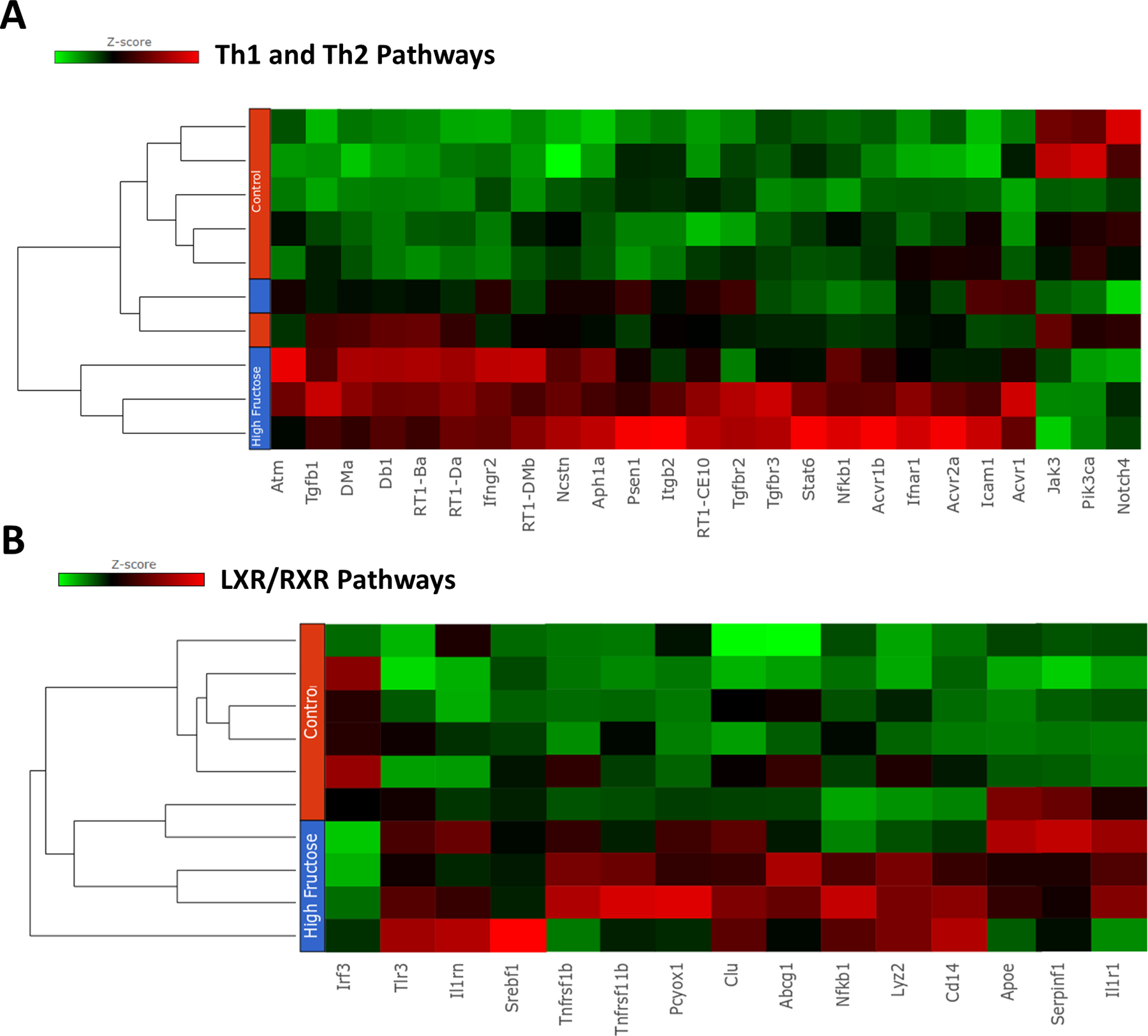
Heat map representing expression levels of differentially expressed genes (p < 0.05, up-or down-regulated > 1.5-fold) in animals on a SD (controls, n = 6) or an HFD (n = 4). Differentially expressed genes were analyzed by IPA. **(A)**- Heat map of genes enriched in the Th1 and Th2 pathways. **(B)**- Genes enriched in the RXR/LXR pathway. Red blocks: genes up regulated, green blocks: genes down regulated.

To investigate the genes and pathways affected in the studies outlined above, we employed RNA sequencing on mammary tumor tissue isolated from rats on the SD (N = 6) and from rats on the HFD (N = 4) at the end of the study. When we examined the RNAs of the two groups using a principal component analysis, we found that they were distinctly different (Figure 3A). The six samples from the SD group clustered together, while three of the four samples from the HFD group clustered together. However, all four were clearly separated from those in the SD group. Of the 8085 sequences mapped, 880 genes showed altered expression (p-value < 0.05, fold-change (FC) > 1.5), 662 showed increased expression and 228 showed decreased expression (Table S3). A visual representation of the genes that were significantly upregulated or downregulated is presented in a volcano plot (Figure 3B). When the data was corrected for multiple testing using Benjamini-Hochberg (BH) method and transcripts were analyzed using more stringent criteria (BH false discovery rate (FDR) < 0.07 and 1.5-fold-change in gene expression), 87 genes showed altered expression (42 genes increased and 45 genes decreased, Table S4). The genes that were found to be altered when using the more stringent criteria are marked bold in the list of genes associated with major pathways (Table 1 and S5). The 880 genes obtained using the less-stringent cut-off (BH FDR < 0.2,p < 0.05, FC 1.5 -up or -down) were analyzed by IPA (setting: direct and indirect analysis; confidence all species include human, mouse and rat and all cell lines and tissue) to determine the molecular pathways and associated diseases/functions affected in the HFD-treated animals. The most significantly affected pathways include those related to Th1 and Th2 activation, molecular mechanisms of cancer, antigen presentation pathway, Wnt β-catenin and the LXR/RXR activation pathways. All pathways were significant at levels p < 5 × 10^‒6^ and remain highly significant after corrections were made for multiple comparisons. In table 1, we present the data associated with these five pathways, including statistical analyses (with or without corrections for multiple analyses), the number of elements in each pathway, the number of elements identified by our RNA sequencing, and the numbers of genes that were identified as being altered when the less stringent statistics were applied. We also present the individual genes altered in each of the pathways, including the individual genes that were highly significant with a BH correction (marked bold). In general, 15–35% of the identified genes in a given pathway are found to have altered expression when the less-stringent RNA criteria are employed. These altered pathways include genes associated with mechanisms of cancer and the Wnt pathway, which is clearly associated with cancer in a number of organs. In addition, there were changes in pathways associated with the immune response and genes associated with the LXR/RXR nuclear receptor pathway (which affects both lipid metabolism as well as immune functioning in macrophages and certain cells associated with the innate immune response). The changes in the pathway associated with the mechanism of cancer and the Wnt pathway would seem to agree with the enhanced tumor progression observed in rats on an HFD. It has often been hypothesized that high-fat or high-fructose diets may be associated with a proinflammatory state, which may contribute to the overall development of tumors. This alteration in the immune response partially reflects the alterations in the LXR/RXR nuclear receptor pathway. A few pathways that are critical for tumor metastasis, such as the regulation of epithelial-mesenchymal transition and neuroinflammation signaling pathways, were also significantly represented (BH multiple correction values < 0.05) in the HFD-fed animals. In order to give readers a visual representation of the gene expression changes in individual samples associated with these pathways, we present two heat maps associated with the immunologic (Th1/Th2) pathways (Figure 4A) and with the RXR/LXR pathways (Figure 4B). These two figures show that for these two pathways, the heat maps for tumors from the SD and HFD animals are quite distinct. However, if we only examine the genes that were significant after a correction for multiple testing, only 1/15 genes (CD14) in the LXR/RXR pathway and 0/23 genes for the Th1/Th2 pathway achieved significance. This emphasizes the strength of pathway analysis as contrasted with the examination of individual genes. In general, gene expression in both pathways is increased in tumors from rats on the HFD.

Finally, to perform a secondary confirmation of the results examining the generalized pathways above, we employed analysis directed to more specific disease-related process and function in IPA (Table 2). We present three pairs of disease-related processes: A) immune-related pathways (Leukocyte Migration and Quantitation of T Cells), B) cell survival pathways (Organismal Survival and Apoptosis) and C) cancer pathways (Epithelial Neoplasms and Gastrointestinal Cancers). It is worth noting that the statistical significance of most of these pathways is profound, either corrected or uncorrected for multiple comparisons; and that the Leukocyte Migration pathway and Quantitation of T Lymphocytes are both increased, while the Organismal Survival and Apoptosis pathways are both decreased.

**Figure 5:**
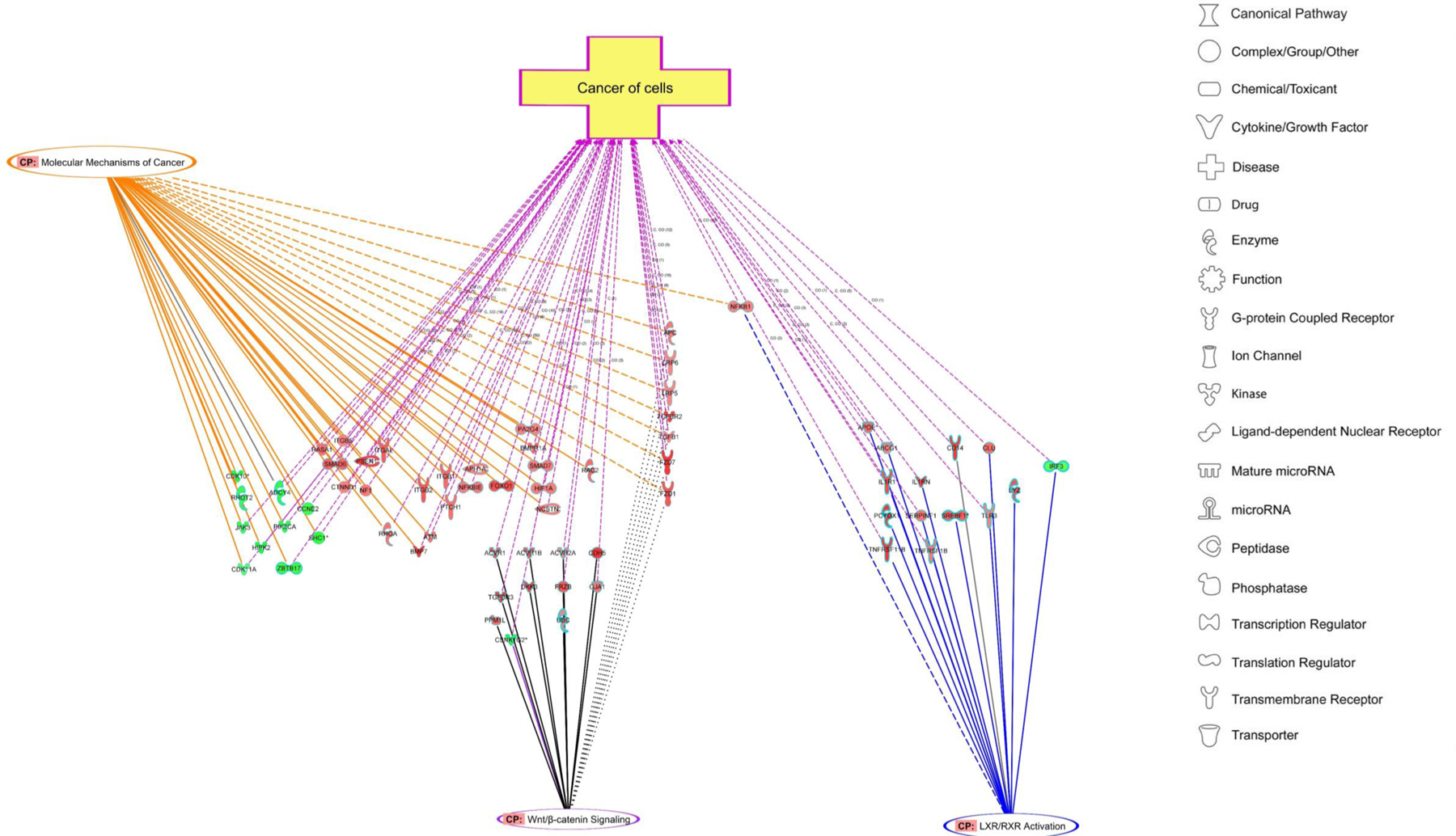

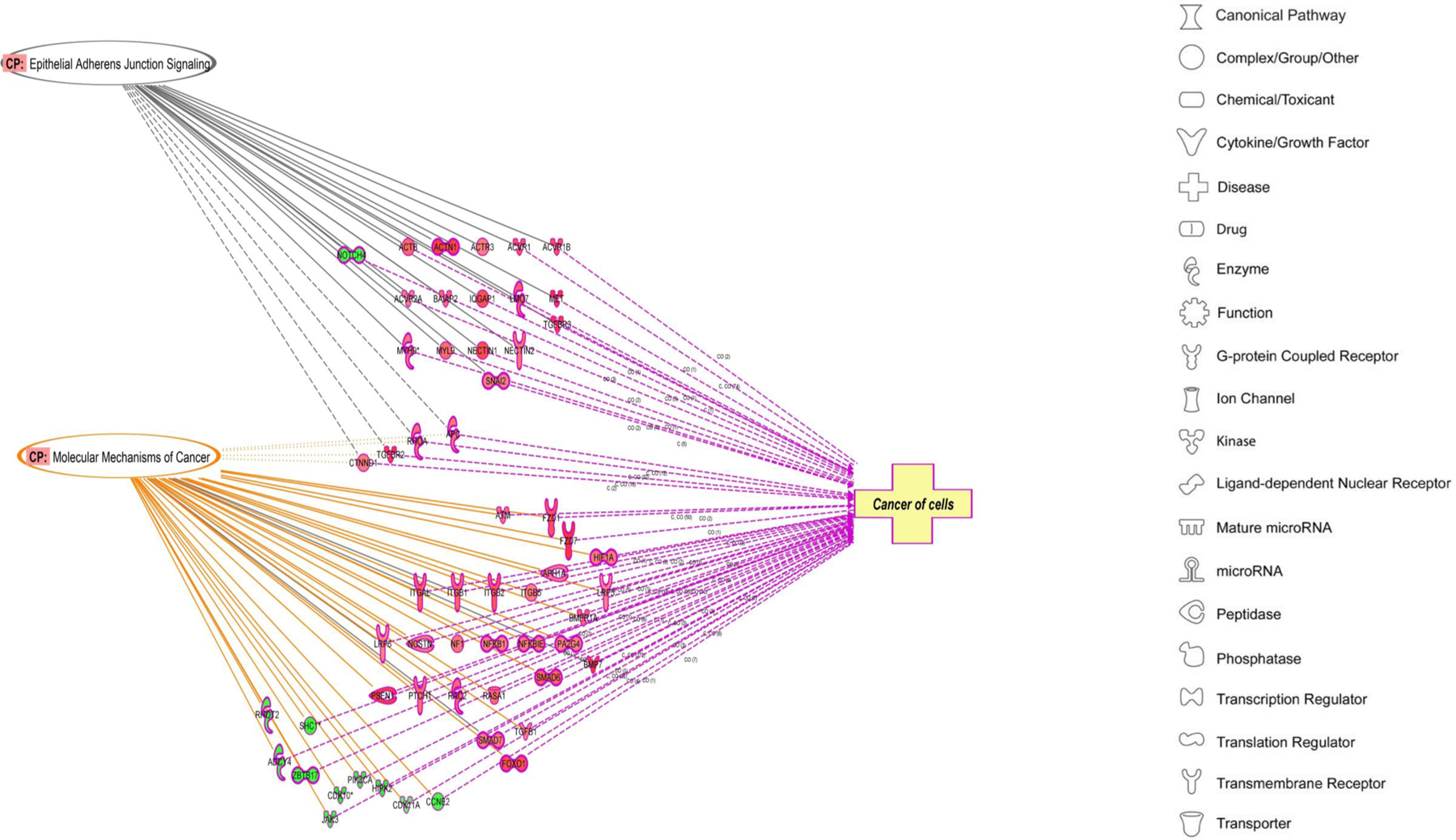
Enrichment of differentially expressed genes in canonical pathways and disease/function features analyzed in IPA. The green fill is down-regulated and red fill is up-regulated genes (p < 0.05, FC = 1.5 -up or -down) between control group and animals fed with HFD. The shape represents the type of molecules (kinase, receptor etc.) as represented in the figure. There is little overlap between LXR/RXR and Molecular Mechanism of Cancer or Wnt/ beta-catenin pathway but most contribute to the disease/function cancer of cells. **(A)**- There is a limited gene shared between epithelial adherens junctional signaling and molecular mechanism of cancer but both pathways contribute to the function Cancer of Cells **(B)**- CP: Canonical Pathway, solid line represents the genes specific to pathway, dotted line represents the genes common among various canonical pathway and disease/function. CO: Correlation between the molecules and/or annotation and number indicate the literature finding curated in IPA knowledge base.

In figure 5, we present graphically the relationship between the genes mediated in three of the Pathways identified in table 1 (molecular mechanisms of cancer, Wnt-β catenin, LXR/RXR activation). The genes altered in the molecular mechanism of cancer and the Wnt pathways have substantial overlap. In contrast, the LXR/RXR Pathway has no overlap with the Wnt pathway and modulation of only a single gene compared with molecular mechanisms of cancer (Figure 5A). When canonical pathways (CP) were overlaid on the disease/function in IPA, there was limited overlap between molecular mechanism of cancer or Wnt-β catenin and LXR/RXR pathways (Figure 5A); however, the genes in all three CP contributed to the disease process of “cancer of cells” highlighting the contribution of different pathways towards a common disease/function. Similarly, both CPs epithelial-adherence junction and molecular mechanism contributed towards cancer of cells with little overlap among the genes between the pathways (Figure 5B).

## Discussion

While the risk for breast cancer has been associated with obesity [9–11, 29] and higher intakes of sweet foods (a.k.a. the Western Diet) with the increased consumption of high fructose corn syrup [30–34], the extent to which various dietary components increase breast cancer risk is still unclear. The increased consumption of fatty acids and/or sugars, in addition to inducing an obese state, can also alter the metabolic profile of an individual, leading to a pre-diabetic state and potentially altering tumor development. In fact, a number of epidemiologic studies have shown an association between a high glycemic index, routinely associated with high levels of sugar intake and susceptibility to breast cancer [13, 14]. Recent studies by Gunter et al. [35] have suggested that the metabolic health of the individual, rather than obesity itself, maybe a key risk factor for breast cancer and demonstrated that the levels of fasting insulin are associated with an increased risk for breast cancer. Furthermore, the consumption of a high sugar diet (sucrose or fructose) has resulted in an increase in tumor growth in a mouse model of triple-negative (ER-PR-Neu-297) breast cancer [17]. The authors attributed the increased tumor development in part to alterations in the production of eicanosoids in the tumor. Furthermore, in a preclinical mouse model of colon cancer, high levels of fructose administered in water caused significant weight increases, and a rapid increase in colon cancer was observed [18]. Chemopreventive agent testing in the MNU models has routinely been conducted in rats on normal chow diets that maintain optimal animal weight and health but have lower levels of fat and simple sugars than the standard human diet. Thus, the standard chow diets routinely employ complex carbohydrates with simple sugars such as sucrose representing roughly 5% of caloric intake; humans may be more likely to ingest significant amounts of simple sugars such as fructose or sucrose. Our laboratory has been conducting experiments to compare ER^+^ mammary tumor development and the response to Chemopreventive agents between MNU-treated rats on a SD and MNU-treated rats on either a Western diet (high fat, low calcium) or an HFD. One should note that the HFD we employed was isocaloric with the standard diet and that could explain the lack of significant change in weight increase in the present study. This is unlike the other mouse models, triple-negative breast cancer or the mouse colon study, in which high fructose resulted in substantial increases in body weight [8]. We recently reported data comparing the SD to a Western diet in control groups as well as groups treated with five different preventive agents (tamoxifen, the aromatase inhibitor vorozole, the RXR agonist Targretin, Lipitor and metformin) [7, 24]. Those studies showed that the Western diet altered tumor development, decreasing tumor latency and increasing tumor multiplicity and final tumor weights. However, the response to the various effective (tamoxifen, vorozole, or Targretin) or ineffective (Lipitor or metformin) preventive agents was the same on either diet. The increases in tumorigenesis in rats on the Western diet were associated with increased body weight, which may itself affect the results. The present study focuses on the effects of an isocaloric, HFD on breast cancer formation. We hypothesized that rats on an isocaloric diet would not gain excessive amounts of weight but would demonstrate altered metabolism as a result of the high intake of fructose. Thus, any changes in tumor progression observed would be independent of weight gain.

In examining the effects of the HFD in the MNU-induced model of ER^+^ mammary cancer, we employed two different protocols. The first, the “early exposure” protocol in which MNU is administered to adolescent rats at 50 DOA, is the standard protocol [2, 3, 6, 7]. The second, the “late exposure” protocol, employs older perimenopausal rats (100 DOA at the time of MNU exposure) with a monitoring period of approximately 200 days. In the early protocol, the HFD (Figure 1) resulted in a significant (as determined by log-rank analysis) decrease in tumor latency in rats administered MNU. This was accompanied by an increase in tumor multiplicity in rats on the HFD of almost 2-fold (P < 0.05). Although not statistically significant, the average tumor weights were also increased by approximately 35%. The reduction in tumor latency and increase in tumor multiplicity in the early MNU protocol was surprising since rats were on the HFD for only about 8 weeks when tumors started to develop (50 DOA to 106 DOA). Our expectation was that we might observe more striking results when employing the late model since rats were on the HFD diet for approximately 180 days when tumors start to appear (43 DOA HFD initiated, 100 days MNU; 120 days post-MNU (Suppl. Figure 1). Although the tumor latency was decreased and the tumor multiplicity and tumor weight were increased in the HFD group compared to the SD group enrolled in the late protocol, none of the differences reached statistical significance. This was most likely due to the presence of fewer proliferating mammary epithelial cells in the older rats (Grubbs, unpublished data) resulting in tumor incidences and multiplicities (1.9 in the HFD group and 1.2 in the SD group) that were significantly lower than in the early protocol (tumor multiplicities of 6.0 and 2.7 in the HFD and SD groups, respectively) despite the fact that they were followed for more time (140 days post-MNU early study *vs.* 200 days post-MNU delayed study). This means that the use of the late protocol requires more animals and a longer observation period, hurdles that have routinely been encountered when employing this model.

We examined RNA expression in mammary tumors derived from SD and HFD rats in the early exposure protocol. The rationale for examining RNA expression in samples generated under this protocol was that the tumors in the HFD rats had a shorter latency and higher multiplicity than tumors derived from rats on the SD. A principal component analysis revealed that RNA expression in the SD and HFD group was distinctly different. On an individual gene level, approximately 10% of genes were identified as being differentially expressed (880 of 8085 sequences (p-value < 0.05, FC > 1.5 -up or -down). However, this number was reduced to 1% (87/8085) when more stringent criteria (BH FDR < 0.07, p < 0.007 and 1.5 FC) were employed. In addition, we present a volcano plot (Figure 3B) which gives a visual representation of the genes that are statistically different in expression between tumors from the animals on the two diets. The 880 genes identified using the less stringent criteria were analyzed with IPA using the core analysis module to determine the molecular pathways affected in HFD-treated animals (Table 1). The various pathway changes we observed include Molecular Mechanisms of Cancer, which agrees with the enhanced tumor progression observed in rats on an HFD. Furthermore, the specific genes and sub pathways associated with the molecular cancer pathways recommend avenues to examine the ability of a HFD to enhance tumor formation. A number of the genes identified in the Molecular Cancer Pathways were also identified in the Wnt/Beta-catenin signaling pathway. Interestingly, when we looked at the subset of disease related pathways employing IPA analysis, there were a variety of pathways associated with abdominal cancer (Table 2). This is not surprising since alterations, typically mutations, in the Wnt/Beta-catenin pathway are indeed the most common alteration observed in sporadic colon and intestinal cancer. The Wnt pathway is also significant in breast cancer where it plays a role in cell growth, differentiation, and stem cell self-renewal [36]. Multiple genes involved in the Wnt signaling pathways were altered in expression, including APC, TGFβ1, TGFβ1 Receptor, and multiple members of the G-protein-coupled receptor “frizzled” family. The Wnt pathway is also involved in breast cancer. Thus, high expression of Wnt 1 under the control of an MMTV promoter induces breast cancer in mice [37] and yields two distinct histopathologic breast cancer subclasses based on RNA profiling [38]. In addition, our examination of the disease pathways elicited strong signals from cell survival and apoptotic pathways, which reasonably can contribute to cancer development as well (Table 2).

It has been hypothesized that high-fat or high-fructose diets are associated with an altered immune response, which may contribute to the overall development of tumors. In fact, the preponderance of the genes in the Th1/Th2 pathway are overexpressed in tumors from rats on the HFD. However, the effects on the immune pathways observed in the tumors is complex. While activation of Th1 cells has often been associated with a better prognosis in certain cancers, activation of suppressor-related immune cells, either regulatory T cells (Th2 pathway) or suppressing monocytes, has often been seen as a poor prognostic factor. The activation of the Th2 pathway and the possibility of enhancing regulatory T cells appear to be preferentially altered in the T cell activation (data not shown). When we performed analysis of the altered genes in IPA employing the disease and function feature, we found that a variety of immune related pathways (e.g., leukocyte migration and number of T cells) were strikingly (P < 10^‒14^) increased in tumors from rats fed the HFD (Table 2). In addition, and in agreement with our observed alterations in the immune response, the LXR/RXR pathway, which is activated (see below), can, *via* alterations in lipid metabolism, alter proinflammatory gene expression in macrophages. In addition to altering genes associated with cell-mediated immune response, the gene pathway associated with antigen presentation was also altered. Another pathway that showed increased expression included genes associated with LXR/RXR activation. This is presumably mediated by an increased production of metabolites that can function as ligands of LXR. This may have significant effects on a variety of metabolic processes and on the development of diabetes, both of which are associated with LXR activity [39]. Taken together, these results emphasize that the HFD had significant effects on various aspects of the immune system. It should be noted that the high-fat Western diet, which also reduced tumor latency and increased tumor multiplicity, failed to alter the same pathways observed with the HFD (Table 1). In figure 5, the relationship between the genes altered in a few canonical pathways identified in table 1 (molecular mechanisms of cancer, Wnt-βCatenin, LXR/RXR activation) is presented graphically. The genes altered in the molecular mechanism of cancer and the Wnt pathways have striking overlap as might be expected since the Wnt pathway is a major component of cancer development in multiple organs. In contrast, the LXR/RXR Pathway has no overlap with the Wnt pathway and modulation of only a single gene compared with molecular mechanisms of Cancer (Figure 5A). Parenthetically the two pathways in table 1 related to epithelial adherens junction signaling and molecular mechanisms of cancer, show few gene overlaps (Figure 5B) but all genes in the two pathways contribute to the process of “Cancer of Cells”. The contribution of many unrelated signaling pathways that converge to the cancer process supports our finding that a high fructose diet is contributing to increased tumor incidence and multiplicity in the current study.

There were two major reasons for performing metabolomics systematically in animals administered the two different diets. The first was to determine whether there were metabolic changes that might help to identify animals and potentially humans consuming higher levels of fructose. The other objective was to compare the metabolites in sera from rats on a HFD to those in the sera from rats on a high-fat diet [6]. There was minimal overlap between the metabolites altered by the two diets relative to the standard diet. However, we will discuss only a limited number of metabolic pathways for purely practical reasons and will present a more complete analysis in a separate publication. Furthermore, as mentioned above, we are only including those metabolites altered by the HF diet which were consistent at both time points (either increased or decreased at both time points and in virtually all cases statistically significant at both time points). Not unexpectedly, there were a wide range of metabolites which differed between serum derived from rats on a HFD and that derived from rats on a SD. Rats on a HFD had increased levels of fructose. Additionally, a variety of specific metabolites (e.g., soy products such as genistein and daidzein) associated with the SD, which employs soy protein as a protein source, were found at much lower levels in rodents on either the HFD or on the Western diet, which do not employ soy. One of the more important observations of these studies is that there were also multiple classes of metabolites that were altered in animals on the HFD, including reduced levels of diglycerols and certain oxygenated lipid mediators indicative of inflammation. Neither of these classes of metabolites were altered when comparing sera from rats on a Western diet and those on a SD. One might initially consider the decrease in oxygenated lipid mediators contrary to the expectation that a HFD will enhance inflammation. However, any clear interpretation of this result is complex since some of these metabolites may have pro-inflammatory or anti-inflammatory characteristics depending on cellular context. One additional metabolite class that is greatly increased in rats on the HFD, but no those on the Western diet, are the various primary and secondary bile acids (deoxycholate, hydrodeoxycholate, and 6- oxolithocholate). It is hypothesized that these metabolites may reflect metabolism by the gut microbiota. This result is particularly interesting since there is recent evidence to suggest that the anti-diabetic effects of metformin may be, in part, related to its ability to alter the microbiome and thus produce great alterations in bile acids [40]. In fact, we have observed those effects with metformin in rats on a HFD (unpublished data). Finally, there were metabolic changes in certain metabolites that have been shown to be associated with diabetes and insulin resistance, for example, increases in 2OH butyrate/2OH isobutyrate [41]. In addition, as presented in the supplementary tables, there were decreases in levels of sphingolipids and various benzoate-related metabolites (catechol sulfate and 4-vinyl phenol sulfate). One of the most important aspects of these studies is that at both time points (98 and 300 DOA), we observed alterations in multiple metabolites associated with a given metabolic pathway rather than changes in a variety of random metabolites in multiple pathways, implying that the pathway changes are real. The metabolites and metabolic pathways identified in this study may serve as part of a potential panel of markers to identify persons consuming higher levels of fructose.

## Conclusions and Possible Limitations

The studies presented in this manuscript yield a number of interesting findings and raise certain additional questions that should be considered in designing future studies. First, the results show that in the early (57 days old) exposure model of MNU-induced ER^+^ breast cancer, the use of an isocaloric HFD enhanced tumor development (shorter latency and greater tumor multiplicity) relative to the SD. This result is similar to what we reported in rats on a Western diet [7]. However, the Western diet was associated with a significant weight gain, which is routinely associated with more rapid tumor development. Parenthetically, certain of these prior studies added fructose in drinking water in addition to the standard diet resulting in substantial weight gain [16]. To avoid the confounding effects of a known contributor to the target cancer (weight gain) a nutritional approach was taken to use specific commercial diet sources to eliminate this variable. This approach makes the model system more generally applicable to its use as a screening method for preventive agents and in the long run more appropriate to human exposure since the effect on weight gain and obesity are well established. Since the rats only went on the HFD on day 50, and tumors started to develop in the HFD group by day 98, this implies that these physiologic changes that enhanced tumor growth occur rapidly. Second, the changes in RNA expression observed in tumors from the HFD appear to agree with the enhanced tumor development since we saw alterations in molecular cancer pathways including the Wnt pathways and pathways associated with metastasis. Additionally, immunologic pathways are particularly appealing for further analysis since the activation of the immune response (Th1 response, Th2 response, monocyte activation, antigen presentation) may have either positive or negative effects on tumor growth. Third, the differences in various serum metabolites and metabolic pathways in the HFD rats are of interest and consistent at both time points: Potentially the most striking findings are the strong increases in expression of various primary and secondary bile acids that are thought to contribute to diabetes through the alteration of FXR expression and appear to be modulated by metformin as part of its mechanism of action [17]. Additionally, there were a number of metabolites that appear to be related to a diabetic/prediabetic state, including an increase in 2OH butyrate. Particularly interesting in view of the alterations in immune pathways observed in the tumors from fructose exposed rats are the altered expression of certain inflammation-related metabolites. Some combination of these metabolites, in addition to fructose levels, may prove useful in identifying persons who have consumed higher levels of fructose. These preliminary results, especially in the area of metabolomics, suggest future experimental approaches *via* the use of genetic knock-in and knock-out *in vivo* models to provide more definitive “cause and effect” data regarding alterations in gene pathways that lead to enhanced tumorigenesis.

## Supplementary Material

Supplementary File

## Figures and Tables

**Table 1:** Canonical pathways altered in HFD condition.

Ingenuity canonical pathways	p- value (Fisher’s exact test )	p-value (B-H multiple testing correction	Molecules present (p-value < 0.05, FC 1.5)/ total molecule in pathway	Total molecules mapped/ present in pathway	Molecules
**Molecular Mechanisms of cancer**	2.8 × 10^−6^	2.1 × 10^−4^	37/388	225/388	RAC2, PIK3CA, PA2G4, NFKBIE, ADCY4, LRP6, RHOT2, NCSTN, **FZD1**, HIF1A, NFKB1, **CDK10**, TGFBR2, SHC1, ZBTB17, BMPR1A, TGFB1, HIPK2, RASA1, ATM, ITGB1, CCNE2, LRP5, PTCH1, SMAD6, SMAD7, APC, NF1, FOXO1, APH1A, RHOA, CDK11A, BMP7, JAK3, CTNND1, FZD7, PSEN1
**Epithelial adherens junction signaling**	8.3 × 10^−7^	1.3 × 10^−4^	21/150	86/150	SNAI2, MYH9, LMO7, ACTB, TGFBR3, **ACVR1**, IQGAP1, ACVR1B, APC, MET, TGFBR2, MYL9, NOTCH4, ACTR3, RHOA, BAIAP2, NECTIN1, ACVR2A, ACTN1, NECTIN2, CTNND1
**Regulation of the epithelial mesenchymal transition pathway**	6.1 × 10^−5^	3.0 × 10^−3^	21/193	91/193	PIK3CA, ID2, SNAI2, PARD6B, NCSTN, HIF1A, FZD1, ZEB1, NFKB1, APC, MET, TGFBR2, NOTCH4, TGFB1, APH1A, RHOA, JAK3, CLDN3, ATM, **FZD7**, PSEN1
**Wnt/β-catenin Signaling**	2.6 × 10^−4^	7.2 × 10^−3^	18/169	84/169	GJA1, LRP5, FRZB, CSNK1G2, TGFBR3, LRP6, **ACVR1**, FZD1, ACVR1B, APC, TGFBR2, CDH5, TGFB1, DKK3, PPM1L, UBC, ACVR2A, **FZD7**
**Th1 and Th2 activation pathway**	1.8 × 10^−6^	1.7 × 10^−4^	23/180		PIK3CA, ICAM1, HLA-A, IFNGR2, HLA-DQA1, NCSTN, NFKB1, ITGB2, LOC100909593/RT1-DMa, NOTCH4, LOC100909630/RT1-DMb, APH1A, HLA-DRA, JAK3, IFNAR1, HLA-DRB5, ATM, PSEN1
**Antigen presentation pathway**	2.7 × 10^−7^	1.2 × 10^−4^	Nov-38	17/38	B2M, CALR, HLA-A, PDIA3, HLA-DRA, HLA-DQA1, CANX, CD74, **TAPBP**, HLA-E, HLA-DRB5
**LXR/RXR activation**	1.5 × 10^−4^	5.1 × 10^−3^	15/121	55/121	APOE, SERPINF1, ABCG1, IRF3, PCYOX1, IL1R1, NFKB1, LYZ, SREBF1, IL1RN, **CD14**, TLR3, TNFRSF1B, CLU, TNFRSF11B

**Table 2:** Altered disease and function-related pathways.

Categories	Diseases or functions annotation	p-value	B-H p-value	Predicted activation state	# Molecules in the exp dataset	# of Genes that follow the direction
**Cellular movement, immune cell trafficking**	Leukocyte migration	5.78E-16	5.31E-13	Increased	114	65
**Hematological system development and function, lymphoid tissue structure and development, tissue morphology**	Quantity of T lymphocytes	6.09E-10	9.16E-08	Increased	67	39
**Cell death and survival**	Cell survival	2E-09	2.43E-07	Increased	135	89
**Cell death and survival**	Apoptosis	1.12E-13	4.56E-11	Decreased	237	113
**Cancer, organismal injury and abnormalities**	Epithelial neoplasm	4.44E-21	2.47E-17	Did not predict	754	NA
**Cancer, organismal injury and abnormalities**	Abdominal carcinoma	8.61E-19	1.27E-15	Did not predict	720	NA
**Cancer, organismal injury and abnormalities**	Cancer of cells	1.61E-13	4.50E-10	Increased	409	301

## Abbreviations

BH: Benjamini-Hochberg
DOA: Days of Age
DMBA: 7,12-dimethylbenz (a)anthracene
ER: Estrogen Receptor
FC: Fold-Change
FDR: False Discovery Rate
HFD: High-Fructose Diet
IPA: Ingenuity Pathway Analysis
MNU: N-methyl-N-nitrosourea
PR: Progesterone Receptor
SD: Standard Diet
SERM: Selective Estrogen Receptor Modulator
