# Supplementary material for: Effects of High-Fructose Diet vs. Teklad Diet in the MNU-Induced Rat Mammary Cancer Model: Altered Tumorigenesis, Metabolomics and Tumor RNA Expression
Source: J Obes Chronic Dis. Author manuscript; Available in PMC 2021 Apr 7. (PMC8026172)
Supplement: Supplementary File [file NIHMS1670153-supplement-Supplementary_File.docx]

**Figure S1:** Effect of HFD on tumor incidence and multiplicity in the late exposure protocol. Rats were placed on the HFD at 43 DOA and treated with MNU (50 mg/kg BW) at 100 DOA. Tumor incidence (A) and multiplicity (B) were determined twice per week by palpation.


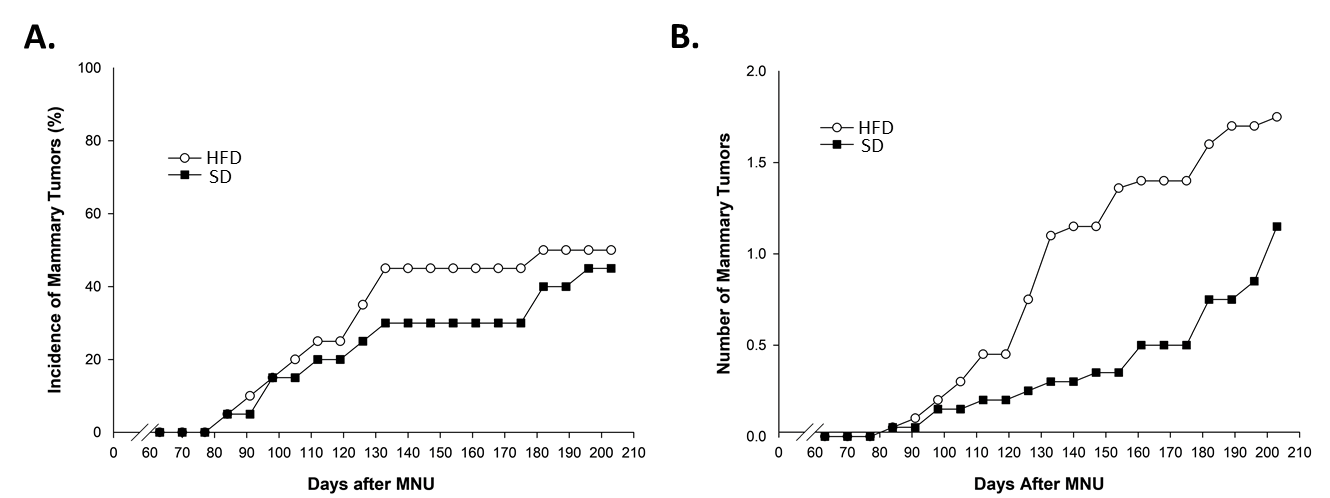


**Table S1: Components of SD and HFD**

| **Selected Nutrient % by weight** | **Teklad 7001 (4% fat)** | **Hi Fructose diet TD.89247** |
| --- | --- | --- |
| Protein | Primarily Soy 25.2% | Casein 18.3% |
| Carbohydrate (total) | 52.8% | 60.4% |
| Fat (lard) | 4.4% | 5.2% |
| Energy density | 3 Kcal/g | 3.6 Kcal/g |
| Calories from Protein | 34% | 20.2% |
| Calories from Fat | 14% | 13% |
| Calories from Carbohydrate | 53% | 66.8% |

**Table S2: Relative levels of serum metabolites at 98 and 300 DOA**

|  | **Relative Metabolite Levels (HFD/SD)** | **98 DOA** | **300 DOA** |
| --- | --- | --- | --- |
| **Food and Plant Components (*Known Soy Component)** | Genistein* | 0.1 | 0.12 |
|  | 2,8-quinolinediol sulfate | 0.66 | 0.3 |
|  | 3-hydroxyindolin-2-one | 0.95 | 1.34 |
|  | betonicine | 0.36 | 0.41 |
|  | gluconate | 0.71 | 1.15 |
|  | Daidzein* | 0.16 | 0.17 |
|  | Equol* | 0.47 | 0.32 |
|  | equol glucuronide* | 0.4 | 0.42 |
|  | equol sulfate* | 0.83 | 0.67 |
|  | ergothioneine | 0.06 | 0.02 |
|  | erythritol | 2.74 | 3.6 |
|  | homostachydrine* | 0 | 0.01 |
|  | N-glycolylneuraminate | 0.87 | 1.31 |
|  | stachydrine | 0 | 0.01 |
| **Diacylglycerols** | linoleoyl-arachidonoyl-glycerol (18:2/20:4) | 0.2 | 0.14 |
|  | linoleoyl-arachidonoyl-glycerol (18:2/20:4) | 0.16 | 0.16 |
|  | linoleoyl-linoleoyl-glycerol (18:2/18:2) | 0.09 | 0.07 |
|  | linoleoyl-linolenoyl-glycerol (18:2/18:3) | 0.09 | 0.11 |
|  | linoleoyl-docosahexaenoyl-glycerol (18:2/22:6) | 0.5 | 0.48 |
|  | palmitoyl-arachidonoyl-glycerol (16:0/20:4) | 1.82 | 1.82 |
| **Polyunsaturated**  **Fatty Acids (n3 and n6)** | stearidonate (18:4n3) | 0.11 | 0.15 |
|  | eicosapentaenoate (EPA; 20:5n3) | 0.6 | 0.51 |
|  | docosapentaenoate (n3 DPA; 22:5n3) | 0.4 | 0.32 |
|  | docosahexaenoate (DHA; 22:6n3) | 1.23 | 1.17 |
|  | linoleate (18:2n6) | 0.34 | 0.31 |
|  | linolenate [alpha or gamma; (18:3n3 or 6)] | 0.18 | 0.18 |
| **Secondary Bile Acids** | deoxycholate | 1.47 | 5.97 |
|  | glycodeoxycholate | 0.4 | 9.53 |
|  | taurodeoxycholate | 0.43 | 1.56 |
|  | ursodeoxycholate | 1.4 | 9.44 |
|  | 6-oxolithocholate | 2.84 | 15.55 |
|  | hyocholate | 3.52 | 5.97 |
|  | hyodeoxycholate | 4.99 | 12.35 |
|  | taurohyodeoxycholic acid | 0.66 | 1.62 |
| **Benzoate Metabolites** | catechol sulfate | 0.06 | 0.03 |
|  | O-methylcatechol sulfate | 0.24 | 0.16 |
|  | 4-methylcatechol sulfate | 0.4 | 0.86 |
|  | 4-ethylphenylsulfate | 0 | 0 |
|  | 4-vinylphenol sulfate | 0.17 | 0.07 |

Higher in the HFD rats (P<0.05). Lower in the HFD rats (P<0.05). Not significantly different in HFD vs. SD.

**Table S3: Genes identified using less stringent conditions (HFD vs. SD)**

| **Gene ID** | **Transcript ID** | **Total counts** | **P-value** | **FDR step up** | **Ratio** | **Fold change** | **LSMean**  **(HFD)** | **LSMean**  **(SD)** |
| --- | --- | --- | --- | --- | --- | --- | --- | --- |
| Csnk1g2 | NM_023102 | 6.06E+02 | 7.56E-04 | 7.12E-02 | 2.27E-01 | -4.40E+00 | 1.99E+01 | 8.77E+01 |
| Rn45s | NR_046239 | 9.73E+03 | 4.15E-03 | 1.19E-01 | 2.45E-01 | -4.08E+00 | 3.42E+02 | 1.39E+03 |
| Abcg3l2 | NM_001014133 | 8.93E+02 | 1.12E-06 | 9.63E-03 | 2.61E-01 | -3.83E+00 | 3.31E+01 | 1.27E+02 |
| Rn18s | NR_046237 | 1.76E+05 | 1.59E-03 | 8.75E-02 | 2.66E-01 | -3.76E+00 | 6.62E+03 | 2.49E+04 |
| LOC257642 | NM_147136 | 3.52E+04 | 4.19E-04 | 6.47E-02 | 2.70E-01 | -3.70E+00 | 1.34E+03 | 4.97E+03 |
| Rn28s | NR_046246 | 3.84E+05 | 6.79E-03 | 1.30E-01 | 2.78E-01 | -3.60E+00 | 1.50E+04 | 5.39E+04 |
| Inafm1 | NM_001136272 | 7.07E+02 | 1.01E-04 | 6.47E-02 | 2.82E-01 | -3.54E+00 | 2.80E+01 | 9.91E+01 |
| Pabpn1 | NM_001135008 | 4.64E+03 | 3.07E-03 | 1.10E-01 | 3.25E-01 | -3.08E+00 | 2.07E+02 | 6.36E+02 |
| Akap8l | NM_001013946 | 1.49E+03 | 2.23E-04 | 6.47E-02 | 3.55E-01 | -2.81E+00 | 7.15E+01 | 2.01E+02 |
| Cdk10 | NM_001109937 | 5.15E+02 | 2.62E-04 | 6.47E-02 | 3.62E-01 | -2.76E+00 | 2.50E+01 | 6.91E+01 |
| Sec62 | NM_001034129 | 1.20E+03 | 2.02E-02 | 1.87E-01 | 3.68E-01 | -2.72E+00 | 5.89E+01 | 1.60E+02 |
| Acsm3 | NM_033231 | 3.35E+02 | 3.75E-04 | 6.47E-02 | 3.71E-01 | -2.70E+00 | 1.66E+01 | 4.48E+01 |
| Catsperg | NM_001170340 | 2.33E+02 | 5.43E-03 | 1.25E-01 | 4.00E-01 | -2.50E+00 | 1.22E+01 | 3.06E+01 |
| Ctnnbl1 | NM_001024870 | 8.17E+02 | 1.18E-02 | 1.51E-01 | 4.07E-01 | -2.46E+00 | 4.36E+01 | 1.07E+02 |
| Il3ra | NM_139260 | 5.58E+02 | 8.99E-05 | 6.47E-02 | 4.15E-01 | -2.41E+00 | 3.03E+01 | 7.29E+01 |
| Hipk2 | NM_001108622 | 2.14E+02 | 4.59E-03 | 1.20E-01 | 4.15E-01 | -2.41E+00 | 1.16E+01 | 2.79E+01 |
| Nxf1 | NM_021579 | 2.65E+03 | 2.66E-04 | 6.47E-02 | 4.18E-01 | -2.39E+00 | 1.45E+02 | 3.46E+02 |
| Cdk10 | NM_001109936 | 2.42E+02 | 3.81E-03 | 1.17E-01 | 4.21E-01 | -2.38E+00 | 1.32E+01 | 3.15E+01 |
| LOC310926 | NM_001025002 | 4.16E+02 | 6.64E-03 | 1.29E-01 | 4.24E-01 | -2.36E+00 | 2.29E+01 | 5.41E+01 |
| Zfp148 | NM_031615 | 4.27E+02 | 5.36E-03 | 1.24E-01 | 4.24E-01 | -2.36E+00 | 2.35E+01 | 5.55E+01 |
| Cchcr1 | NM_001002822 | 5.18E+02 | 2.07E-03 | 9.87E-02 | 4.33E-01 | -2.31E+00 | 2.90E+01 | 6.70E+01 |
| Mapt | NM_017212 | 1.38E+02 | 8.75E-04 | 7.65E-02 | 4.39E-01 | -2.28E+00 | 7.79E+00 | 1.77E+01 |
| Atg16l2 | NM_001191560 | 1.51E+02 | 2.20E-04 | 6.47E-02 | 4.46E-01 | -2.24E+00 | 8.66E+00 | 1.94E+01 |
| Uspl1 | NM_001105906 | 1.97E+02 | 2.03E-04 | 6.47E-02 | 4.48E-01 | -2.23E+00 | 1.13E+01 | 2.53E+01 |
| Tnrc6a | NM_001107549 | 1.18E+03 | 1.14E-03 | 8.00E-02 | 4.51E-01 | -2.22E+00 | 6.83E+01 | 1.51E+02 |
| Tle2 | NM_001039013 | 5.92E+02 | 3.36E-05 | 6.47E-02 | 4.51E-01 | -2.21E+00 | 3.43E+01 | 7.59E+01 |
| Vill | NM_001191617 | 9.54E+01 | 4.33E-04 | 6.47E-02 | 4.53E-01 | -2.21E+00 | 5.53E+00 | 1.22E+01 |
| Akap17a | NM_001127246 | 1.39E+03 | 3.85E-04 | 6.47E-02 | 4.56E-01 | -2.19E+00 | 8.11E+01 | 1.78E+02 |
| Rgl3 | NM_001106805 | 8.69E+01 | 8.94E-03 | 1.38E-01 | 4.57E-01 | -2.19E+00 | 5.07E+00 | 1.11E+01 |
| Hdac10 | NM_001035000 | 2.65E+02 | 7.06E-04 | 6.85E-02 | 4.58E-01 | -2.18E+00 | 1.55E+01 | 3.38E+01 |
| Il17rc | NM_001170565 | 5.72E+02 | 1.96E-03 | 9.67E-02 | 4.58E-01 | -2.18E+00 | 3.35E+01 | 7.31E+01 |
| Safb2 | NM_001303144 | 1.04E+03 | 5.28E-05 | 6.47E-02 | 4.62E-01 | -2.16E+00 | 6.13E+01 | 1.33E+02 |
| RGD1562114 | NM_001109283 | 4.60E+02 | 2.95E-04 | 6.47E-02 | 4.69E-01 | -2.13E+00 | 2.74E+01 | 5.84E+01 |
| Miip | NM_001017450 | 1.60E+02 | 5.16E-04 | 6.47E-02 | 4.69E-01 | -2.13E+00 | 9.52E+00 | 2.03E+01 |
| Miip | NM_001017450.1 | 1.60E+02 | 5.16E-04 | 6.47E-02 | 4.69E-01 | -2.13E+00 | 9.52E+00 | 2.03E+01 |
| Elmo3 | NM_001030028 | 5.93E+02 | 6.61E-04 | 6.85E-02 | 4.77E-01 | -2.10E+00 | 3.57E+01 | 7.50E+01 |
| Cep95 | NM_001013862 | 6.04E+02 | 1.66E-04 | 6.47E-02 | 4.77E-01 | -2.10E+00 | 3.64E+01 | 7.63E+01 |
| Haus7 | NM_001277272 | 2.73E+02 | 3.65E-04 | 6.47E-02 | 4.78E-01 | -2.09E+00 | 1.65E+01 | 3.45E+01 |
| Phc3 | NM_001107662 | 4.97E+01 | 6.06E-03 | 1.26E-01 | 4.79E-01 | -2.09E+00 | 3.01E+00 | 6.28E+00 |
| Lrrc14 | NM_001024354 | 3.14E+02 | 8.08E-04 | 7.42E-02 | 4.82E-01 | -2.07E+00 | 1.91E+01 | 3.96E+01 |
| Chrne | NM_017194 | 4.87E+01 | 7.23E-03 | 1.32E-01 | 4.85E-01 | -2.06E+00 | 2.98E+00 | 6.14E+00 |
| Abcb6 | NM_080582 | 5.93E+02 | 4.11E-04 | 6.47E-02 | 4.90E-01 | -2.04E+00 | 3.65E+01 | 7.45E+01 |
| Coq8b | NM_001012065 | 3.23E+02 | 1.99E-03 | 9.69E-02 | 4.95E-01 | -2.02E+00 | 2.01E+01 | 4.05E+01 |
| Traf3ip2 | NM_001044248 | 6.25E+02 | 2.55E-03 | 1.05E-01 | 4.97E-01 | -2.01E+00 | 3.89E+01 | 7.82E+01 |
| Clasrp | NM_001024294 | 5.97E+02 | 3.22E-04 | 6.47E-02 | 5.03E-01 | -1.99E+00 | 3.75E+01 | 7.45E+01 |
| Dkc1 | NM_133419 | 1.23E+03 | 1.40E-03 | 8.31E-02 | 5.04E-01 | -1.99E+00 | 7.70E+01 | 1.53E+02 |
| Spata7 | NM_138862 | 2.23E+02 | 3.93E-04 | 6.47E-02 | 5.05E-01 | -1.98E+00 | 1.40E+01 | 2.78E+01 |
| Nosip | NM_001106260 | 6.66E+02 | 2.77E-04 | 6.47E-02 | 5.06E-01 | -1.98E+00 | 4.20E+01 | 8.31E+01 |
| Abca7 | NM_207598 | 1.43E+02 | 1.05E-03 | 7.92E-02 | 5.06E-01 | -1.98E+00 | 9.04E+00 | 1.79E+01 |
| Tra2a | NM_001126296 | 2.12E+03 | 2.59E-04 | 6.47E-02 | 5.08E-01 | -1.97E+00 | 1.34E+02 | 2.64E+02 |
| Mmgt2 | NM_001013967 | 4.09E+02 | 6.05E-04 | 6.62E-02 | 5.10E-01 | -1.96E+00 | 2.59E+01 | 5.09E+01 |
| Trit1 | NM_001108676 | 5.32E+02 | 2.44E-04 | 6.47E-02 | 5.13E-01 | -1.95E+00 | 3.39E+01 | 6.61E+01 |
| Egfl7 | NM_139104 | 1.46E+03 | 2.19E-03 | 9.95E-02 | 5.13E-01 | -1.95E+00 | 9.33E+01 | 1.82E+02 |
| Ssna1 | NR_130647 | 7.27E+01 | 1.08E-02 | 1.45E-01 | 5.14E-01 | -1.94E+00 | 4.64E+00 | 9.02E+00 |
| Zfp653 | NM_001106807 | 1.63E+02 | 1.89E-03 | 9.66E-02 | 5.15E-01 | -1.94E+00 | 1.04E+01 | 2.03E+01 |
| Zfyve19 | NM_001034948 | 2.75E+02 | 3.40E-03 | 1.12E-01 | 5.15E-01 | -1.94E+00 | 1.76E+01 | 3.41E+01 |
| Kank3 | NM_001108989 | 6.53E+02 | 8.28E-04 | 7.42E-02 | 5.17E-01 | -1.94E+00 | 4.18E+01 | 8.10E+01 |
| Polr2h | NM_001134789.1 | 1.15E+02 | 9.81E-03 | 1.43E-01 | 5.17E-01 | -1.93E+00 | 7.36E+00 | 1.42E+01 |
| Polr2h | NM_001134789 | 1.15E+02 | 9.81E-03 | 1.43E-01 | 5.17E-01 | -1.93E+00 | 7.36E+00 | 1.42E+01 |
| Tssc4 | NM_001310046 | 1.42E+02 | 4.31E-03 | 1.19E-01 | 5.19E-01 | -1.93E+00 | 9.14E+00 | 1.76E+01 |
| Zufsp | NM_001008308 | 3.56E+02 | 1.43E-04 | 6.47E-02 | 5.19E-01 | -1.93E+00 | 2.29E+01 | 4.40E+01 |
| Tpcn2 | NM_001107566 | 3.91E+02 | 1.57E-04 | 6.47E-02 | 5.22E-01 | -1.92E+00 | 2.52E+01 | 4.83E+01 |
| Tmem120a | NM_001010945 | 1.10E+03 | 8.31E-04 | 7.42E-02 | 5.22E-01 | -1.92E+00 | 7.11E+01 | 1.36E+02 |
| Mtif2 | NM_001004254 | 3.20E+02 | 5.75E-04 | 6.47E-02 | 5.25E-01 | -1.91E+00 | 2.07E+01 | 3.95E+01 |
| Pik3ca | NM_133399 | 1.73E+03 | 1.38E-03 | 8.31E-02 | 5.26E-01 | -1.90E+00 | 1.12E+02 | 2.14E+02 |
| Pex11a | NM_053487 | 6.59E+02 | 1.22E-02 | 1.53E-01 | 5.26E-01 | -1.90E+00 | 4.28E+01 | 8.13E+01 |
| Kmt5c | NM_001107475 | 8.16E+02 | 1.36E-03 | 8.31E-02 | 5.28E-01 | -1.90E+00 | 5.31E+01 | 1.01E+02 |
| Deaf1 | NM_031801 | 1.15E+03 | 3.06E-04 | 6.47E-02 | 5.28E-01 | -1.89E+00 | 7.52E+01 | 1.42E+02 |
| Adcy4 | NM_019285 | 2.91E+02 | 5.44E-03 | 1.25E-01 | 5.33E-01 | -1.88E+00 | 1.91E+01 | 3.58E+01 |
| Cyp4f6 | NM_153318 | 2.93E+02 | 2.95E-03 | 1.10E-01 | 5.35E-01 | -1.87E+00 | 1.93E+01 | 3.60E+01 |
| Rpap3 | NM_001004243 | 7.16E+02 | 2.03E-04 | 6.47E-02 | 5.38E-01 | -1.86E+00 | 4.72E+01 | 8.78E+01 |
| Hexdc | NM_001142562 | 5.35E+02 | 7.27E-03 | 1.32E-01 | 5.38E-01 | -1.86E+00 | 3.53E+01 | 6.56E+01 |
| Cc2d1a | NM_001013869 | 7.65E+02 | 1.97E-03 | 9.67E-02 | 5.39E-01 | -1.85E+00 | 5.06E+01 | 9.38E+01 |
| Clk2 | NM_001014254 | 8.59E+02 | 5.74E-04 | 6.47E-02 | 5.40E-01 | -1.85E+00 | 5.69E+01 | 1.05E+02 |
| Pnpla7 | NM_144738 | 1.13E+03 | 5.38E-03 | 1.24E-01 | 5.41E-01 | -1.85E+00 | 7.47E+01 | 1.38E+02 |
| Zfp775 | NM_001107859 | 2.14E+02 | 5.48E-04 | 6.47E-02 | 5.42E-01 | -1.85E+00 | 1.42E+01 | 2.62E+01 |
| Wdr83 | NM_001047847 | 2.46E+02 | 2.58E-04 | 6.47E-02 | 5.43E-01 | -1.84E+00 | 1.63E+01 | 3.01E+01 |
| Gpatch4 | NM_001024979 | 2.92E+02 | 7.23E-03 | 1.32E-01 | 5.44E-01 | -1.84E+00 | 1.94E+01 | 3.57E+01 |
| Tbce | NM_001012161 | 6.72E+02 | 2.18E-04 | 6.47E-02 | 5.45E-01 | -1.83E+00 | 4.48E+01 | 8.22E+01 |
| Rhpn1 | NM_001305237 | 3.65E+01 | 6.10E-03 | 1.27E-01 | 5.48E-01 | -1.83E+00 | 2.44E+00 | 4.45E+00 |
| Jak3 | NM_012855 | 3.95E+02 | 4.42E-03 | 1.19E-01 | 5.48E-01 | -1.82E+00 | 2.64E+01 | 4.82E+01 |
| Ilkap | NM_022606 | 6.84E+02 | 8.54E-04 | 7.55E-02 | 5.49E-01 | -1.82E+00 | 4.58E+01 | 8.35E+01 |
| Zbtb17 | NM_001012105 | 3.77E+02 | 5.47E-03 | 1.25E-01 | 5.52E-01 | -1.81E+00 | 2.53E+01 | 4.59E+01 |
| Abtb1 | NM_001005902 | 3.65E+02 | 1.96E-03 | 9.67E-02 | 5.52E-01 | -1.81E+00 | 2.46E+01 | 4.45E+01 |
| Slfn4 | NM_053687 | 5.78E+02 | 2.24E-03 | 9.95E-02 | 5.52E-01 | -1.81E+00 | 3.89E+01 | 7.05E+01 |
| Phf7 | NM_001012211 | 2.26E+02 | 9.31E-03 | 1.41E-01 | 5.53E-01 | -1.81E+00 | 1.52E+01 | 2.75E+01 |
| Arv1 | NM_001106197 | 2.59E+02 | 5.04E-03 | 1.24E-01 | 5.54E-01 | -1.81E+00 | 1.75E+01 | 3.15E+01 |
| Chkb | NM_017177 | 5.82E+02 | 7.51E-04 | 7.12E-02 | 5.54E-01 | -1.80E+00 | 3.92E+01 | 7.08E+01 |
| Dus2 | NM_001106181 | 2.40E+02 | 2.34E-03 | 1.00E-01 | 5.56E-01 | -1.80E+00 | 1.62E+01 | 2.92E+01 |
| Clk1 | NM_001106913 | 4.49E+03 | 9.97E-03 | 1.43E-01 | 5.57E-01 | -1.80E+00 | 3.04E+02 | 5.46E+02 |
| Rps6kb2 | NM_001010962 | 3.68E+02 | 4.54E-04 | 6.47E-02 | 5.57E-01 | -1.79E+00 | 2.49E+01 | 4.47E+01 |
| Pla2g4b | NM_001107764 | 1.37E+02 | 3.88E-03 | 1.17E-01 | 5.58E-01 | -1.79E+00 | 9.30E+00 | 1.67E+01 |
| Prickle3 | NM_001014110 | 3.33E+02 | 1.15E-03 | 8.04E-02 | 5.58E-01 | -1.79E+00 | 2.26E+01 | 4.05E+01 |
| Zc3h7a | NM_001108262 | 1.39E+03 | 2.87E-04 | 6.47E-02 | 5.59E-01 | -1.79E+00 | 9.41E+01 | 1.68E+02 |
| Fuz | NM_001037646 | 2.94E+02 | 2.38E-04 | 6.47E-02 | 5.63E-01 | -1.78E+00 | 2.01E+01 | 3.57E+01 |
| Coq10a | NM_001108727 | 2.97E+02 | 7.02E-04 | 6.85E-02 | 5.64E-01 | -1.77E+00 | 2.03E+01 | 3.60E+01 |
| Lppos | NR_132630 | 1.03E+02 | 1.25E-02 | 1.55E-01 | 5.64E-01 | -1.77E+00 | 7.03E+00 | 1.25E+01 |
| Acin1 | NM_001170468 | 2.21E+03 | 1.21E-03 | 8.06E-02 | 5.64E-01 | -1.77E+00 | 1.51E+02 | 2.67E+02 |
| Pde2a | NM_001270604 | 3.65E+02 | 1.55E-02 | 1.69E-01 | 5.68E-01 | -1.76E+00 | 2.51E+01 | 4.42E+01 |
| Rhot2 | NM_181823 | 6.87E+02 | 9.92E-04 | 7.90E-02 | 5.68E-01 | -1.76E+00 | 4.72E+01 | 8.31E+01 |
| Armc5 | NM_001009455 | 7.21E+02 | 5.78E-04 | 6.47E-02 | 5.70E-01 | -1.75E+00 | 4.97E+01 | 8.71E+01 |
| Exoc3l1 | NM_001106178 | 1.69E+02 | 2.38E-04 | 6.47E-02 | 5.72E-01 | -1.75E+00 | 1.16E+01 | 2.03E+01 |
| Sirt5 | NM_001004256 | 6.92E+02 | 1.48E-03 | 8.43E-02 | 5.73E-01 | -1.74E+00 | 4.79E+01 | 8.35E+01 |
| Ccne2 | NM_001108656 | 2.68E+02 | 1.40E-03 | 8.31E-02 | 5.74E-01 | -1.74E+00 | 1.86E+01 | 3.24E+01 |
| Dhx57 | NM_001191907 | 4.73E+02 | 2.28E-03 | 9.95E-02 | 5.78E-01 | -1.73E+00 | 3.29E+01 | 5.69E+01 |
| Tmem265 | NM_001304268 | 2.73E+02 | 1.34E-03 | 8.31E-02 | 5.80E-01 | -1.72E+00 | 1.90E+01 | 3.28E+01 |
| Myo9b | NM_012984 | 4.89E+02 | 2.01E-03 | 9.75E-02 | 5.81E-01 | -1.72E+00 | 3.41E+01 | 5.88E+01 |
| Tada2a | NM_001012141 | 4.71E+02 | 5.62E-04 | 6.47E-02 | 5.82E-01 | -1.72E+00 | 3.29E+01 | 5.65E+01 |
| Notch4 | NM_001002827 | 8.47E+02 | 5.51E-03 | 1.25E-01 | 5.82E-01 | -1.72E+00 | 5.92E+01 | 1.02E+02 |
| Gba2 | NM_001013091 | 5.16E+02 | 6.93E-03 | 1.31E-01 | 5.82E-01 | -1.72E+00 | 3.61E+01 | 6.20E+01 |
| Parp6 | NM_001106828 | 5.56E+02 | 5.25E-03 | 1.24E-01 | 5.83E-01 | -1.72E+00 | 3.89E+01 | 6.68E+01 |
| Fam126b | NM_001025710 | 1.68E+02 | 1.23E-03 | 8.06E-02 | 5.83E-01 | -1.72E+00 | 1.17E+01 | 2.01E+01 |
| Rbm43 | NM_001037649 | 1.35E+02 | 7.81E-03 | 1.32E-01 | 5.83E-01 | -1.72E+00 | 9.44E+00 | 1.62E+01 |
| Mafg | NM_022386 | 1.79E+02 | 3.13E-03 | 1.10E-01 | 5.83E-01 | -1.72E+00 | 1.25E+01 | 2.15E+01 |
| Mier2 | NM_001108737 | 4.79E+02 | 9.54E-03 | 1.42E-01 | 5.83E-01 | -1.72E+00 | 3.35E+01 | 5.75E+01 |
| Lpin3 | NM_001014184 | 5.65E+02 | 3.88E-03 | 1.17E-01 | 5.85E-01 | -1.71E+00 | 3.96E+01 | 6.78E+01 |
| Rasip1 | NM_001106261 | 9.11E+02 | 5.22E-03 | 1.24E-01 | 5.87E-01 | -1.70E+00 | 6.40E+01 | 1.09E+02 |
| Zfp513 | NM_001012110 | 7.62E+02 | 1.45E-03 | 8.35E-02 | 5.88E-01 | -1.70E+00 | 5.37E+01 | 9.12E+01 |
| Cenpj | NM_001107265 | 3.63E+02 | 2.58E-04 | 6.47E-02 | 5.89E-01 | -1.70E+00 | 2.56E+01 | 4.34E+01 |
| Mib2 | NM_001005901 | 3.90E+02 | 1.44E-03 | 8.33E-02 | 5.91E-01 | -1.69E+00 | 2.75E+01 | 4.66E+01 |
| Glmn | NM_001105993 | 1.92E+02 | 1.28E-03 | 8.26E-02 | 5.92E-01 | -1.69E+00 | 1.36E+01 | 2.30E+01 |
| Pex6 | NM_057125 | 5.55E+02 | 7.17E-03 | 1.32E-01 | 5.92E-01 | -1.69E+00 | 3.93E+01 | 6.63E+01 |
| Npdc1 | NM_001004231 | 1.88E+03 | 1.17E-02 | 1.51E-01 | 5.95E-01 | -1.68E+00 | 1.33E+02 | 2.24E+02 |
| Exosc8 | NM_001106432 | 2.29E+02 | 2.93E-03 | 1.10E-01 | 5.95E-01 | -1.68E+00 | 1.63E+01 | 2.73E+01 |
| Ccdc22 | NM_001135837 | 3.54E+02 | 3.62E-03 | 1.17E-01 | 5.97E-01 | -1.68E+00 | 2.52E+01 | 4.22E+01 |
| Trim39 | NM_213562 | 3.97E+02 | 2.65E-03 | 1.07E-01 | 5.97E-01 | -1.68E+00 | 2.82E+01 | 4.73E+01 |
| Ccnl1 | NM_053662 | 1.35E+03 | 6.02E-03 | 1.26E-01 | 5.97E-01 | -1.68E+00 | 9.59E+01 | 1.61E+02 |
| Slc26a6 | NM_001143817 | 2.46E+02 | 1.91E-03 | 9.67E-02 | 5.99E-01 | -1.67E+00 | 1.76E+01 | 2.93E+01 |
| Nufip1 | NM_001007758 | 3.71E+02 | 1.14E-02 | 1.49E-01 | 5.99E-01 | -1.67E+00 | 2.65E+01 | 4.42E+01 |
| Tbkbp1 | NM_172021 | 3.28E+02 | 9.56E-03 | 1.42E-01 | 6.03E-01 | -1.66E+00 | 2.35E+01 | 3.90E+01 |
| Atg4d | NM_001101013 | 5.16E+02 | 6.66E-03 | 1.29E-01 | 6.04E-01 | -1.66E+00 | 3.70E+01 | 6.13E+01 |
| Zmym6 | NM_001108681 | 5.40E+02 | 4.43E-03 | 1.19E-01 | 6.06E-01 | -1.65E+00 | 3.89E+01 | 6.41E+01 |
| Phkg2 | NM_080584 | 7.02E+02 | 4.03E-03 | 1.18E-01 | 6.07E-01 | -1.65E+00 | 5.06E+01 | 8.33E+01 |
| Sfswap | NM_001034924 | 4.63E+02 | 2.47E-03 | 1.03E-01 | 6.08E-01 | -1.64E+00 | 3.34E+01 | 5.49E+01 |
| Fam193b | NM_001170408 | 8.17E+02 | 3.36E-03 | 1.12E-01 | 6.10E-01 | -1.64E+00 | 5.91E+01 | 9.68E+01 |
| Pde7a | NM_031080 | 2.85E+02 | 5.93E-03 | 1.26E-01 | 6.11E-01 | -1.64E+00 | 2.06E+01 | 3.38E+01 |
| Commd4 | NM_001108762 | 7.80E+02 | 2.14E-03 | 9.95E-02 | 6.12E-01 | -1.63E+00 | 5.65E+01 | 9.23E+01 |
| Hnrnph1 | NM_080896 | 5.55E+03 | 2.50E-03 | 1.03E-01 | 6.14E-01 | -1.63E+00 | 4.03E+02 | 6.57E+02 |
| Fastkd2 | NM_001009673 | 1.40E+02 | 6.60E-03 | 1.29E-01 | 6.14E-01 | -1.63E+00 | 1.02E+01 | 1.66E+01 |
| Ybx1 | NM_031563 | 7.21E+02 | 1.34E-02 | 1.59E-01 | 6.14E-01 | -1.63E+00 | 5.23E+01 | 8.52E+01 |
| Shank3 | NM_021676 | 8.54E+02 | 7.69E-03 | 1.32E-01 | 6.14E-01 | -1.63E+00 | 6.20E+01 | 1.01E+02 |
| Clk3 | NM_134340 | 8.31E+02 | 9.80E-04 | 7.90E-02 | 6.14E-01 | -1.63E+00 | 6.03E+01 | 9.82E+01 |
| Rbm39 | NM_001013207 | 2.89E+03 | 3.20E-03 | 1.10E-01 | 6.14E-01 | -1.63E+00 | 2.10E+02 | 3.41E+02 |
| Rbm39 | NM_001013207.1 | 2.89E+03 | 3.20E-03 | 1.10E-01 | 6.14E-01 | -1.63E+00 | 2.10E+02 | 3.41E+02 |
| Pstk | NM_001271229 | 2.71E+02 | 1.32E-03 | 8.31E-02 | 6.16E-01 | -1.62E+00 | 1.98E+01 | 3.21E+01 |
| Tars2 | NM_001014040 | 3.64E+02 | 2.98E-03 | 1.10E-01 | 6.17E-01 | -1.62E+00 | 2.65E+01 | 4.30E+01 |
| Pla2r1 | NM_001100837 | 1.40E+02 | 7.16E-03 | 1.32E-01 | 6.17E-01 | -1.62E+00 | 1.02E+01 | 1.66E+01 |
| Fhod1 | NM_001191600 | 5.24E+02 | 2.07E-02 | 1.90E-01 | 6.20E-01 | -1.61E+00 | 3.83E+01 | 6.18E+01 |
| Trmt13 | NM_001033902 | 1.61E+02 | 4.43E-03 | 1.19E-01 | 6.20E-01 | -1.61E+00 | 1.18E+01 | 1.90E+01 |
| Zfp202 | NM_001109290 | 2.29E+02 | 1.03E-02 | 1.44E-01 | 6.21E-01 | -1.61E+00 | 1.68E+01 | 2.70E+01 |
| Mpnd | NM_001085406 | 7.27E+02 | 4.92E-04 | 6.47E-02 | 6.23E-01 | -1.61E+00 | 5.33E+01 | 8.57E+01 |
| Egln2 | NM_001004083 | 1.20E+03 | 6.87E-03 | 1.31E-01 | 6.23E-01 | -1.61E+00 | 8.78E+01 | 1.41E+02 |
| Zeb1 | NM_001308265 | 8.96E+02 | 2.79E-03 | 1.08E-01 | 6.23E-01 | -1.60E+00 | 6.57E+01 | 1.05E+02 |
| Cops4 | NM_001004275 | 1.07E+03 | 6.31E-03 | 1.28E-01 | 6.24E-01 | -1.60E+00 | 7.89E+01 | 1.26E+02 |
| Cttnbp2 | NM_001114401 | 1.35E+02 | 1.11E-02 | 1.47E-01 | 6.24E-01 | -1.60E+00 | 9.91E+00 | 1.59E+01 |
| Samd11 | NM_001305446 | 6.29E+02 | 2.81E-03 | 1.08E-01 | 6.25E-01 | -1.60E+00 | 4.62E+01 | 7.40E+01 |
| Wsb1 | NM_001025664 | 9.83E+02 | 1.67E-02 | 1.75E-01 | 6.25E-01 | -1.60E+00 | 7.23E+01 | 1.16E+02 |
| Gramd1a | NM_001014160 | 8.61E+02 | 6.13E-03 | 1.27E-01 | 6.26E-01 | -1.60E+00 | 6.34E+01 | 1.01E+02 |
| Dnm1 | NM_080689 | 3.77E+02 | 5.07E-03 | 1.24E-01 | 6.26E-01 | -1.60E+00 | 2.78E+01 | 4.44E+01 |
| Trim34 | NM_001276491 | 4.61E+02 | 5.31E-03 | 1.24E-01 | 6.27E-01 | -1.59E+00 | 3.40E+01 | 5.42E+01 |
| Trafd1 | NM_053760 | 1.35E+03 | 3.51E-03 | 1.14E-01 | 6.29E-01 | -1.59E+00 | 9.96E+01 | 1.59E+02 |
| Tsen54 | NM_001109576 | 1.63E+02 | 7.29E-03 | 1.32E-01 | 6.29E-01 | -1.59E+00 | 1.20E+01 | 1.92E+01 |
| Slc35e4 | NM_153316 | 4.00E+02 | 2.12E-02 | 1.91E-01 | 6.29E-01 | -1.59E+00 | 2.96E+01 | 4.70E+01 |
| Akap8 | NM_053855 | 2.00E+03 | 5.78E-03 | 1.26E-01 | 6.29E-01 | -1.59E+00 | 1.48E+02 | 2.35E+02 |
| Ift88 | NM_001107266 | 1.67E+02 | 1.97E-02 | 1.86E-01 | 6.29E-01 | -1.59E+00 | 1.23E+01 | 1.96E+01 |
| Coq5 | NM_001039022 | 2.94E+02 | 3.37E-04 | 6.47E-02 | 6.30E-01 | -1.59E+00 | 2.17E+01 | 3.45E+01 |
| Sema6b | NM_053471 | 3.28E+02 | 2.27E-02 | 1.97E-01 | 6.31E-01 | -1.59E+00 | 2.43E+01 | 3.85E+01 |
| Rfesd | NM_001108540 | 8.02E+01 | 3.07E-03 | 1.10E-01 | 6.32E-01 | -1.58E+00 | 5.95E+00 | 9.40E+00 |
| Tnk2 | NM_001008336 | 1.08E+03 | 6.46E-03 | 1.29E-01 | 6.33E-01 | -1.58E+00 | 8.02E+01 | 1.27E+02 |
| Tep1 | NM_022591 | 1.02E+03 | 1.66E-02 | 1.75E-01 | 6.34E-01 | -1.58E+00 | 7.59E+01 | 1.20E+02 |
| Pick1 | NM_053460 | 3.24E+02 | 2.06E-02 | 1.89E-01 | 6.34E-01 | -1.58E+00 | 2.40E+01 | 3.79E+01 |
| Snrnp48 | NM_001106107 | 1.79E+02 | 7.64E-03 | 1.32E-01 | 6.34E-01 | -1.58E+00 | 1.33E+01 | 2.09E+01 |
| Zmym1 | NM_001107983 | 2.95E+02 | 2.04E-03 | 9.84E-02 | 6.35E-01 | -1.58E+00 | 2.19E+01 | 3.45E+01 |
| Ikbke | NM_001108854 | 3.15E+02 | 9.73E-03 | 1.43E-01 | 6.35E-01 | -1.58E+00 | 2.34E+01 | 3.69E+01 |
| Hspbap1 | NM_134419 | 1.63E+02 | 9.93E-03 | 1.43E-01 | 6.35E-01 | -1.58E+00 | 1.21E+01 | 1.91E+01 |
| Ythdc1 | NM_133423 | 1.10E+03 | 2.80E-03 | 1.08E-01 | 6.36E-01 | -1.57E+00 | 8.18E+01 | 1.29E+02 |
| Gmeb1 | NM_001109268 | 7.00E+01 | 1.58E-02 | 1.71E-01 | 6.36E-01 | -1.57E+00 | 5.21E+00 | 8.20E+00 |
| Kat2a | NM_001107050 | 8.08E+02 | 3.10E-03 | 1.10E-01 | 6.36E-01 | -1.57E+00 | 6.02E+01 | 9.45E+01 |
| Wdr45 | NM_001013958 | 4.66E+02 | 1.44E-03 | 8.33E-02 | 6.37E-01 | -1.57E+00 | 3.47E+01 | 5.45E+01 |
| Pdia5 | NM_001014125 | 7.47E+01 | 5.19E-03 | 1.24E-01 | 6.38E-01 | -1.57E+00 | 5.57E+00 | 8.74E+00 |
| Fnbp4 | NM_001013159 | 1.25E+03 | 1.30E-02 | 1.57E-01 | 6.38E-01 | -1.57E+00 | 9.34E+01 | 1.46E+02 |
| Cryzl1 | NM_001013044 | 1.80E+02 | 3.36E-03 | 1.12E-01 | 6.41E-01 | -1.56E+00 | 1.35E+01 | 2.10E+01 |
| Slc35d2 | NM_001106098 | 1.36E+02 | 1.01E-02 | 1.43E-01 | 6.41E-01 | -1.56E+00 | 1.02E+01 | 1.59E+01 |
| Mdm1 | NM_001017459 | 1.65E+02 | 1.26E-02 | 1.55E-01 | 6.44E-01 | -1.55E+00 | 1.24E+01 | 1.92E+01 |
| Prpf40b | NM_001134583 | 6.14E+02 | 2.73E-03 | 1.07E-01 | 6.44E-01 | -1.55E+00 | 4.61E+01 | 7.16E+01 |
| Hbs1l | NM_001011934 | 8.56E+02 | 4.74E-03 | 1.21E-01 | 6.44E-01 | -1.55E+00 | 6.43E+01 | 9.98E+01 |
| Sp110 | NM_001034137 | 4.21E+02 | 3.79E-03 | 1.17E-01 | 6.45E-01 | -1.55E+00 | 3.16E+01 | 4.91E+01 |
| Lime1 | NM_001108614 | 1.33E+02 | 1.14E-02 | 1.49E-01 | 6.45E-01 | -1.55E+00 | 1.00E+01 | 1.55E+01 |
| Ankrd10 | NM_001271219 | 1.05E+03 | 1.03E-02 | 1.44E-01 | 6.47E-01 | -1.55E+00 | 7.93E+01 | 1.23E+02 |
| Upf3b | NM_001135873 | 4.40E+02 | 2.22E-03 | 9.95E-02 | 6.47E-01 | -1.54E+00 | 3.31E+01 | 5.12E+01 |
| Zfp142 | NM_001108225 | 7.18E+02 | 1.72E-02 | 1.77E-01 | 6.50E-01 | -1.54E+00 | 5.43E+01 | 8.35E+01 |
| Cep83 | NM_001014266 | 3.41E+02 | 2.49E-03 | 1.03E-01 | 6.50E-01 | -1.54E+00 | 2.58E+01 | 3.97E+01 |
| Ncln | NM_001014082 | 1.63E+03 | 7.64E-03 | 1.32E-01 | 6.51E-01 | -1.54E+00 | 1.24E+02 | 1.90E+02 |
| Trip10 | NM_053920 | 9.27E+02 | 1.97E-02 | 1.86E-01 | 6.51E-01 | -1.54E+00 | 7.02E+01 | 1.08E+02 |
| Naxd | NM_001108402 | 4.64E+02 | 1.05E-02 | 1.44E-01 | 6.51E-01 | -1.53E+00 | 3.51E+01 | 5.39E+01 |
| Ahsa2 | NM_001107241 | 1.06E+03 | 3.74E-03 | 1.17E-01 | 6.53E-01 | -1.53E+00 | 8.01E+01 | 1.23E+02 |
| Rab24 | NM_001015023 | 9.38E+02 | 1.10E-02 | 1.47E-01 | 6.53E-01 | -1.53E+00 | 7.12E+01 | 1.09E+02 |
| Supt5h | NM_001107497 | 1.39E+03 | 8.08E-03 | 1.33E-01 | 6.54E-01 | -1.53E+00 | 1.05E+02 | 1.61E+02 |
| Fbxw9 | NM_001081634 | 1.87E+02 | 7.72E-03 | 1.32E-01 | 6.54E-01 | -1.53E+00 | 1.42E+01 | 2.17E+01 |
| RGD1560108 | NM_001109161 | 6.42E+02 | 5.02E-03 | 1.24E-01 | 6.54E-01 | -1.53E+00 | 4.87E+01 | 7.45E+01 |
| Stk38 | NM_001015025 | 1.16E+03 | 8.61E-03 | 1.35E-01 | 6.55E-01 | -1.53E+00 | 8.81E+01 | 1.35E+02 |
| Amdhd2 | NM_001024990 | 2.44E+02 | 1.01E-02 | 1.43E-01 | 6.57E-01 | -1.52E+00 | 1.86E+01 | 2.83E+01 |
| Setd4 | NM_001113747 | 2.43E+02 | 1.31E-02 | 1.57E-01 | 6.59E-01 | -1.52E+00 | 1.86E+01 | 2.82E+01 |
| Gdi1 | NM_017088 | 2.31E+03 | 7.51E-03 | 1.32E-01 | 6.59E-01 | -1.52E+00 | 1.77E+02 | 2.68E+02 |
| Pced1a | NM_001012348 | 2.35E+02 | 1.11E-02 | 1.47E-01 | 6.59E-01 | -1.52E+00 | 1.79E+01 | 2.72E+01 |
| Chka | NM_017127 | 5.88E+02 | 4.45E-03 | 1.19E-01 | 6.59E-01 | -1.52E+00 | 4.49E+01 | 6.81E+01 |
| Mrgbp | NM_001173739.1 | 2.30E+02 | 1.14E-02 | 1.49E-01 | 6.60E-01 | -1.52E+00 | 1.76E+01 | 2.66E+01 |
| Mrgbp | NM_001173739 | 2.30E+02 | 1.14E-02 | 1.49E-01 | 6.60E-01 | -1.52E+00 | 1.76E+01 | 2.66E+01 |
| Gtpbp6 | NM_001135840 | 1.30E+02 | 1.00E-02 | 1.43E-01 | 6.60E-01 | -1.51E+00 | 9.90E+00 | 1.50E+01 |
| Gtpbp6 | NM_001135840.1 | 1.30E+02 | 1.00E-02 | 1.43E-01 | 6.60E-01 | -1.51E+00 | 9.90E+00 | 1.50E+01 |
| Dtymk | NM_001106925 | 5.77E+02 | 2.98E-03 | 1.10E-01 | 6.62E-01 | -1.51E+00 | 4.42E+01 | 6.67E+01 |
| Irf3 | NM_001006969 | 1.37E+03 | 1.00E-02 | 1.43E-01 | 6.62E-01 | -1.51E+00 | 1.05E+02 | 1.58E+02 |
| Dcaf11 | NM_001009686 | 1.45E+03 | 8.20E-03 | 1.33E-01 | 6.63E-01 | -1.51E+00 | 1.11E+02 | 1.68E+02 |
| Trmt2a | NM_001011895 | 3.73E+02 | 1.15E-02 | 1.49E-01 | 6.64E-01 | -1.51E+00 | 2.86E+01 | 4.30E+01 |
| E4f1 | NM_001185046 | 3.22E+02 | 7.58E-03 | 1.32E-01 | 6.64E-01 | -1.51E+00 | 2.47E+01 | 3.72E+01 |
| RGD1311739 | NM_001025691 | 6.47E+02 | 1.72E-02 | 1.78E-01 | 6.65E-01 | -1.50E+00 | 4.96E+01 | 7.47E+01 |
| Cdk11b | NM_145766 | 8.31E+02 | 7.32E-03 | 1.32E-01 | 6.66E-01 | -1.50E+00 | 6.39E+01 | 9.60E+01 |
| Ddx46 | NM_139098 | 9.28E+02 | 5.93E-03 | 1.26E-01 | 6.66E-01 | -1.50E+00 | 7.13E+01 | 1.07E+02 |
| Trub1 | NM_001012173 | 9.59E+01 | 2.13E-02 | 1.92E-01 | 6.66E-01 | -1.50E+00 | 7.37E+00 | 1.11E+01 |
| Kpnb1 | NM_017063 | 3.17E+02 | 4.89E-03 | 1.23E-01 | 1.50E+00 | 1.50E+00 | 3.96E+01 | 2.64E+01 |
| Eaf1 | NM_001107293 | 2.07E+02 | 3.39E-03 | 1.12E-01 | 1.50E+00 | 1.50E+00 | 2.59E+01 | 1.73E+01 |
| Gak | NM_031030 | 4.00E+02 | 1.24E-03 | 8.06E-02 | 1.50E+00 | 1.50E+00 | 5.01E+01 | 3.34E+01 |
| Unc119 | NM_017188 | 1.16E+02 | 2.14E-02 | 1.92E-01 | 1.50E+00 | 1.50E+00 | 1.46E+01 | 9.68E+00 |
| Rab6a | NM_053366 | 9.80E+02 | 8.78E-03 | 1.36E-01 | 1.51E+00 | 1.51E+00 | 1.23E+02 | 8.15E+01 |
| Hmg1l1 | NM_001109373 | 2.20E+02 | 1.80E-02 | 1.81E-01 | 1.51E+00 | 1.51E+00 | 2.76E+01 | 1.83E+01 |
| Yif1a | NM_172017 | 1.58E+02 | 7.31E-03 | 1.32E-01 | 1.51E+00 | 1.51E+00 | 1.98E+01 | 1.31E+01 |
| Psap | NM_013013 | 3.56E+03 | 1.80E-02 | 1.81E-01 | 1.51E+00 | 1.51E+00 | 4.46E+02 | 2.96E+02 |
| Ptprs | NM_019140 | 4.66E+02 | 2.07E-02 | 1.90E-01 | 1.51E+00 | 1.51E+00 | 5.84E+01 | 3.87E+01 |
| Tmem209 | NM_001014055 | 5.46E+01 | 1.91E-02 | 1.85E-01 | 1.51E+00 | 1.51E+00 | 6.85E+00 | 4.54E+00 |
| Pdia6 | NM_001004442 | 2.09E+03 | 7.54E-03 | 1.32E-01 | 1.51E+00 | 1.51E+00 | 2.63E+02 | 1.74E+02 |
| Lrp6 | NM_001107892 | 1.69E+02 | 1.07E-02 | 1.45E-01 | 1.51E+00 | 1.51E+00 | 2.12E+01 | 1.40E+01 |
| Dync1li1 | NM_145772 | 7.19E+01 | 1.49E-02 | 1.67E-01 | 1.51E+00 | 1.51E+00 | 9.02E+00 | 5.97E+00 |
| RGD1307752 | NM_001013922 | 4.92E+02 | 4.48E-03 | 1.19E-01 | 1.51E+00 | 1.51E+00 | 6.17E+01 | 4.08E+01 |
| Naa60 | NM_001014226 | 2.90E+02 | 6.35E-03 | 1.28E-01 | 1.51E+00 | 1.51E+00 | 3.64E+01 | 2.41E+01 |
| Ankrd50 | NM_001191606 | 1.05E+02 | 1.45E-02 | 1.65E-01 | 1.51E+00 | 1.51E+00 | 1.31E+01 | 8.69E+00 |
| Lrp5 | NM_001106321 | 7.37E+02 | 3.98E-03 | 1.17E-01 | 1.51E+00 | 1.51E+00 | 9.25E+01 | 6.12E+01 |
| Fem1a | NM_001025706 | 9.98E+01 | 8.10E-03 | 1.33E-01 | 1.51E+00 | 1.51E+00 | 1.25E+01 | 8.28E+00 |
| LOC314140 | NM_001009694 | 6.93E+01 | 1.94E-02 | 1.85E-01 | 1.51E+00 | 1.51E+00 | 8.70E+00 | 5.75E+00 |
| Champ1 | NM_001107329 | 2.42E+02 | 2.21E-02 | 1.94E-01 | 1.51E+00 | 1.51E+00 | 3.04E+01 | 2.01E+01 |
| Tgfb1 | NM_021578 | 2.01E+02 | 1.63E-02 | 1.74E-01 | 1.52E+00 | 1.52E+00 | 2.53E+01 | 1.67E+01 |
| Abcb8 | NM_001007796 | 6.66E+01 | 1.01E-02 | 1.43E-01 | 1.52E+00 | 1.52E+00 | 8.37E+00 | 5.52E+00 |
| RGD1305350 | NM_001135781 | 4.14E+02 | 1.75E-02 | 1.80E-01 | 1.52E+00 | 1.52E+00 | 5.21E+01 | 3.43E+01 |
| P4hb | NM_012998 | 5.26E+03 | 2.20E-02 | 1.93E-01 | 1.52E+00 | 1.52E+00 | 6.61E+02 | 4.36E+02 |
| Anp32e | NM_001013200 | 1.92E+02 | 7.19E-03 | 1.32E-01 | 1.52E+00 | 1.52E+00 | 2.41E+01 | 1.59E+01 |
| Ccdc47 | NM_001013974 | 9.01E+02 | 8.61E-03 | 1.35E-01 | 1.52E+00 | 1.52E+00 | 1.13E+02 | 7.46E+01 |
| Pigx | NM_001100651 | 1.62E+02 | 7.04E-03 | 1.32E-01 | 1.52E+00 | 1.52E+00 | 2.03E+01 | 1.34E+01 |
| Natd1 | NM_001170541 | 9.89E+01 | 6.26E-03 | 1.28E-01 | 1.52E+00 | 1.52E+00 | 1.24E+01 | 8.18E+00 |
| Natd1 | NM_001170541.1 | 9.89E+01 | 6.26E-03 | 1.28E-01 | 1.52E+00 | 1.52E+00 | 1.24E+01 | 8.18E+00 |
| Atp6v1a | NM_001108318 | 5.24E+02 | 2.70E-03 | 1.07E-01 | 1.52E+00 | 1.52E+00 | 6.60E+01 | 4.33E+01 |
| Psmb2 | NM_017284 | 4.45E+02 | 8.42E-03 | 1.34E-01 | 1.52E+00 | 1.52E+00 | 5.60E+01 | 3.68E+01 |
| Atp5a1 | NM_023093 | 2.41E+03 | 1.01E-02 | 1.43E-01 | 1.52E+00 | 1.52E+00 | 3.04E+02 | 1.99E+02 |
| Hint3 | NM_001100825 | 1.40E+02 | 7.11E-03 | 1.32E-01 | 1.53E+00 | 1.53E+00 | 1.76E+01 | 1.15E+01 |
| Hmbs | NM_013168 | 2.01E+02 | 8.24E-03 | 1.34E-01 | 1.53E+00 | 1.53E+00 | 2.53E+01 | 1.66E+01 |
| Cd68 | NM_001031638 | 1.35E+02 | 1.22E-02 | 1.53E-01 | 1.53E+00 | 1.53E+00 | 1.71E+01 | 1.12E+01 |
| Mettl2b | NM_001108839 | 3.11E+01 | 1.18E-02 | 1.51E-01 | 1.53E+00 | 1.53E+00 | 3.92E+00 | 2.56E+00 |
| Cpox | NM_001037095 | 1.85E+02 | 9.01E-03 | 1.38E-01 | 1.53E+00 | 1.53E+00 | 2.34E+01 | 1.53E+01 |
| Zcchc11 | NM_001107953 | 1.13E+02 | 1.54E-02 | 1.69E-01 | 1.53E+00 | 1.53E+00 | 1.43E+01 | 9.35E+00 |
| Ubc | NM_017314 | 5.11E+03 | 1.63E-02 | 1.74E-01 | 1.53E+00 | 1.53E+00 | 6.46E+02 | 4.22E+02 |
| Atpaf1 | NM_001107959 | 1.45E+02 | 1.57E-02 | 1.70E-01 | 1.53E+00 | 1.53E+00 | 1.84E+01 | 1.20E+01 |
| Mul1 | NM_001106695 | 2.10E+02 | 5.22E-03 | 1.24E-01 | 1.53E+00 | 1.53E+00 | 2.66E+01 | 1.73E+01 |
| Cdkn2aip | NM_001014000 | 1.07E+02 | 1.15E-02 | 1.49E-01 | 1.54E+00 | 1.54E+00 | 1.36E+01 | 8.84E+00 |
| Lrpap1 | NM_001169113 | 1.92E+02 | 3.80E-03 | 1.17E-01 | 1.54E+00 | 1.54E+00 | 2.43E+01 | 1.58E+01 |
| Itgb5 | NM_147139 | 1.13E+03 | 2.15E-02 | 1.92E-01 | 1.54E+00 | 1.54E+00 | 1.42E+02 | 9.27E+01 |
| Tmem98 | NM_001007672 | 2.59E+02 | 1.80E-02 | 1.81E-01 | 1.54E+00 | 1.54E+00 | 3.28E+01 | 2.13E+01 |
| Tmem30a | NM_001004248 | 5.58E+02 | 1.64E-02 | 1.74E-01 | 1.54E+00 | 1.54E+00 | 7.07E+01 | 4.60E+01 |
| Spryd7 | NM_001009635 | 1.80E+02 | 8.62E-03 | 1.35E-01 | 1.54E+00 | 1.54E+00 | 2.28E+01 | 1.49E+01 |
| Atad1 | NM_001035002 | 2.72E+02 | 9.70E-03 | 1.43E-01 | 1.54E+00 | 1.54E+00 | 3.44E+01 | 2.24E+01 |
| Dad1 | NM_138910 | 6.64E+02 | 1.84E-02 | 1.82E-01 | 1.54E+00 | 1.54E+00 | 8.40E+01 | 5.46E+01 |
| Calu | NM_001033898 | 7.34E+02 | 1.04E-02 | 1.44E-01 | 1.54E+00 | 1.54E+00 | 9.30E+01 | 6.04E+01 |
| Letm1 | NM_001005884 | 8.85E+01 | 1.60E-02 | 1.72E-01 | 1.54E+00 | 1.54E+00 | 1.12E+01 | 7.28E+00 |
| Tm9sf2 | NM_001005554 | 1.39E+03 | 1.68E-02 | 1.75E-01 | 1.54E+00 | 1.54E+00 | 1.76E+02 | 1.14E+02 |
| Rdh13 | NM_001108468 | 4.91E+01 | 1.93E-02 | 1.85E-01 | 1.54E+00 | 1.54E+00 | 6.23E+00 | 4.04E+00 |
| RT1-S3 | NM_001008886 | 1.04E+03 | 7.86E-03 | 1.32E-01 | 1.54E+00 | 1.54E+00 | 1.31E+02 | 8.50E+01 |
| Laptm5 | NM_053538 | 4.16E+02 | 9.36E-03 | 1.41E-01 | 1.54E+00 | 1.54E+00 | 5.28E+01 | 3.42E+01 |
| Pptc7 | NM_001107141 | 1.62E+02 | 3.79E-03 | 1.17E-01 | 1.54E+00 | 1.54E+00 | 2.06E+01 | 1.33E+01 |
| Usp34 | NM_001271196 | 3.57E+02 | 1.34E-02 | 1.59E-01 | 1.54E+00 | 1.54E+00 | 4.53E+01 | 2.93E+01 |
| Hnrnpf | NM_001037287 | 8.65E+02 | 1.93E-02 | 1.85E-01 | 1.55E+00 | 1.55E+00 | 1.10E+02 | 7.10E+01 |
| Tp53i3 | NM_001113775 | 1.19E+02 | 1.67E-03 | 9.01E-02 | 1.55E+00 | 1.55E+00 | 1.51E+01 | 9.79E+00 |
| Atp2b1 | NM_053311 | 9.88E+01 | 1.53E-02 | 1.69E-01 | 1.55E+00 | 1.55E+00 | 1.25E+01 | 8.11E+00 |
| Tlr3 | NM_198791 | 1.00E+02 | 8.51E-03 | 1.34E-01 | 1.55E+00 | 1.55E+00 | 1.27E+01 | 8.23E+00 |
| Pfkl | NM_013190 | 3.77E+02 | 4.52E-03 | 1.19E-01 | 1.55E+00 | 1.55E+00 | 4.79E+01 | 3.09E+01 |
| Os9 | NM_001007265 | 9.56E+02 | 3.98E-03 | 1.17E-01 | 1.55E+00 | 1.55E+00 | 1.21E+02 | 7.85E+01 |
| Sh2d4a | NM_001012048 | 1.59E+02 | 1.06E-02 | 1.45E-01 | 1.55E+00 | 1.55E+00 | 2.02E+01 | 1.31E+01 |
| Gtpbp4 | NM_053689 | 1.17E+02 | 7.39E-03 | 1.32E-01 | 1.55E+00 | 1.55E+00 | 1.48E+01 | 9.57E+00 |
| Actr3 | NM_031068 | 9.01E+02 | 1.20E-02 | 1.52E-01 | 1.55E+00 | 1.55E+00 | 1.14E+02 | 7.39E+01 |
| Rnf139 | NM_001127545 | 1.87E+02 | 8.77E-03 | 1.36E-01 | 1.55E+00 | 1.55E+00 | 2.38E+01 | 1.54E+01 |
| Cyb561a3 | NM_001014164 | 1.53E+02 | 1.03E-02 | 1.44E-01 | 1.55E+00 | 1.55E+00 | 1.94E+01 | 1.25E+01 |
| Pdia3 | NM_017319 | 3.69E+03 | 1.67E-02 | 1.75E-01 | 1.55E+00 | 1.55E+00 | 4.69E+02 | 3.03E+02 |
| Aph1a | NM_001014255 | 6.14E+02 | 1.85E-03 | 9.56E-02 | 1.55E+00 | 1.55E+00 | 7.81E+01 | 5.03E+01 |
| Plekhb1 | NM_172033 | 6.94E+02 | 2.15E-02 | 1.92E-01 | 1.55E+00 | 1.55E+00 | 8.82E+01 | 5.68E+01 |
| Ctnnd1 | NM_001107740 | 8.77E+02 | 4.35E-03 | 1.19E-01 | 1.55E+00 | 1.55E+00 | 1.12E+02 | 7.17E+01 |
| Tmem57 | NM_001025699 | 2.54E+02 | 5.23E-03 | 1.24E-01 | 1.56E+00 | 1.56E+00 | 3.24E+01 | 2.08E+01 |
| Orai3 | NM_001014024 | 1.69E+02 | 5.50E-03 | 1.25E-01 | 1.56E+00 | 1.56E+00 | 2.15E+01 | 1.38E+01 |
| Tcf20 | NM_001130574 | 1.70E+02 | 2.11E-02 | 1.91E-01 | 1.56E+00 | 1.56E+00 | 2.17E+01 | 1.39E+01 |
| Ddx21 | NM_001037201 | 5.27E+02 | 1.88E-02 | 1.84E-01 | 1.56E+00 | 1.56E+00 | 6.72E+01 | 4.31E+01 |
| Abhd13 | NM_001271072 | 2.49E+02 | 1.22E-02 | 1.53E-01 | 1.56E+00 | 1.56E+00 | 3.17E+01 | 2.04E+01 |
| Coro1a | NM_130411 | 2.52E+02 | 7.23E-03 | 1.32E-01 | 1.56E+00 | 1.56E+00 | 3.21E+01 | 2.05E+01 |
| Tor1a | NM_153303 | 1.85E+02 | 2.19E-03 | 9.95E-02 | 1.56E+00 | 1.56E+00 | 2.36E+01 | 1.51E+01 |
| Dnajb11 | NM_001015021 | 4.28E+02 | 5.35E-03 | 1.24E-01 | 1.56E+00 | 1.56E+00 | 5.45E+01 | 3.49E+01 |
| Tpp1 | NM_031357 | 4.58E+02 | 6.81E-04 | 6.85E-02 | 1.56E+00 | 1.56E+00 | 5.84E+01 | 3.74E+01 |
| Tomm40 | NM_212520 | 1.05E+02 | 1.75E-02 | 1.80E-01 | 1.56E+00 | 1.56E+00 | 1.34E+01 | 8.58E+00 |
| Smim7 | NM_001135252 | 3.60E+02 | 4.97E-03 | 1.23E-01 | 1.57E+00 | 1.57E+00 | 4.59E+01 | 2.93E+01 |
| Pttg1ip | NM_001013238 | 1.83E+03 | 1.45E-02 | 1.65E-01 | 1.57E+00 | 1.57E+00 | 2.34E+02 | 1.49E+02 |
| Glg1 | NM_017211 | 1.72E+02 | 1.91E-02 | 1.85E-01 | 1.57E+00 | 1.57E+00 | 2.20E+01 | 1.40E+01 |
| Prpf8 | NM_001191590 | 8.26E+02 | 4.57E-03 | 1.20E-01 | 1.57E+00 | 1.57E+00 | 1.05E+02 | 6.73E+01 |
| Atpif1 | NM_012915 | 5.00E+02 | 9.78E-03 | 1.43E-01 | 1.57E+00 | 1.57E+00 | 6.38E+01 | 4.07E+01 |
| Copb1 | NM_080781 | 6.53E+02 | 6.70E-03 | 1.29E-01 | 1.57E+00 | 1.57E+00 | 8.34E+01 | 5.32E+01 |
| Chic2 | NM_001105736 | 1.16E+02 | 8.50E-03 | 1.34E-01 | 1.57E+00 | 1.57E+00 | 1.49E+01 | 9.48E+00 |
| Iars | NM_001100572 | 2.32E+02 | 1.72E-03 | 9.17E-02 | 1.57E+00 | 1.57E+00 | 2.97E+01 | 1.89E+01 |
| Nt5c | NM_001271189 | 5.71E+01 | 1.61E-02 | 1.72E-01 | 1.57E+00 | 1.57E+00 | 7.31E+00 | 4.65E+00 |
| Snrnp70 | NM_001108483 | 2.67E+02 | 2.16E-02 | 1.92E-01 | 1.57E+00 | 1.57E+00 | 3.42E+01 | 2.18E+01 |
| Eprs | NM_001024238 | 6.33E+02 | 8.19E-03 | 1.33E-01 | 1.57E+00 | 1.57E+00 | 8.09E+01 | 5.15E+01 |
| Tmem127 | NM_001100978 | 6.99E+02 | 2.02E-02 | 1.87E-01 | 1.57E+00 | 1.57E+00 | 8.95E+01 | 5.68E+01 |
| Lrp10 | NM_001037777 | 9.60E+02 | 1.26E-02 | 1.55E-01 | 1.58E+00 | 1.58E+00 | 1.23E+02 | 7.80E+01 |
| Sdf4 | NM_130412 | 6.98E+02 | 4.21E-03 | 1.19E-01 | 1.58E+00 | 1.58E+00 | 8.94E+01 | 5.67E+01 |
| C2 | NM_172222 | 1.11E+03 | 2.61E-03 | 1.06E-01 | 1.58E+00 | 1.58E+00 | 1.42E+02 | 9.01E+01 |
| Gpr155 | NM_001107811 | 1.62E+02 | 7.03E-04 | 6.85E-02 | 1.58E+00 | 1.58E+00 | 2.07E+01 | 1.31E+01 |
| Pigt | NM_001106540 | 5.62E+02 | 3.62E-03 | 1.17E-01 | 1.58E+00 | 1.58E+00 | 7.21E+01 | 4.56E+01 |
| Ncstn | NM_174864 | 6.66E+02 | 6.86E-03 | 1.31E-01 | 1.58E+00 | 1.58E+00 | 8.54E+01 | 5.40E+01 |
| Ssr4 | NM_017199 | 5.58E+02 | 2.05E-02 | 1.89E-01 | 1.58E+00 | 1.58E+00 | 7.15E+01 | 4.53E+01 |
| Tpk1 | NM_001134994 | 7.83E+01 | 9.47E-03 | 1.42E-01 | 1.58E+00 | 1.58E+00 | 1.00E+01 | 6.35E+00 |
| Rspry1 | NM_001100945 | 3.47E+01 | 2.20E-02 | 1.93E-01 | 1.58E+00 | 1.58E+00 | 4.45E+00 | 2.82E+00 |
| Smad7 | NM_030858 | 1.86E+02 | 7.37E-03 | 1.32E-01 | 1.58E+00 | 1.58E+00 | 2.38E+01 | 1.51E+01 |
| Ndufs1 | NM_001005550 | 3.19E+02 | 6.55E-03 | 1.29E-01 | 1.58E+00 | 1.58E+00 | 4.09E+01 | 2.59E+01 |
| Tmed2 | NM_031722 | 2.27E+03 | 1.04E-02 | 1.44E-01 | 1.59E+00 | 1.59E+00 | 2.92E+02 | 1.84E+02 |
| Trappc9 | NM_001034156 | 1.36E+02 | 5.92E-03 | 1.26E-01 | 1.59E+00 | 1.59E+00 | 1.74E+01 | 1.10E+01 |
| Ifitm2 | NM_030833 | 2.06E+03 | 1.49E-02 | 1.67E-01 | 1.59E+00 | 1.59E+00 | 2.64E+02 | 1.67E+02 |
| Rab10 | NM_017359 | 7.22E+02 | 2.91E-03 | 1.10E-01 | 1.59E+00 | 1.59E+00 | 9.28E+01 | 5.85E+01 |
| Myh9 | NM_001305877 | 5.11E+02 | 7.77E-03 | 1.32E-01 | 1.59E+00 | 1.59E+00 | 6.57E+01 | 4.14E+01 |
| Abcb10 | NM_001012166 | 9.87E+01 | 1.37E-02 | 1.61E-01 | 1.59E+00 | 1.59E+00 | 1.27E+01 | 7.99E+00 |
| Canx | NM_172008 | 3.68E+03 | 7.08E-03 | 1.32E-01 | 1.59E+00 | 1.59E+00 | 4.74E+02 | 2.98E+02 |
| Jkamp | NM_001106738 | 6.80E+01 | 1.88E-02 | 1.84E-01 | 1.59E+00 | 1.59E+00 | 8.75E+00 | 5.50E+00 |
| Farsb | NM_001004252 | 1.76E+02 | 3.82E-03 | 1.17E-01 | 1.59E+00 | 1.59E+00 | 2.27E+01 | 1.42E+01 |
| Sec11a | NM_031723 | 5.07E+02 | 1.54E-02 | 1.69E-01 | 1.59E+00 | 1.59E+00 | 6.52E+01 | 4.10E+01 |
| Ptpn6 | NM_053908 | 1.67E+02 | 6.62E-03 | 1.29E-01 | 1.59E+00 | 1.59E+00 | 2.14E+01 | 1.35E+01 |
| Tmem185a | NM_001135712 | 1.73E+02 | 1.31E-02 | 1.57E-01 | 1.59E+00 | 1.59E+00 | 2.22E+01 | 1.40E+01 |
| Calm2 | NM_017326.1 | 5.25E+02 | 2.28E-02 | 1.97E-01 | 1.60E+00 | 1.60E+00 | 6.77E+01 | 4.24E+01 |
| Top2b | NM_001100858 | 4.48E+02 | 1.93E-02 | 1.85E-01 | 1.60E+00 | 1.60E+00 | 5.77E+01 | 3.62E+01 |
| Rnf13 | NM_001109444 | 6.11E+02 | 1.49E-02 | 1.67E-01 | 1.60E+00 | 1.60E+00 | 7.88E+01 | 4.94E+01 |
| Clcn6 | NM_001106479 | 5.20E+01 | 1.38E-02 | 1.62E-01 | 1.60E+00 | 1.60E+00 | 6.70E+00 | 4.19E+00 |
| Irf2 | NM_001047086 | 8.06E+01 | 9.44E-03 | 1.42E-01 | 1.60E+00 | 1.60E+00 | 1.04E+01 | 6.50E+00 |
| Hdgf | NM_053707 | 4.26E+02 | 4.03E-03 | 1.18E-01 | 1.60E+00 | 1.60E+00 | 5.50E+01 | 3.44E+01 |
| Paics | NM_080910 | 6.34E+02 | 1.13E-03 | 8.00E-02 | 1.61E+00 | 1.61E+00 | 8.19E+01 | 5.10E+01 |
| Sf3b4 | NM_001011951 | 2.28E+02 | 2.34E-02 | 1.99E-01 | 1.61E+00 | 1.61E+00 | 2.95E+01 | 1.83E+01 |
| Slc2a9 | NM_001191551 | 1.29E+02 | 1.63E-03 | 8.83E-02 | 1.61E+00 | 1.61E+00 | 1.67E+01 | 1.04E+01 |
| Adgrl2 | NM_001302209 | 5.05E+01 | 1.04E-02 | 1.44E-01 | 1.61E+00 | 1.61E+00 | 6.54E+00 | 4.07E+00 |
| Mboat7l1 | NM_001313940.1 | 7.54E+01 | 2.02E-02 | 1.87E-01 | 1.61E+00 | 1.61E+00 | 9.77E+00 | 6.06E+00 |
| Mboat7 | NM_001134978.1 | 7.54E+01 | 2.02E-02 | 1.87E-01 | 1.61E+00 | 1.61E+00 | 9.77E+00 | 6.06E+00 |
| Mboat7l1 | NM_001313940 | 7.54E+01 | 2.02E-02 | 1.87E-01 | 1.61E+00 | 1.61E+00 | 9.77E+00 | 6.06E+00 |
| Mboat7 | NM_001134978 | 7.54E+01 | 2.02E-02 | 1.87E-01 | 1.61E+00 | 1.61E+00 | 9.77E+00 | 6.06E+00 |
| Clcn3 | NM_053363 | 7.02E+01 | 1.27E-02 | 1.55E-01 | 1.61E+00 | 1.61E+00 | 9.10E+00 | 5.64E+00 |
| Hnrnph2 | NM_001014019 | 2.53E+02 | 4.28E-03 | 1.19E-01 | 1.61E+00 | 1.61E+00 | 3.27E+01 | 2.03E+01 |
| Itfg1 | NM_133557 | 6.53E+02 | 3.34E-03 | 1.12E-01 | 1.61E+00 | 1.61E+00 | 8.46E+01 | 5.24E+01 |
| Park7 | NM_001277253 | 8.55E+01 | 8.41E-03 | 1.34E-01 | 1.61E+00 | 1.61E+00 | 1.11E+01 | 6.86E+00 |
| Eno1 | NM_012554 | 1.51E+03 | 3.70E-03 | 1.17E-01 | 1.61E+00 | 1.61E+00 | 1.95E+02 | 1.21E+02 |
| Ssr3 | NM_031120 | 1.12E+03 | 3.19E-03 | 1.10E-01 | 1.62E+00 | 1.62E+00 | 1.45E+02 | 8.99E+01 |
| Tmem231 | NM_001271031 | 5.94E+01 | 1.53E-02 | 1.69E-01 | 1.62E+00 | 1.62E+00 | 7.70E+00 | 4.77E+00 |
| Azin1 | NM_022585 | 8.51E+01 | 9.55E-03 | 1.42E-01 | 1.62E+00 | 1.62E+00 | 1.10E+01 | 6.83E+00 |
| B3galt4 | NM_133553 | 5.25E+01 | 1.05E-02 | 1.44E-01 | 1.62E+00 | 1.62E+00 | 6.81E+00 | 4.21E+00 |
| Adck2 | NM_001107855 | 9.02E+01 | 4.27E-03 | 1.19E-01 | 1.62E+00 | 1.62E+00 | 1.17E+01 | 7.22E+00 |
| Lman2 | NM_001115024 | 6.22E+02 | 1.23E-02 | 1.54E-01 | 1.62E+00 | 1.62E+00 | 8.08E+01 | 4.98E+01 |
| Myh9l1 | NM_013194.1 | 1.41E+03 | 1.28E-02 | 1.55E-01 | 1.62E+00 | 1.62E+00 | 1.84E+02 | 1.13E+02 |
| Myh9l1 | NM_013194 | 1.41E+03 | 1.28E-02 | 1.55E-01 | 1.62E+00 | 1.62E+00 | 1.84E+02 | 1.13E+02 |
| Ndfip2 | NM_001108390 | 2.94E+02 | 7.82E-03 | 1.32E-01 | 1.63E+00 | 1.63E+00 | 3.83E+01 | 2.35E+01 |
| Gemin5 | NM_001172089 | 8.42E+01 | 1.78E-02 | 1.81E-01 | 1.63E+00 | 1.63E+00 | 1.10E+01 | 6.73E+00 |
| Arl8b | NM_001024332 | 9.74E+02 | 1.87E-02 | 1.84E-01 | 1.63E+00 | 1.63E+00 | 1.27E+02 | 7.78E+01 |
| Ssr1 | NM_001008891 | 8.02E+02 | 8.91E-03 | 1.38E-01 | 1.63E+00 | 1.63E+00 | 1.05E+02 | 6.40E+01 |
| Sdc3 | NM_053893 | 5.27E+02 | 1.90E-02 | 1.85E-01 | 1.64E+00 | 1.64E+00 | 6.87E+01 | 4.20E+01 |
| Nf1 | NM_012609 | 9.52E+01 | 1.90E-02 | 1.85E-01 | 1.64E+00 | 1.64E+00 | 1.24E+01 | 7.59E+00 |
| Bcap29 | NM_001006980 | 1.97E+02 | 1.22E-02 | 1.53E-01 | 1.64E+00 | 1.64E+00 | 2.57E+01 | 1.57E+01 |
| Acsl3 | NM_057107 | 1.64E+02 | 6.12E-03 | 1.27E-01 | 1.64E+00 | 1.64E+00 | 2.14E+01 | 1.30E+01 |
| Ptprj | NM_017269 | 3.05E+02 | 1.43E-03 | 8.33E-02 | 1.64E+00 | 1.64E+00 | 3.98E+01 | 2.43E+01 |
| Eef1e1 | NM_001106106 | 1.67E+02 | 1.87E-02 | 1.84E-01 | 1.64E+00 | 1.64E+00 | 2.18E+01 | 1.33E+01 |
| Sumf1 | NM_001108639 | 3.46E+02 | 1.38E-02 | 1.62E-01 | 1.64E+00 | 1.64E+00 | 4.52E+01 | 2.76E+01 |
| Slc39a1 | NM_001134577 | 9.53E+02 | 1.81E-02 | 1.82E-01 | 1.64E+00 | 1.64E+00 | 1.25E+02 | 7.59E+01 |
| Gcs1 | NM_031749 | 1.63E+02 | 5.90E-03 | 1.26E-01 | 1.64E+00 | 1.64E+00 | 2.13E+01 | 1.30E+01 |
| Gcs1 | NM_031749.1 | 1.63E+02 | 5.90E-03 | 1.26E-01 | 1.64E+00 | 1.64E+00 | 2.13E+01 | 1.30E+01 |
| Aars2 | NM_001106891 | 4.36E+01 | 3.35E-03 | 1.12E-01 | 1.64E+00 | 1.64E+00 | 5.69E+00 | 3.47E+00 |
| Emc10 | NM_001004221 | 6.25E+02 | 8.96E-03 | 1.38E-01 | 1.64E+00 | 1.64E+00 | 8.16E+01 | 4.97E+01 |
| Apc | NM_012499 | 1.57E+02 | 8.31E-03 | 1.34E-01 | 1.64E+00 | 1.64E+00 | 2.05E+01 | 1.25E+01 |
| Ryk | NM_080402 | 1.89E+02 | 9.02E-03 | 1.38E-01 | 1.65E+00 | 1.65E+00 | 2.47E+01 | 1.50E+01 |
| Stard7 | NM_001106503 | 1.01E+02 | 2.29E-03 | 9.95E-02 | 1.65E+00 | 1.65E+00 | 1.33E+01 | 8.06E+00 |
| Zbtb1 | NM_001004444 | 1.31E+02 | 2.32E-02 | 1.98E-01 | 1.65E+00 | 1.65E+00 | 1.72E+01 | 1.05E+01 |
| Ifi30 | NM_001030026 | 4.84E+02 | 1.57E-02 | 1.70E-01 | 1.65E+00 | 1.65E+00 | 6.34E+01 | 3.85E+01 |
| Tmed3 | NM_001004249 | 5.54E+02 | 8.27E-03 | 1.34E-01 | 1.65E+00 | 1.65E+00 | 7.25E+01 | 4.40E+01 |
| Dchs1 | NM_001107544 | 2.38E+02 | 1.82E-02 | 1.82E-01 | 1.65E+00 | 1.65E+00 | 3.11E+01 | 1.89E+01 |
| Cldnd1 | NM_001006955 | 5.21E+02 | 3.42E-03 | 1.12E-01 | 1.65E+00 | 1.65E+00 | 6.82E+01 | 4.13E+01 |
| Adnp2 | NM_001127373 | 8.70E+01 | 1.50E-03 | 8.43E-02 | 1.65E+00 | 1.65E+00 | 1.14E+01 | 6.90E+00 |
| Ccnl2 | NM_001013094 | 1.19E+02 | 4.87E-03 | 1.23E-01 | 1.65E+00 | 1.65E+00 | 1.56E+01 | 9.42E+00 |
| Sec61a1 | NM_199256 | 1.14E+03 | 6.66E-03 | 1.29E-01 | 1.65E+00 | 1.65E+00 | 1.50E+02 | 9.08E+01 |
| Saraf | NM_001004213 | 7.55E+02 | 1.40E-02 | 1.63E-01 | 1.65E+00 | 1.65E+00 | 9.90E+01 | 5.98E+01 |
| Manf | NM_001108183 | 1.31E+02 | 2.25E-02 | 1.96E-01 | 1.66E+00 | 1.66E+00 | 1.72E+01 | 1.04E+01 |
| Bfar | NM_001013125 | 1.94E+02 | 5.17E-03 | 1.24E-01 | 1.66E+00 | 1.66E+00 | 2.54E+01 | 1.54E+01 |
| Nomo1 | NM_001108484 | 3.28E+02 | 6.18E-04 | 6.62E-02 | 1.66E+00 | 1.66E+00 | 4.31E+01 | 2.60E+01 |
| Kdelc2 | NM_001025123 | 5.31E+02 | 1.92E-02 | 1.85E-01 | 1.66E+00 | 1.66E+00 | 6.98E+01 | 4.20E+01 |
| Hax1 | NM_181627 | 1.06E+02 | 1.01E-03 | 7.90E-02 | 1.66E+00 | 1.66E+00 | 1.39E+01 | 8.38E+00 |
| Ifngr2 | NM_001108313 | 2.48E+02 | 3.52E-04 | 6.47E-02 | 1.67E+00 | 1.67E+00 | 3.27E+01 | 1.96E+01 |
| Il13ra1 | NM_145789 | 5.16E+02 | 1.95E-02 | 1.86E-01 | 1.67E+00 | 1.67E+00 | 6.79E+01 | 4.07E+01 |
| RT1-CE4 | NM_001008842 | 2.21E+02 | 5.01E-03 | 1.24E-01 | 1.67E+00 | 1.67E+00 | 2.90E+01 | 1.74E+01 |
| Cd81 | NM_013087 | 2.90E+03 | 5.14E-03 | 1.24E-01 | 1.67E+00 | 1.67E+00 | 3.82E+02 | 2.28E+02 |
| Clip1 | NM_031745 | 9.53E+01 | 2.59E-03 | 1.06E-01 | 1.67E+00 | 1.67E+00 | 1.26E+01 | 7.51E+00 |
| Yif1b | NM_001014810.1 | 7.55E+01 | 5.68E-03 | 1.26E-01 | 1.68E+00 | 1.68E+00 | 9.96E+00 | 5.94E+00 |
| Yif1b | NM_001014810 | 7.55E+01 | 5.68E-03 | 1.26E-01 | 1.68E+00 | 1.68E+00 | 9.96E+00 | 5.94E+00 |
| Tsfm | NM_001276476 | 8.07E+01 | 1.18E-03 | 8.06E-02 | 1.68E+00 | 1.68E+00 | 1.06E+01 | 6.35E+00 |
| Slc35f5 | NM_001105950 | 1.90E+02 | 2.54E-03 | 1.05E-01 | 1.68E+00 | 1.68E+00 | 2.50E+01 | 1.49E+01 |
| Tmem186 | NM_001025756 | 1.14E+02 | 1.05E-02 | 1.44E-01 | 1.68E+00 | 1.68E+00 | 1.50E+01 | 8.96E+00 |
| Mri1 | NM_001010947 | 7.62E+01 | 3.67E-03 | 1.17E-01 | 1.68E+00 | 1.68E+00 | 1.01E+01 | 5.99E+00 |
| Mgat2 | NM_053604 | 3.01E+02 | 1.21E-03 | 8.06E-02 | 1.68E+00 | 1.68E+00 | 3.97E+01 | 2.36E+01 |
| Kdelr2 | NM_001013122 | 6.29E+02 | 5.91E-03 | 1.26E-01 | 1.68E+00 | 1.68E+00 | 8.32E+01 | 4.95E+01 |
| Mmp19 | NM_001107159 | 2.20E+02 | 1.48E-02 | 1.67E-01 | 1.68E+00 | 1.68E+00 | 2.91E+01 | 1.73E+01 |
| Zmynd19 | NM_198770 | 1.27E+02 | 1.83E-02 | 1.82E-01 | 1.68E+00 | 1.68E+00 | 1.68E+01 | 1.00E+01 |
| Tmem59 | NM_001139465 | 1.84E+03 | 3.60E-03 | 1.17E-01 | 1.68E+00 | 1.68E+00 | 2.43E+02 | 1.44E+02 |
| Emilin1 | NM_001106710 | 2.86E+02 | 1.62E-02 | 1.73E-01 | 1.69E+00 | 1.69E+00 | 3.79E+01 | 2.25E+01 |
| Lmtk2 | NM_001137641 | 2.21E+02 | 1.59E-03 | 8.75E-02 | 1.69E+00 | 1.69E+00 | 2.93E+01 | 1.73E+01 |
| Peli1 | NM_001100565 | 9.52E+01 | 6.53E-03 | 1.29E-01 | 1.69E+00 | 1.69E+00 | 1.26E+01 | 7.46E+00 |
| Cd82 | NM_031797 | 9.79E+02 | 2.09E-02 | 1.91E-01 | 1.69E+00 | 1.69E+00 | 1.30E+02 | 7.68E+01 |
| Extl3 | NM_020097 | 6.55E+01 | 1.77E-02 | 1.81E-01 | 1.69E+00 | 1.69E+00 | 8.67E+00 | 5.13E+00 |
| Myo1e | NM_173101 | 2.02E+02 | 1.20E-03 | 8.06E-02 | 1.69E+00 | 1.69E+00 | 2.68E+01 | 1.58E+01 |
| Pfdn6 | NM_212506 | 1.23E+02 | 8.14E-04 | 7.42E-02 | 1.69E+00 | 1.69E+00 | 1.63E+01 | 9.60E+00 |
| Sptlc2 | NM_001037097 | 2.04E+02 | 2.27E-02 | 1.97E-01 | 1.70E+00 | 1.70E+00 | 2.71E+01 | 1.60E+01 |
| Lcp1 | NM_001012044 | 6.58E+02 | 1.26E-02 | 1.55E-01 | 1.70E+00 | 1.70E+00 | 8.74E+01 | 5.14E+01 |
| Yipf1 | NM_199383 | 1.29E+02 | 3.77E-03 | 1.17E-01 | 1.70E+00 | 1.70E+00 | 1.72E+01 | 1.01E+01 |
| RGD1311164 | NM_001106623 | 6.84E+01 | 1.47E-02 | 1.66E-01 | 1.70E+00 | 1.70E+00 | 9.09E+00 | 5.34E+00 |
| Srprb | NM_001013252 | 1.07E+02 | 1.08E-02 | 1.45E-01 | 1.70E+00 | 1.70E+00 | 1.43E+01 | 8.38E+00 |
| Cobl | NM_001107236 | 1.81E+02 | 8.30E-03 | 1.34E-01 | 1.70E+00 | 1.70E+00 | 2.40E+01 | 1.41E+01 |
| Ctss | NM_017320 | 4.41E+02 | 6.32E-03 | 1.28E-01 | 1.70E+00 | 1.70E+00 | 5.86E+01 | 3.44E+01 |
| Nectin2 | NM_001012064 | 2.81E+02 | 1.02E-02 | 1.44E-01 | 1.70E+00 | 1.70E+00 | 3.73E+01 | 2.19E+01 |
| Eif2ak1 | NM_013223 | 1.42E+02 | 2.09E-02 | 1.91E-01 | 1.70E+00 | 1.70E+00 | 1.89E+01 | 1.11E+01 |
| Nucb1 | NM_053463 | 1.76E+03 | 2.32E-03 | 1.00E-01 | 1.70E+00 | 1.70E+00 | 2.34E+02 | 1.37E+02 |
| Lsr | NM_032616 | 6.29E+02 | 6.14E-03 | 1.27E-01 | 1.70E+00 | 1.70E+00 | 8.36E+01 | 4.91E+01 |
| Lpcat3 | NM_001012189 | 3.37E+02 | 2.40E-03 | 1.02E-01 | 1.71E+00 | 1.71E+00 | 4.48E+01 | 2.63E+01 |
| Litaf | NM_001105735 | 9.69E+02 | 1.01E-02 | 1.43E-01 | 1.71E+00 | 1.71E+00 | 1.29E+02 | 7.56E+01 |
| Tasp1 | NM_001044243 | 3.68E+01 | 8.73E-03 | 1.36E-01 | 1.71E+00 | 1.71E+00 | 4.90E+00 | 2.87E+00 |
| Bace1 | NM_019204 | 4.38E+01 | 3.03E-03 | 1.10E-01 | 1.71E+00 | 1.71E+00 | 5.83E+00 | 3.42E+00 |
| Tram1 | NM_001007701 | 1.57E+03 | 7.70E-03 | 1.32E-01 | 1.71E+00 | 1.71E+00 | 2.08E+02 | 1.22E+02 |
| Txndc5 | NM_001271330 | 8.98E+02 | 5.28E-04 | 6.47E-02 | 1.71E+00 | 1.71E+00 | 1.20E+02 | 7.00E+01 |
| Nucb2 | NM_021663 | 3.18E+02 | 1.08E-03 | 7.99E-02 | 1.71E+00 | 1.71E+00 | 4.23E+01 | 2.47E+01 |
| Hspa5 | NM_013083 | 3.98E+03 | 7.37E-03 | 1.32E-01 | 1.71E+00 | 1.71E+00 | 5.29E+02 | 3.10E+02 |
| Hltf | NM_001106478 | 2.07E+02 | 1.40E-02 | 1.63E-01 | 1.71E+00 | 1.71E+00 | 2.75E+01 | 1.61E+01 |
| Ccdc86 | NM_001006974 | 5.49E+01 | 7.48E-03 | 1.32E-01 | 1.71E+00 | 1.71E+00 | 7.31E+00 | 4.28E+00 |
| Laptm4a | NM_199384 | 3.21E+03 | 2.19E-02 | 1.93E-01 | 1.71E+00 | 1.71E+00 | 4.28E+02 | 2.50E+02 |
| Zdhhc9 | NM_001039016 | 3.12E+02 | 9.38E-04 | 7.90E-02 | 1.72E+00 | 1.72E+00 | 4.17E+01 | 2.43E+01 |
| RT1-Ba | NM_001008831 | 8.20E+02 | 6.26E-03 | 1.28E-01 | 1.72E+00 | 1.72E+00 | 1.09E+02 | 6.37E+01 |
| Med1 | NM_001134361 | 8.90E+01 | 2.29E-02 | 1.97E-01 | 1.73E+00 | 1.73E+00 | 1.19E+01 | 6.90E+00 |
| Tmem47 | NM_001109317 | 1.01E+03 | 1.26E-02 | 1.55E-01 | 1.73E+00 | 1.73E+00 | 1.36E+02 | 7.85E+01 |
| Ebag9 | NM_001009665 | 2.15E+02 | 5.27E-03 | 1.24E-01 | 1.73E+00 | 1.73E+00 | 2.88E+01 | 1.67E+01 |
| Cd164 | NM_031812 | 3.44E+03 | 4.10E-03 | 1.18E-01 | 1.73E+00 | 1.73E+00 | 4.60E+02 | 2.66E+02 |
| Rnf121 | NM_001107540 | 5.86E+01 | 1.01E-02 | 1.43E-01 | 1.73E+00 | 1.73E+00 | 7.85E+00 | 4.54E+00 |
| Snrpb | NM_134358 | 5.58E+02 | 2.30E-03 | 9.95E-02 | 1.73E+00 | 1.73E+00 | 7.48E+01 | 4.32E+01 |
| Tob2 | NM_001007146 | 9.46E+01 | 2.14E-03 | 9.95E-02 | 1.73E+00 | 1.73E+00 | 1.27E+01 | 7.31E+00 |
| Lrrc59 | NM_001008280 | 6.22E+02 | 3.73E-03 | 1.17E-01 | 1.74E+00 | 1.74E+00 | 8.34E+01 | 4.81E+01 |
| Rhoa | NM_057132 | 6.46E+02 | 1.44E-02 | 1.65E-01 | 1.74E+00 | 1.74E+00 | 8.67E+01 | 4.99E+01 |
| Pbx3 | NM_001107834 | 7.71E+01 | 1.15E-02 | 1.49E-01 | 1.74E+00 | 1.74E+00 | 1.04E+01 | 5.95E+00 |
| Slc17a5 | NM_001009713 | 8.61E+01 | 8.93E-03 | 1.38E-01 | 1.74E+00 | 1.74E+00 | 1.16E+01 | 6.64E+00 |
| Agpat5 | NM_001134744 | 1.12E+02 | 1.20E-02 | 1.52E-01 | 1.74E+00 | 1.74E+00 | 1.51E+01 | 8.67E+00 |
| Wsb1 | NM_001042561 | 2.12E+02 | 2.45E-03 | 1.03E-01 | 1.74E+00 | 1.74E+00 | 2.85E+01 | 1.64E+01 |
| Galnt2 | NM_001106196 | 6.07E+02 | 3.44E-03 | 1.12E-01 | 1.74E+00 | 1.74E+00 | 8.16E+01 | 4.68E+01 |
| Tmem115 | NM_001108779 | 2.68E+02 | 7.80E-03 | 1.32E-01 | 1.75E+00 | 1.75E+00 | 3.60E+01 | 2.06E+01 |
| Pofut2 | NM_001107621 | 3.63E+02 | 5.03E-03 | 1.24E-01 | 1.75E+00 | 1.75E+00 | 4.88E+01 | 2.79E+01 |
| Zscan25 | NM_001108851 | 3.48E+01 | 5.73E-03 | 1.26E-01 | 1.75E+00 | 1.75E+00 | 4.68E+00 | 2.68E+00 |
| Ptgfrn | NM_019243 | 7.67E+02 | 1.36E-02 | 1.61E-01 | 1.75E+00 | 1.75E+00 | 1.03E+02 | 5.91E+01 |
| Slc35e1 | NM_001109107 | 1.99E+02 | 6.16E-04 | 6.62E-02 | 1.75E+00 | 1.75E+00 | 2.68E+01 | 1.53E+01 |
| Tmem19 | NM_199098 | 1.41E+02 | 3.44E-03 | 1.12E-01 | 1.75E+00 | 1.75E+00 | 1.90E+01 | 1.09E+01 |
| Rnf26 | NM_001113748 | 2.10E+02 | 2.74E-03 | 1.07E-01 | 1.75E+00 | 1.75E+00 | 2.83E+01 | 1.62E+01 |
| Dnajb14 | NM_001109193 | 3.61E+01 | 6.06E-03 | 1.26E-01 | 1.75E+00 | 1.75E+00 | 4.86E+00 | 2.78E+00 |
| Abcd1 | NM_001108821 | 1.35E+02 | 5.26E-04 | 6.47E-02 | 1.75E+00 | 1.75E+00 | 1.81E+01 | 1.04E+01 |
| Bsg | NM_012783 | 1.81E+03 | 3.94E-03 | 1.17E-01 | 1.75E+00 | 1.75E+00 | 2.43E+02 | 1.39E+02 |
| Rnh1 | NM_139105 | 2.82E+02 | 1.14E-03 | 8.00E-02 | 1.76E+00 | 1.76E+00 | 3.81E+01 | 2.17E+01 |
| Tanc1 | NM_001002854 | 1.34E+02 | 1.54E-02 | 1.69E-01 | 1.76E+00 | 1.76E+00 | 1.80E+01 | 1.03E+01 |
| Slc30a4 | NM_172066 | 5.88E+02 | 9.60E-03 | 1.42E-01 | 1.76E+00 | 1.76E+00 | 7.92E+01 | 4.51E+01 |
| Cgnl1 | NM_001108164 | 6.84E+02 | 2.16E-03 | 9.95E-02 | 1.76E+00 | 1.76E+00 | 9.22E+01 | 5.25E+01 |
| Yif1b | NM_198734.1 | 4.74E+01 | 9.18E-03 | 1.39E-01 | 1.76E+00 | 1.76E+00 | 6.39E+00 | 3.64E+00 |
| Yif1b | NM_198734 | 4.74E+01 | 9.18E-03 | 1.39E-01 | 1.76E+00 | 1.76E+00 | 6.39E+00 | 3.64E+00 |
| Atm | NM_001106821 | 8.64E+01 | 3.83E-03 | 1.17E-01 | 1.76E+00 | 1.76E+00 | 1.17E+01 | 6.63E+00 |
| Zw10 | NM_001024801 | 5.91E+01 | 6.17E-03 | 1.27E-01 | 1.76E+00 | 1.76E+00 | 7.98E+00 | 4.54E+00 |
| Mfsd14b | NM_001107334 | 3.45E+02 | 1.03E-02 | 1.44E-01 | 1.76E+00 | 1.76E+00 | 4.65E+01 | 2.64E+01 |
| RGD1309079 | NM_001134472 | 2.32E+02 | 2.22E-02 | 1.94E-01 | 1.76E+00 | 1.76E+00 | 3.13E+01 | 1.78E+01 |
| Hyou1 | NM_001034028 | 5.68E+02 | 1.13E-02 | 1.49E-01 | 1.77E+00 | 1.77E+00 | 7.68E+01 | 4.35E+01 |
| Col18a1 | NM_053489 | 1.80E+03 | 7.23E-03 | 1.32E-01 | 1.77E+00 | 1.77E+00 | 2.43E+02 | 1.37E+02 |
| Lsm2 | NM_001165922 | 1.00E+02 | 4.96E-03 | 1.23E-01 | 1.77E+00 | 1.77E+00 | 1.36E+01 | 7.68E+00 |
| Galnt4 | NM_001025053 | 5.40E+01 | 2.28E-02 | 1.97E-01 | 1.77E+00 | 1.77E+00 | 7.31E+00 | 4.13E+00 |
| Arl1 | NM_022385 | 1.60E+02 | 2.19E-03 | 9.95E-02 | 1.77E+00 | 1.77E+00 | 2.17E+01 | 1.23E+01 |
| Cnep1r1 | NM_001106173 | 7.77E+01 | 1.17E-03 | 8.06E-02 | 1.77E+00 | 1.77E+00 | 1.05E+01 | 5.94E+00 |
| Lrfn3 | NM_001107502 | 2.95E+01 | 6.75E-03 | 1.30E-01 | 1.77E+00 | 1.77E+00 | 4.00E+00 | 2.26E+00 |
| Clu | NM_053021 | 4.62E+03 | 3.16E-03 | 1.10E-01 | 1.77E+00 | 1.77E+00 | 6.25E+02 | 3.53E+02 |
| Slc9a8 | NM_001025281 | 3.06E+01 | 1.83E-02 | 1.82E-01 | 1.77E+00 | 1.77E+00 | 4.15E+00 | 2.34E+00 |
| Cnpy3 | NM_001134710 | 3.41E+02 | 3.09E-03 | 1.10E-01 | 1.78E+00 | 1.78E+00 | 4.62E+01 | 2.60E+01 |
| Tm9sf4 | NM_001025649 | 1.27E+02 | 6.52E-03 | 1.29E-01 | 1.78E+00 | 1.78E+00 | 1.73E+01 | 9.71E+00 |
| Ptdss1 | NM_001012113 | 2.48E+02 | 7.56E-03 | 1.32E-01 | 1.78E+00 | 1.78E+00 | 3.37E+01 | 1.89E+01 |
| Slc12a6 | NM_001109630 | 9.30E+01 | 2.49E-03 | 1.03E-01 | 1.78E+00 | 1.78E+00 | 1.26E+01 | 7.09E+00 |
| Pdzd8 | NM_001107446 | 2.01E+02 | 3.82E-03 | 1.17E-01 | 1.78E+00 | 1.78E+00 | 2.72E+01 | 1.53E+01 |
| Atp6v0a2 | NM_053775 | 4.20E+01 | 7.33E-03 | 1.32E-01 | 1.78E+00 | 1.78E+00 | 5.71E+00 | 3.20E+00 |
| Tmem30b | NM_001080380 | 1.70E+02 | 1.42E-02 | 1.64E-01 | 1.78E+00 | 1.78E+00 | 2.31E+01 | 1.30E+01 |
| Rrs1 | NM_001079699 | 1.02E+02 | 1.33E-03 | 8.31E-02 | 1.78E+00 | 1.78E+00 | 1.38E+01 | 7.75E+00 |
| Socs5 | NM_001109274 | 6.33E+02 | 2.20E-02 | 1.93E-01 | 1.79E+00 | 1.79E+00 | 8.61E+01 | 4.82E+01 |
| Itm2c | NM_001009674 | 1.90E+03 | 2.26E-02 | 1.96E-01 | 1.79E+00 | 1.79E+00 | 2.58E+02 | 1.45E+02 |
| Ppib | NM_022536 | 1.69E+03 | 9.17E-04 | 7.86E-02 | 1.79E+00 | 1.79E+00 | 2.30E+02 | 1.29E+02 |
| Tmem203 | NM_001107819 | 1.07E+02 | 7.85E-03 | 1.32E-01 | 1.79E+00 | 1.79E+00 | 1.46E+01 | 8.15E+00 |
| Reln | NM_080394 | 3.67E+02 | 1.39E-02 | 1.62E-01 | 1.79E+00 | 1.79E+00 | 4.99E+01 | 2.79E+01 |
| Adgrl2 | NM_001302212 | 1.86E+02 | 2.99E-03 | 1.10E-01 | 1.79E+00 | 1.79E+00 | 2.54E+01 | 1.41E+01 |
| Stt3a | NM_001134749 | 9.25E+02 | 2.11E-03 | 9.95E-02 | 1.79E+00 | 1.79E+00 | 1.26E+02 | 7.02E+01 |
| Tmem132a | NM_178021 | 3.84E+02 | 1.87E-02 | 1.84E-01 | 1.79E+00 | 1.79E+00 | 5.23E+01 | 2.92E+01 |
| Pigw | NM_194461 | 5.90E+01 | 1.67E-02 | 1.75E-01 | 1.79E+00 | 1.79E+00 | 8.03E+00 | 4.48E+00 |
| Rac2 | NM_001008384 | 1.71E+02 | 9.54E-04 | 7.90E-02 | 1.80E+00 | 1.80E+00 | 2.32E+01 | 1.29E+01 |
| Mesd | NM_001008345 | 4.96E+02 | 6.20E-03 | 1.27E-01 | 1.80E+00 | 1.80E+00 | 6.76E+01 | 3.76E+01 |
| Cd47 | NM_019195 | 3.66E+02 | 3.07E-03 | 1.10E-01 | 1.80E+00 | 1.80E+00 | 4.99E+01 | 2.78E+01 |
| Ppm1l | NM_001107681 | 3.74E+02 | 2.32E-02 | 1.98E-01 | 1.80E+00 | 1.80E+00 | 5.10E+01 | 2.83E+01 |
| Ephb6 | NM_001107857 | 1.64E+02 | 4.91E-03 | 1.23E-01 | 1.80E+00 | 1.80E+00 | 2.24E+01 | 1.24E+01 |
| Minpp1 | NM_019263 | 2.98E+02 | 1.53E-04 | 6.47E-02 | 1.80E+00 | 1.80E+00 | 4.07E+01 | 2.26E+01 |
| Calu | NM_022535 | 8.21E+02 | 1.91E-02 | 1.85E-01 | 1.80E+00 | 1.80E+00 | 1.12E+02 | 6.21E+01 |
| Fbxo46 | NM_001025642 | 1.13E+02 | 7.97E-03 | 1.33E-01 | 1.81E+00 | 1.81E+00 | 1.54E+01 | 8.50E+00 |
| Steap3 | NM_133314 | 2.16E+02 | 1.11E-02 | 1.47E-01 | 1.81E+00 | 1.81E+00 | 2.95E+01 | 1.63E+01 |
| Vegfc | NM_053653 | 5.52E+01 | 1.48E-02 | 1.67E-01 | 1.81E+00 | 1.81E+00 | 7.56E+00 | 4.16E+00 |
| RT1-Db1 | NM_001008884 | 1.14E+03 | 4.45E-03 | 1.19E-01 | 1.82E+00 | 1.82E+00 | 1.57E+02 | 8.62E+01 |
| Det1 | NM_001037194 | 2.43E+01 | 1.96E-03 | 9.67E-02 | 1.82E+00 | 1.82E+00 | 3.33E+00 | 1.83E+00 |
| Arl6ip6 | NM_001024310 | 1.03E+02 | 1.54E-02 | 1.69E-01 | 1.82E+00 | 1.82E+00 | 1.41E+01 | 7.73E+00 |
| LOC100911498 | NR_132635 | 5.64E+03 | 1.31E-02 | 1.57E-01 | 1.82E+00 | 1.82E+00 | 7.74E+02 | 4.25E+02 |
| Exd2 | NM_001108715 | 1.44E+02 | 9.77E-03 | 1.43E-01 | 1.82E+00 | 1.82E+00 | 1.97E+01 | 1.08E+01 |
| Manba | NM_001031655 | 1.36E+02 | 1.64E-04 | 6.47E-02 | 1.82E+00 | 1.82E+00 | 1.86E+01 | 1.02E+01 |
| Ifnar1 | NM_001105893 | 3.28E+02 | 6.82E-03 | 1.31E-01 | 1.82E+00 | 1.82E+00 | 4.50E+01 | 2.47E+01 |
| Spcs2 | NM_001191601 | 8.04E+02 | 3.91E-03 | 1.17E-01 | 1.83E+00 | 1.83E+00 | 1.10E+02 | 6.04E+01 |
| RT1-DMa | NM_198741.1 | 7.28E+01 | 9.07E-03 | 1.38E-01 | 1.83E+00 | 1.83E+00 | 1.00E+01 | 5.47E+00 |
| P2rx7 | NM_019256 | 7.67E+01 | 1.25E-02 | 1.55E-01 | 1.83E+00 | 1.83E+00 | 1.05E+01 | 5.76E+00 |
| Gla | NM_001108820 | 1.03E+02 | 1.50E-03 | 8.43E-02 | 1.83E+00 | 1.83E+00 | 1.41E+01 | 7.71E+00 |
| Srd5a3 | NM_001013990 | 1.19E+02 | 1.13E-02 | 1.49E-01 | 1.83E+00 | 1.83E+00 | 1.64E+01 | 8.96E+00 |
| Atp1b1 | NM_013113 | 1.72E+02 | 7.33E-03 | 1.32E-01 | 1.84E+00 | 1.84E+00 | 2.37E+01 | 1.29E+01 |
| Impad1 | NM_001008772 | 2.84E+02 | 7.55E-03 | 1.32E-01 | 1.84E+00 | 1.84E+00 | 3.91E+01 | 2.13E+01 |
| Actb | NM_031144 | 5.86E+03 | 3.22E-03 | 1.11E-01 | 1.84E+00 | 1.84E+00 | 8.07E+02 | 4.39E+02 |
| M6pr | NM_001007700.1 | 4.63E+02 | 9.76E-04 | 7.90E-02 | 1.84E+00 | 1.84E+00 | 6.38E+01 | 3.47E+01 |
| Hmgb2l1 | NM_001329881 | 3.74E+02 | 2.05E-02 | 1.89E-01 | 1.85E+00 | 1.85E+00 | 5.16E+01 | 2.80E+01 |
| Dtwd2 | NM_001108431 | 2.79E+01 | 1.65E-02 | 1.75E-01 | 1.85E+00 | 1.85E+00 | 3.85E+00 | 2.09E+00 |
| Tmed7 | NM_001105758 | 5.46E+02 | 1.62E-03 | 8.83E-02 | 1.85E+00 | 1.85E+00 | 7.53E+01 | 4.08E+01 |
| Rcn2 | NM_017132 | 4.21E+02 | 1.51E-02 | 1.68E-01 | 1.86E+00 | 1.86E+00 | 5.83E+01 | 3.13E+01 |
| Cd59 | NM_012925 | 8.80E+02 | 1.59E-02 | 1.71E-01 | 1.86E+00 | 1.86E+00 | 1.22E+02 | 6.54E+01 |
| Dnajb4 | NM_001013076 | 9.42E+01 | 1.78E-02 | 1.81E-01 | 1.86E+00 | 1.86E+00 | 1.30E+01 | 7.00E+00 |
| Adam9 | NM_001014772 | 1.81E+02 | 6.29E-03 | 1.28E-01 | 1.87E+00 | 1.87E+00 | 2.51E+01 | 1.35E+01 |
| Itgb1 | NM_017022 | 2.53E+03 | 1.65E-02 | 1.75E-01 | 1.87E+00 | 1.87E+00 | 3.51E+02 | 1.88E+02 |
| Pcna | NM_022381 | 3.52E+02 | 4.70E-03 | 1.21E-01 | 1.87E+00 | 1.87E+00 | 4.88E+01 | 2.61E+01 |
| Ctsl | NM_013156 | 2.27E+03 | 9.29E-03 | 1.41E-01 | 1.87E+00 | 1.87E+00 | 3.15E+02 | 1.68E+02 |
| Zhx2 | NM_001271056 | 6.32E+01 | 2.27E-02 | 1.97E-01 | 1.87E+00 | 1.87E+00 | 8.77E+00 | 4.69E+00 |
| Smad6 | NM_001109002 | 4.59E+01 | 4.52E-03 | 1.19E-01 | 1.87E+00 | 1.87E+00 | 6.37E+00 | 3.41E+00 |
| Ctns | NM_001191647 | 5.80E+01 | 2.83E-03 | 1.08E-01 | 1.87E+00 | 1.87E+00 | 8.05E+00 | 4.30E+00 |
| P4ha1 | NM_172062 | 1.17E+02 | 3.12E-03 | 1.10E-01 | 1.87E+00 | 1.87E+00 | 1.63E+01 | 8.69E+00 |
| RT1-DMb | NM_198740.1 | 9.08E+01 | 5.84E-03 | 1.26E-01 | 1.88E+00 | 1.88E+00 | 1.26E+01 | 6.72E+00 |
| RT1-DMb | NM_198740 | 9.08E+01 | 5.84E-03 | 1.26E-01 | 1.88E+00 | 1.88E+00 | 1.26E+01 | 6.72E+00 |
| Atp1a1 | NM_012504 | 1.53E+03 | 2.88E-04 | 6.47E-02 | 1.88E+00 | 1.88E+00 | 2.13E+02 | 1.13E+02 |
| Mpzl1 | NM_001007728 | 6.81E+02 | 1.96E-02 | 1.86E-01 | 1.88E+00 | 1.88E+00 | 9.47E+01 | 5.04E+01 |
| Tenm3 | NM_001169133 | 6.35E+01 | 1.07E-02 | 1.45E-01 | 1.88E+00 | 1.88E+00 | 8.83E+00 | 4.70E+00 |
| Bmpr1a | NM_030849 | 1.64E+02 | 3.39E-03 | 1.12E-01 | 1.88E+00 | 1.88E+00 | 2.28E+01 | 1.21E+01 |
| RT1-A3 | NM_001008830 | 8.77E+01 | 1.17E-02 | 1.51E-01 | 1.89E+00 | 1.89E+00 | 1.22E+01 | 6.47E+00 |
| RT1-DMa | NM_198741 | 7.44E+01 | 7.71E-03 | 1.32E-01 | 1.90E+00 | 1.90E+00 | 1.04E+01 | 5.47E+00 |
| Cybrd1 | NM_001011954 | 5.08E+01 | 1.12E-02 | 1.48E-01 | 1.90E+00 | 1.90E+00 | 7.10E+00 | 3.74E+00 |
| Cd74 | NM_013069 | 3.18E+03 | 4.06E-03 | 1.18E-01 | 1.90E+00 | 1.90E+00 | 4.45E+02 | 2.34E+02 |
| Mfsd1 | NM_001191847 | 2.04E+02 | 6.96E-04 | 6.85E-02 | 1.90E+00 | 1.90E+00 | 2.85E+01 | 1.50E+01 |
| Slc3a2 | NM_001271089 | 1.82E+03 | 1.08E-02 | 1.45E-01 | 1.90E+00 | 1.90E+00 | 2.55E+02 | 1.34E+02 |
| Tapbpl | NM_001106622 | 1.96E+02 | 5.81E-04 | 6.47E-02 | 1.91E+00 | 1.91E+00 | 2.75E+01 | 1.44E+01 |
| M6pr | NM_001007700 | 4.69E+02 | 5.17E-04 | 6.47E-02 | 1.91E+00 | 1.91E+00 | 6.57E+01 | 3.44E+01 |
| Ssr2 | NM_001106442 | 7.76E+02 | 4.13E-03 | 1.19E-01 | 1.91E+00 | 1.91E+00 | 1.09E+02 | 5.69E+01 |
| Calr | NM_022399 | 4.68E+03 | 4.06E-03 | 1.18E-01 | 1.92E+00 | 1.92E+00 | 6.57E+02 | 3.43E+02 |
| Nr3c2 | NM_013131 | 1.49E+02 | 3.90E-03 | 1.17E-01 | 1.92E+00 | 1.92E+00 | 2.09E+01 | 1.09E+01 |
| Grn | NM_017113 | 9.35E+02 | 4.77E-04 | 6.47E-02 | 1.92E+00 | 1.92E+00 | 1.31E+02 | 6.84E+01 |
| Fstl1 | NM_024369 | 5.67E+02 | 4.52E-03 | 1.19E-01 | 1.92E+00 | 1.92E+00 | 7.96E+01 | 4.14E+01 |
| Timp2 | NM_021989 | 1.18E+03 | 1.93E-02 | 1.85E-01 | 1.93E+00 | 1.93E+00 | 1.66E+02 | 8.61E+01 |
| Htra1 | NM_031721 | 1.02E+03 | 1.56E-02 | 1.70E-01 | 1.93E+00 | 1.93E+00 | 1.43E+02 | 7.41E+01 |
| Id2 | NM_013060 | 2.01E+02 | 9.74E-03 | 1.43E-01 | 1.93E+00 | 1.93E+00 | 2.83E+01 | 1.46E+01 |
| Gpc4 | NM_001014108 | 4.57E+02 | 2.72E-03 | 1.07E-01 | 1.93E+00 | 1.93E+00 | 6.43E+01 | 3.33E+01 |
| C1r | NM_001134555 | 2.21E+03 | 5.47E-03 | 1.25E-01 | 1.94E+00 | 1.94E+00 | 3.11E+02 | 1.61E+02 |
| Ermp1 | NM_184050 | 4.66E+02 | 6.33E-03 | 1.28E-01 | 1.94E+00 | 1.94E+00 | 6.57E+01 | 3.39E+01 |
| Serpinh1 | NM_017173 | 2.93E+03 | 1.66E-02 | 1.75E-01 | 1.94E+00 | 1.94E+00 | 4.13E+02 | 2.13E+02 |
| Tor4a | NM_001107816 | 6.93E+01 | 6.11E-03 | 1.27E-01 | 1.95E+00 | 1.95E+00 | 9.79E+00 | 5.03E+00 |
| Stt3b | NM_001170539 | 1.78E+02 | 4.38E-03 | 1.19E-01 | 1.95E+00 | 1.95E+00 | 2.51E+01 | 1.29E+01 |
| Ankle2 | NM_001047901 | 5.59E+01 | 1.12E-03 | 8.00E-02 | 1.95E+00 | 1.95E+00 | 7.89E+00 | 4.05E+00 |
| Lyn | NM_001111098 | 3.23E+02 | 8.41E-03 | 1.34E-01 | 1.95E+00 | 1.95E+00 | 4.56E+01 | 2.34E+01 |
| Tmx1 | NM_001024800 | 1.76E+02 | 2.84E-03 | 1.08E-01 | 1.95E+00 | 1.95E+00 | 2.49E+01 | 1.28E+01 |
| Mmp14 | NM_031056 | 7.19E+02 | 1.66E-02 | 1.75E-01 | 1.95E+00 | 1.95E+00 | 1.02E+02 | 5.21E+01 |
| RT1-CE16 | NM_001008839 | 2.38E+02 | 4.39E-03 | 1.19E-01 | 1.96E+00 | 1.96E+00 | 3.37E+01 | 1.72E+01 |
| Mbtps1 | NM_053569 | 6.64E+02 | 1.92E-03 | 9.67E-02 | 1.96E+00 | 1.96E+00 | 9.40E+01 | 4.80E+01 |
| Tmed10 | NM_053467 | 9.61E+02 | 4.09E-04 | 6.47E-02 | 1.96E+00 | 1.96E+00 | 1.36E+02 | 6.94E+01 |
| Ckap4 | NM_001108740 | 2.80E+02 | 1.51E-02 | 1.68E-01 | 1.96E+00 | 1.96E+00 | 3.97E+01 | 2.02E+01 |
| Plek | NM_001025750 | 4.49E+01 | 1.07E-02 | 1.45E-01 | 1.96E+00 | 1.96E+00 | 6.36E+00 | 3.24E+00 |
| Man1a2 | NM_001106452 | 9.91E+01 | 7.53E-03 | 1.32E-01 | 1.96E+00 | 1.96E+00 | 1.40E+01 | 7.15E+00 |
| Itgal | NM_001033998 | 4.13E+01 | 1.44E-03 | 8.33E-02 | 1.97E+00 | 1.97E+00 | 5.85E+00 | 2.98E+00 |
| Mfsd6 | NM_001106911 | 2.01E+02 | 5.83E-03 | 1.26E-01 | 1.97E+00 | 1.97E+00 | 2.86E+01 | 1.45E+01 |
| Olfml3 | NM_001107708 | 2.48E+02 | 7.02E-03 | 1.32E-01 | 1.97E+00 | 1.97E+00 | 3.51E+01 | 1.79E+01 |
| Fuca2 | NM_001004218 | 1.13E+02 | 2.07E-03 | 9.87E-02 | 1.97E+00 | 1.97E+00 | 1.60E+01 | 8.12E+00 |
| Cd53 | NM_012523 | 1.31E+02 | 1.77E-03 | 9.32E-02 | 1.98E+00 | 1.98E+00 | 1.86E+01 | 9.41E+00 |
| Atp6ap2 | NM_001007091 | 8.23E+01 | 6.03E-03 | 1.26E-01 | 1.98E+00 | 1.98E+00 | 1.17E+01 | 5.92E+00 |
| Slc44a1 | NM_053492 | 1.21E+02 | 6.46E-03 | 1.29E-01 | 1.98E+00 | 1.98E+00 | 1.72E+01 | 8.65E+00 |
| Clcn7 | NM_031568 | 6.68E+01 | 1.89E-03 | 9.66E-02 | 1.99E+00 | 1.99E+00 | 9.52E+00 | 4.79E+00 |
| Srebf1 | NM_001276708 | 1.55E+03 | 2.11E-02 | 1.91E-01 | 1.99E+00 | 1.99E+00 | 2.20E+02 | 1.11E+02 |
| RT1-Da | NM_001008847 | 1.10E+03 | 2.25E-03 | 9.95E-02 | 1.99E+00 | 1.99E+00 | 1.57E+02 | 7.88E+01 |
| Scarb2 | NM_054001 | 2.14E+02 | 4.35E-04 | 6.47E-02 | 1.99E+00 | 1.99E+00 | 3.06E+01 | 1.53E+01 |
| Frzb | NM_001100527 | 2.15E+02 | 1.54E-02 | 1.69E-01 | 2.00E+00 | 2.00E+00 | 3.06E+01 | 1.53E+01 |
| Slc7a5 | NM_017353 | 9.52E+01 | 6.04E-03 | 1.26E-01 | 2.00E+00 | 2.00E+00 | 1.36E+01 | 6.81E+00 |
| Ctsh | NM_012939 | 1.74E+03 | 3.25E-03 | 1.11E-01 | 2.00E+00 | 2.00E+00 | 2.48E+02 | 1.24E+02 |
| Ifitm3 | NM_001136124 | 3.31E+03 | 2.71E-03 | 1.07E-01 | 2.00E+00 | 2.00E+00 | 4.73E+02 | 2.37E+02 |
| Alcam | NM_031753 | 2.02E+02 | 1.23E-02 | 1.54E-01 | 2.00E+00 | 2.00E+00 | 2.88E+01 | 1.44E+01 |
| Pdia4 | NM_053849 | 8.44E+02 | 8.02E-03 | 1.33E-01 | 2.00E+00 | 2.00E+00 | 1.21E+02 | 6.03E+01 |
| Ptprf | NM_019249 | 6.73E+02 | 4.40E-03 | 1.19E-01 | 2.00E+00 | 2.00E+00 | 9.62E+01 | 4.81E+01 |
| Edem3 | NM_001191671 | 3.84E+01 | 2.18E-02 | 1.93E-01 | 2.00E+00 | 2.00E+00 | 5.48E+00 | 2.74E+00 |
| Slc38a7 | NM_001003705 | 2.36E+01 | 3.06E-03 | 1.10E-01 | 2.00E+00 | 2.00E+00 | 3.37E+00 | 1.68E+00 |
| Pim1 | NM_017034 | 3.49E+01 | 1.99E-02 | 1.87E-01 | 2.01E+00 | 2.01E+00 | 4.99E+00 | 2.48E+00 |
| Rdh10 | NM_181478 | 4.65E+01 | 2.19E-02 | 1.93E-01 | 2.01E+00 | 2.01E+00 | 6.66E+00 | 3.31E+00 |
| Prelp | NM_053385 | 3.94E+02 | 1.38E-02 | 1.62E-01 | 2.01E+00 | 2.01E+00 | 5.65E+01 | 2.80E+01 |
| Ppt1 | NM_022502 | 1.55E+02 | 8.99E-04 | 7.79E-02 | 2.02E+00 | 2.02E+00 | 2.22E+01 | 1.10E+01 |
| Man2a1 | NM_012979 | 6.32E+02 | 3.00E-03 | 1.10E-01 | 2.02E+00 | 2.02E+00 | 9.06E+01 | 4.49E+01 |
| Tapbp | NM_033098 | 2.75E+02 | 3.11E-04 | 6.47E-02 | 2.02E+00 | 2.02E+00 | 3.94E+01 | 1.95E+01 |
| Slc6a6 | NM_017206 | 5.81E+01 | 9.71E-03 | 1.43E-01 | 2.02E+00 | 2.02E+00 | 8.34E+00 | 4.13E+00 |
| Lhfp | NM_001109183 | 4.23E+02 | 1.50E-02 | 1.67E-01 | 2.02E+00 | 2.02E+00 | 6.06E+01 | 3.00E+01 |
| Yipf6 | NM_001025747 | 8.93E+01 | 6.02E-03 | 1.26E-01 | 2.03E+00 | 2.03E+00 | 1.28E+01 | 6.33E+00 |
| Ece1 | NM_053596 | 3.97E+02 | 1.00E-02 | 1.43E-01 | 2.03E+00 | 2.03E+00 | 5.70E+01 | 2.81E+01 |
| Impad1 | NM_001008772.1 | 2.17E+02 | 5.12E-03 | 1.24E-01 | 2.03E+00 | 2.03E+00 | 3.12E+01 | 1.53E+01 |
| Agtrap | NM_001007654 | 1.14E+02 | 7.76E-04 | 7.23E-02 | 2.04E+00 | 2.04E+00 | 1.64E+01 | 8.04E+00 |
| Degs1 | NM_053323 | 4.26E+02 | 5.06E-03 | 1.24E-01 | 2.04E+00 | 2.04E+00 | 6.14E+01 | 3.01E+01 |
| Itgb2 | NM_001037780 | 8.44E+01 | 1.54E-02 | 1.69E-01 | 2.05E+00 | 2.05E+00 | 1.22E+01 | 5.95E+00 |
| Zdhhc5 | NM_001039338 | 1.57E+02 | 1.05E-03 | 7.92E-02 | 2.05E+00 | 2.05E+00 | 2.26E+01 | 1.10E+01 |
| Abcc4 | NM_133411 | 2.46E+01 | 1.68E-02 | 1.75E-01 | 2.05E+00 | 2.05E+00 | 3.55E+00 | 1.73E+00 |
| Spint2 | NM_199087 | 2.80E+03 | 1.11E-02 | 1.47E-01 | 2.05E+00 | 2.05E+00 | 4.05E+02 | 1.97E+02 |
| Myadm | NM_183332 | 9.12E+02 | 8.18E-03 | 1.33E-01 | 2.06E+00 | 2.06E+00 | 1.32E+02 | 6.40E+01 |
| Arsb | NM_033443 | 1.00E+02 | 5.19E-04 | 6.47E-02 | 2.06E+00 | 2.06E+00 | 1.46E+01 | 7.05E+00 |
| Akap1 | NM_053665 | 2.04E+02 | 1.24E-03 | 8.06E-02 | 2.07E+00 | 2.07E+00 | 2.95E+01 | 1.43E+01 |
| Zmpste24 | NM_001107974 | 9.40E+01 | 1.08E-03 | 7.99E-02 | 2.07E+00 | 2.07E+00 | 1.36E+01 | 6.58E+00 |
| Fzd1 | NM_021266 | 5.38E+02 | 5.16E-03 | 1.24E-01 | 2.07E+00 | 2.07E+00 | 7.80E+01 | 3.76E+01 |
| Slc16a1 | NM_012716 | 1.36E+02 | 9.88E-03 | 1.43E-01 | 2.07E+00 | 2.07E+00 | 1.97E+01 | 9.49E+00 |
| RT1-CE7 | NM_001008845 | 2.50E+02 | 1.03E-03 | 7.92E-02 | 2.07E+00 | 2.07E+00 | 3.63E+01 | 1.75E+01 |
| Acvr1 | NM_024486 | 4.46E+01 | 6.27E-04 | 6.64E-02 | 2.08E+00 | 2.08E+00 | 6.47E+00 | 3.12E+00 |
| Acvr1b | NM_199230 | 4.40E+01 | 1.76E-02 | 1.80E-01 | 2.08E+00 | 2.08E+00 | 6.40E+00 | 3.07E+00 |
| Lamc1 | NM_053966 | 1.42E+03 | 1.50E-02 | 1.67E-01 | 2.08E+00 | 2.08E+00 | 2.06E+02 | 9.87E+01 |
| Ddost | NM_001012104 | 7.09E+02 | 4.19E-04 | 6.47E-02 | 2.10E+00 | 2.10E+00 | 1.03E+02 | 4.93E+01 |
| Tpst1 | NM_001011903 | 1.69E+02 | 9.92E-04 | 7.90E-02 | 2.10E+00 | 2.10E+00 | 2.46E+01 | 1.17E+01 |
| Pard6b | NM_001108609 | 6.74E+01 | 1.03E-02 | 1.44E-01 | 2.10E+00 | 2.10E+00 | 9.83E+00 | 4.68E+00 |
| Wfs1 | NM_031823 | 3.74E+01 | 3.09E-03 | 1.10E-01 | 2.11E+00 | 2.11E+00 | 5.46E+00 | 2.59E+00 |
| Acp5 | NM_019144 | 1.41E+02 | 1.14E-03 | 8.00E-02 | 2.11E+00 | 2.11E+00 | 2.06E+01 | 9.79E+00 |
| Lrrc8d | NM_001008338 | 2.58E+02 | 1.76E-05 | 5.02E-02 | 2.11E+00 | 2.11E+00 | 3.77E+01 | 1.78E+01 |
| Col5a1 | NM_134452 | 1.93E+02 | 1.71E-03 | 9.15E-02 | 2.11E+00 | 2.11E+00 | 2.82E+01 | 1.33E+01 |
| Chst14 | NM_001109639 | 1.05E+02 | 7.43E-03 | 1.32E-01 | 2.12E+00 | 2.12E+00 | 1.54E+01 | 7.29E+00 |
| Iqgap1 | NM_001108489 | 4.76E+02 | 1.37E-02 | 1.61E-01 | 2.12E+00 | 2.12E+00 | 6.98E+01 | 3.29E+01 |
| B3galt6 | NM_001106699 | 1.84E+01 | 2.36E-03 | 1.00E-01 | 2.12E+00 | 2.12E+00 | 2.69E+00 | 1.27E+00 |
| Sectm1b | NM_199082 | 5.17E+02 | 3.67E-03 | 1.17E-01 | 2.12E+00 | 2.12E+00 | 7.58E+01 | 3.57E+01 |
| Lyz2 | NM_012771 | 1.73E+03 | 5.28E-03 | 1.24E-01 | 2.12E+00 | 2.12E+00 | 2.53E+02 | 1.19E+02 |
| Man2a2 | NM_001107527 | 8.87E+01 | 1.77E-03 | 9.32E-02 | 2.12E+00 | 2.12E+00 | 1.30E+01 | 6.12E+00 |
| Ceacam1 | NM_001033860 | 1.02E+02 | 6.44E-03 | 1.29E-01 | 2.12E+00 | 2.12E+00 | 1.49E+01 | 7.02E+00 |
| Suco | NM_199403 | 7.05E+01 | 4.74E-04 | 6.47E-02 | 2.12E+00 | 2.12E+00 | 1.03E+01 | 4.86E+00 |
| Cptp | NM_001007703 | 1.02E+02 | 1.51E-02 | 1.68E-01 | 2.13E+00 | 2.13E+00 | 1.50E+01 | 7.04E+00 |
| Nrp1 | NM_145098 | 7.97E+01 | 1.33E-02 | 1.59E-01 | 2.13E+00 | 2.13E+00 | 1.17E+01 | 5.49E+00 |
| Tspan14 | NM_001169127 | 1.67E+02 | 4.00E-04 | 6.47E-02 | 2.13E+00 | 2.13E+00 | 2.45E+01 | 1.15E+01 |
| Foxo1 | NM_001191846 | 9.28E+01 | 1.53E-02 | 1.69E-01 | 2.14E+00 | 2.14E+00 | 1.36E+01 | 6.38E+00 |
| Angptl2 | NM_133569 | 1.51E+02 | 2.61E-03 | 1.06E-01 | 2.14E+00 | 2.14E+00 | 2.21E+01 | 1.04E+01 |
| Lemd3 | NM_001191000 | 6.20E+01 | 1.10E-03 | 8.00E-02 | 2.15E+00 | 2.15E+00 | 9.13E+00 | 4.24E+00 |
| Lman1 | NM_053886 | 9.87E+01 | 1.79E-02 | 1.81E-01 | 2.15E+00 | 2.15E+00 | 1.46E+01 | 6.75E+00 |
| Steap4 | NM_001044265 | 2.02E+03 | 1.20E-02 | 1.52E-01 | 2.16E+00 | 2.16E+00 | 2.99E+02 | 1.38E+02 |
| Gns | NM_001011989.1 | 5.32E+01 | 7.72E-03 | 1.32E-01 | 2.17E+00 | 2.17E+00 | 7.86E+00 | 3.63E+00 |
| Gns | NM_001011989 | 5.32E+01 | 7.72E-03 | 1.32E-01 | 2.17E+00 | 2.17E+00 | 7.86E+00 | 3.63E+00 |
| Lmnb1 | NM_053905 | 4.04E+01 | 1.95E-02 | 1.86E-01 | 2.17E+00 | 2.17E+00 | 5.97E+00 | 2.75E+00 |
| Nectin1 | NM_001100476 | 4.65E+02 | 2.22E-03 | 9.95E-02 | 2.17E+00 | 2.17E+00 | 6.87E+01 | 3.17E+01 |
| Slc41a1 | NM_001108855 | 4.67E+02 | 7.77E-03 | 1.32E-01 | 2.17E+00 | 2.17E+00 | 6.91E+01 | 3.18E+01 |
| Prdx4 | NM_053512 | 4.49E+02 | 2.94E-03 | 1.10E-01 | 2.18E+00 | 2.18E+00 | 6.64E+01 | 3.05E+01 |
| Lrrc8a | NM_001024782 | 1.71E+02 | 5.64E-04 | 6.47E-02 | 2.19E+00 | 2.19E+00 | 2.54E+01 | 1.16E+01 |
| Cxadr | NM_053570 | 7.93E+01 | 5.83E-03 | 1.26E-01 | 2.20E+00 | 2.20E+00 | 1.18E+01 | 5.36E+00 |
| Mtdh | NM_133398 | 1.22E+02 | 4.71E-03 | 1.21E-01 | 2.21E+00 | 2.21E+00 | 1.82E+01 | 8.22E+00 |
| Timm9 | NM_133604 | 5.47E+01 | 1.25E-02 | 1.55E-01 | 2.22E+00 | 2.22E+00 | 8.16E+00 | 3.68E+00 |
| Sypl1 | NM_001014263 | 1.59E+02 | 2.19E-03 | 9.95E-02 | 2.22E+00 | 2.22E+00 | 2.37E+01 | 1.07E+01 |
| Kcnj8 | NM_017099 | 7.40E+01 | 1.00E-02 | 1.43E-01 | 2.22E+00 | 2.22E+00 | 1.10E+01 | 4.97E+00 |
| Lgr4 | NM_173328 | 2.47E+02 | 2.77E-03 | 1.08E-01 | 2.22E+00 | 2.22E+00 | 3.69E+01 | 1.66E+01 |
| Slc43a2 | NM_001105812 | 1.79E+02 | 2.22E-04 | 6.47E-02 | 2.23E+00 | 2.23E+00 | 2.67E+01 | 1.20E+01 |
| Ctsc | NM_017097 | 9.53E+02 | 3.76E-04 | 6.47E-02 | 2.23E+00 | 2.23E+00 | 1.42E+02 | 6.40E+01 |
| RT1-CE10 | NM_001008833 | 4.07E+02 | 1.24E-03 | 8.06E-02 | 2.23E+00 | 2.23E+00 | 6.08E+01 | 2.72E+01 |
| LOC108348108 | NM_001329896 | 4.44E+02 | 3.40E-03 | 1.12E-01 | 2.23E+00 | 2.23E+00 | 6.64E+01 | 2.98E+01 |
| Sectm1a | NM_001013043 | 1.26E+02 | 1.90E-02 | 1.85E-01 | 2.24E+00 | 2.24E+00 | 1.89E+01 | 8.43E+00 |
| Slc30a7 | NM_001191715 | 6.83E+01 | 4.58E-03 | 1.20E-01 | 2.24E+00 | 2.24E+00 | 1.02E+01 | 4.57E+00 |
| Cmtr2 | NM_001106186 | 6.36E+01 | 1.05E-02 | 1.44E-01 | 2.24E+00 | 2.24E+00 | 9.52E+00 | 4.25E+00 |
| Sft2d2 | NM_001034011 | 6.44E+02 | 3.87E-04 | 6.47E-02 | 2.24E+00 | 2.24E+00 | 9.65E+01 | 4.31E+01 |
| Tmtc4 | NM_001134414 | 7.66E+01 | 7.06E-04 | 6.85E-02 | 2.24E+00 | 2.24E+00 | 1.15E+01 | 5.12E+00 |
| Arl6ip5 | NM_023972 | 9.68E+01 | 1.93E-03 | 9.67E-02 | 2.24E+00 | 2.24E+00 | 1.45E+01 | 6.46E+00 |
| Perp | NM_001106265 | 1.43E+03 | 5.66E-03 | 1.26E-01 | 2.25E+00 | 2.25E+00 | 2.14E+02 | 9.53E+01 |
| Dag1 | NM_053697 | 2.10E+03 | 4.73E-03 | 1.21E-01 | 2.26E+00 | 2.26E+00 | 3.16E+02 | 1.40E+02 |
| Islr | NM_001126300 | 3.04E+02 | 1.08E-02 | 1.45E-01 | 2.26E+00 | 2.26E+00 | 4.56E+01 | 2.02E+01 |
| Cxcl16 | NM_001017478 | 3.92E+02 | 1.38E-02 | 1.62E-01 | 2.27E+00 | 2.27E+00 | 5.89E+01 | 2.60E+01 |
| Dkk3 | NM_138519 | 5.10E+02 | 1.58E-02 | 1.71E-01 | 2.27E+00 | 2.27E+00 | 7.68E+01 | 3.38E+01 |
| Actn1 | NM_031005 | 3.07E+02 | 2.73E-03 | 1.07E-01 | 2.30E+00 | 2.30E+00 | 4.65E+01 | 2.02E+01 |
| Ostm1 | NM_001029925 | 4.81E+01 | 1.51E-03 | 8.43E-02 | 2.30E+00 | 2.30E+00 | 7.28E+00 | 3.16E+00 |
| Reep3 | NM_001106386 | 1.30E+02 | 3.08E-04 | 6.47E-02 | 2.31E+00 | 2.31E+00 | 1.97E+01 | 8.53E+00 |
| Pigm | NM_024144 | 6.53E+01 | 9.52E-04 | 7.90E-02 | 2.31E+00 | 2.31E+00 | 9.90E+00 | 4.28E+00 |
| Krt8 | NM_199370 | 2.08E+03 | 9.53E-03 | 1.42E-01 | 2.32E+00 | 2.32E+00 | 3.15E+02 | 1.36E+02 |
| Slc39a6 | NM_001024745 | 2.48E+02 | 1.05E-02 | 1.44E-01 | 2.33E+00 | 2.33E+00 | 3.77E+01 | 1.62E+01 |
| Mfge8 | NM_012811 | 6.15E+03 | 8.44E-03 | 1.34E-01 | 2.34E+00 | 2.34E+00 | 9.36E+02 | 4.01E+02 |
| Prnp | NM_012631 | 1.71E+03 | 1.09E-02 | 1.46E-01 | 2.34E+00 | 2.34E+00 | 2.60E+02 | 1.11E+02 |
| Fut8 | NM_001002289 | 7.86E+01 | 1.36E-03 | 8.31E-02 | 2.34E+00 | 2.34E+00 | 1.20E+01 | 5.12E+00 |
| Large1 | NM_001108439 | 1.26E+02 | 1.56E-03 | 8.68E-02 | 2.34E+00 | 2.34E+00 | 1.92E+01 | 8.19E+00 |
| Il1rn | NM_022194 | 6.20E+01 | 5.72E-03 | 1.26E-01 | 2.35E+00 | 2.35E+00 | 9.47E+00 | 4.03E+00 |
| Vsig10 | NM_001271336 | 1.01E+02 | 2.98E-03 | 1.10E-01 | 2.36E+00 | 2.36E+00 | 1.54E+01 | 6.52E+00 |
| Psen1 | NM_019163 | 4.87E+01 | 1.31E-03 | 8.31E-02 | 2.36E+00 | 2.36E+00 | 7.44E+00 | 3.15E+00 |
| Col14a1 | NM_001130548 | 7.28E+02 | 8.14E-03 | 1.33E-01 | 2.37E+00 | 2.37E+00 | 1.12E+02 | 4.70E+01 |
| Irf2bpl | NM_001012470 | 2.84E+02 | 1.96E-02 | 1.86E-01 | 2.41E+00 | 2.41E+00 | 4.38E+01 | 1.82E+01 |
| Erlin2 | NM_001106088 | 1.40E+02 | 1.41E-05 | 5.02E-02 | 2.42E+00 | 2.42E+00 | 2.17E+01 | 8.95E+00 |
| Tgfbr3 | NM_017256 | 2.42E+02 | 7.76E-03 | 1.32E-01 | 2.42E+00 | 2.42E+00 | 3.74E+01 | 1.54E+01 |
| Paqr4 | NM_001017377 | 1.74E+02 | 5.50E-03 | 1.25E-01 | 2.44E+00 | 2.44E+00 | 2.70E+01 | 1.11E+01 |
| Bace2 | NM_001002802 | 1.23E+02 | 3.96E-03 | 1.17E-01 | 2.45E+00 | 2.45E+00 | 1.90E+01 | 7.76E+00 |
| Tmx2 | NM_001007643 | 9.38E+01 | 8.45E-05 | 6.47E-02 | 2.49E+00 | 2.49E+00 | 1.46E+01 | 5.88E+00 |
| Met | NM_031517 | 7.02E+01 | 1.24E-02 | 1.55E-01 | 2.50E+00 | 2.50E+00 | 1.10E+01 | 4.39E+00 |
| Golim4 | NM_001191567 | 1.93E+02 | 9.90E-05 | 6.47E-02 | 2.50E+00 | 2.50E+00 | 3.01E+01 | 1.20E+01 |
| RT1-CE11 | NM_001008834 | 5.06E+01 | 3.99E-03 | 1.17E-01 | 2.50E+00 | 2.50E+00 | 7.90E+00 | 3.16E+00 |
| Spint2 | NM_001082549 | 6.93E+01 | 4.39E-03 | 1.19E-01 | 2.51E+00 | 2.51E+00 | 1.08E+01 | 4.32E+00 |
| Ptprg | NM_134356 | 7.22E+01 | 4.96E-04 | 6.47E-02 | 2.52E+00 | 2.52E+00 | 1.13E+01 | 4.49E+00 |
| Crim1 | NM_001169103 | 1.57E+02 | 6.47E-03 | 1.29E-01 | 2.52E+00 | 2.52E+00 | 2.46E+01 | 9.74E+00 |
| Cd14 | NM_021744 | 7.67E+02 | 4.49E-04 | 6.47E-02 | 2.52E+00 | 2.52E+00 | 1.20E+02 | 4.77E+01 |
| Hsd11b1 | NM_017080 | 1.78E+02 | 3.16E-04 | 6.47E-02 | 2.54E+00 | 2.54E+00 | 2.80E+01 | 1.10E+01 |
| Gpc3 | NM_012774 | 8.40E+01 | 2.28E-03 | 9.95E-02 | 2.55E+00 | 2.55E+00 | 1.32E+01 | 5.18E+00 |
| Ugt1a7c | NM_130407 | 1.01E+02 | 1.30E-03 | 8.31E-02 | 2.58E+00 | 2.58E+00 | 1.60E+01 | 6.20E+00 |
| Bmp7 | NM_001191856 | 1.76E+02 | 6.93E-03 | 1.31E-01 | 2.59E+00 | 2.59E+00 | 2.79E+01 | 1.08E+01 |
| Mfap3 | NM_001007609 | 2.32E+01 | 4.04E-03 | 1.18E-01 | 2.60E+00 | 2.60E+00 | 3.67E+00 | 1.41E+00 |
| Dgcr2 | NM_001012146 | 3.16E+01 | 1.01E-03 | 7.90E-02 | 2.62E+00 | 2.62E+00 | 5.03E+00 | 1.92E+00 |
| Dgcr2 | NM_001012146.1 | 3.16E+01 | 1.01E-03 | 7.90E-02 | 2.62E+00 | 2.62E+00 | 5.03E+00 | 1.92E+00 |
| Ikbip | NM_001009430 | 2.84E+01 | 4.61E-04 | 6.47E-02 | 2.65E+00 | 2.65E+00 | 4.53E+00 | 1.71E+00 |
| Pcyox1 | NM_145085 | 1.26E+02 | 1.04E-03 | 7.92E-02 | 2.66E+00 | 2.66E+00 | 2.01E+01 | 7.55E+00 |
| Lcn2 | NM_130741 | 2.29E+03 | 1.04E-02 | 1.44E-01 | 2.67E+00 | 2.67E+00 | 3.66E+02 | 1.37E+02 |
| Timp3 | NM_012886 | 3.79E+02 | 8.09E-03 | 1.33E-01 | 2.69E+00 | 2.69E+00 | 6.07E+01 | 2.26E+01 |
| Pcdhgc3 | NM_053943 | 7.22E+02 | 1.39E-03 | 8.31E-02 | 2.70E+00 | 2.70E+00 | 1.16E+02 | 4.30E+01 |
| Sdc1 | NM_013026 | 1.10E+03 | 4.51E-04 | 6.47E-02 | 2.72E+00 | 2.72E+00 | 1.77E+02 | 6.51E+01 |
| Slc35d1 | NM_001106668 | 4.11E+01 | 1.92E-02 | 1.85E-01 | 2.74E+00 | 2.74E+00 | 6.63E+00 | 2.42E+00 |
| Cdh5 | NM_001107407 | 2.21E+02 | 9.65E-03 | 1.42E-01 | 2.77E+00 | 2.77E+00 | 3.59E+01 | 1.30E+01 |
| Osmr | NM_001005384 | 7.91E+01 | 2.28E-03 | 9.95E-02 | 2.83E+00 | 2.83E+00 | 1.29E+01 | 4.57E+00 |
| Adam10 | NM_019254 | 2.81E+02 | 1.84E-03 | 9.56E-02 | 2.90E+00 | 2.90E+00 | 4.62E+01 | 1.60E+01 |
| Scd2 | NM_031841 | 1.02E+03 | 6.01E-03 | 1.26E-01 | 2.93E+00 | 2.93E+00 | 1.69E+02 | 5.77E+01 |
| Tnfrsf11b | NM_012870 | 6.43E+01 | 4.47E-03 | 1.19E-01 | 2.94E+00 | 2.94E+00 | 1.07E+01 | 3.62E+00 |
| Cpd | NM_012836 | 9.26E+01 | 1.03E-02 | 1.44E-01 | 2.94E+00 | 2.94E+00 | 1.53E+01 | 5.21E+00 |
| Adamts1 | NM_024400 | 4.94E+02 | 4.69E-03 | 1.21E-01 | 2.96E+00 | 2.96E+00 | 8.19E+01 | 2.77E+01 |
| Epcam | NM_138541 | 1.20E+03 | 7.34E-03 | 1.32E-01 | 2.97E+00 | 2.97E+00 | 2.00E+02 | 6.73E+01 |
| Fbn1 | NM_031825 | 3.22E+02 | 7.12E-04 | 6.85E-02 | 2.97E+00 | 2.97E+00 | 5.34E+01 | 1.80E+01 |
| Cd93 | NM_053383 | 2.29E+02 | 1.61E-02 | 1.72E-01 | 3.00E+00 | 3.00E+00 | 3.81E+01 | 1.27E+01 |
| App | NM_019288 | 1.16E+03 | 5.71E-04 | 6.47E-02 | 3.06E+00 | 3.06E+00 | 1.95E+02 | 6.36E+01 |
| Csf2rb | NM_133555 | 1.34E+02 | 9.71E-03 | 1.43E-01 | 3.07E+00 | 3.07E+00 | 2.24E+01 | 7.32E+00 |
| Gjb2 | NM_001004099 | 2.59E+02 | 2.20E-02 | 1.93E-01 | 3.10E+00 | 3.10E+00 | 4.37E+01 | 1.41E+01 |
| Cldn3 | NM_031700 | 8.25E+02 | 5.36E-03 | 1.24E-01 | 3.19E+00 | 3.19E+00 | 1.40E+02 | 4.40E+01 |
| Lbr | NM_134453 | 4.45E+01 | 4.32E-04 | 6.47E-02 | 3.31E+00 | 3.31E+00 | 7.65E+00 | 2.32E+00 |
| Nt5e | NM_021576 | 2.24E+02 | 5.48E-05 | 6.47E-02 | 3.41E+00 | 3.41E+00 | 3.89E+01 | 1.14E+01 |
| Fzd7 | NM_001271185 | 1.14E+02 | 1.47E-04 | 6.47E-02 | 3.70E+00 | 3.70E+00 | 2.03E+01 | 5.50E+00 |

**Table S4: Genes identified using more stringent conditions (HFD vs. SD)**

| **Symbol** | **Entrez Gene Name** | **Expr p-value** | **Expr False Discovery Rate (q-value)** | **Expr Fold Change** |
| --- | --- | --- | --- | --- |
| Abcg3l2 | ATP-binding cassette, subfamily G (WHITE), member 3-like 2 | 0.00000112 | 0.00963 | -3.831 |
| LOC257642 | rRNA promoter binding protein | 0.000419 | 0.0647 | -3.7 |
| Inafm1 | InaF motif containing 1 | 0.000101 | 0.0647 | -3.542 |
| Akap8l | A-kinase anchoring protein 8 like | 0.000223 | 0.0647 | -2.813 |
| Cdk10 | cyclin dependent kinase 10 | 0.000262 | 0.0647 | -2.76 |
| Acsm3 | acyl-CoA synthetase medium chain family member 3 | 0.000375 | 0.0647 | -2.698 |
| Il3ra | interleukin 3 receptor subunit alpha | 0.0000899 | 0.0647 | -2.409 |
| Nxf1 | nuclear RNA export factor 1 | 0.000266 | 0.0647 | -2.391 |
| Atg16l2 | autophagy related 16 like 2 | 0.00022 | 0.0647 | -2.244 |
| Uspl1 | ubiquitin specific peptidase like 1 | 0.000203 | 0.0647 | -2.23 |
| Tle2 | transducin like enhancer of split 2 | 0.0000336 | 0.0647 | -2.215 |
| Vill | villin like | 0.000433 | 0.0647 | -2.209 |
| Akap17a | A-kinase anchoring protein 17A | 0.000385 | 0.0647 | -2.193 |
| Hdac10 | histone deacetylase 10 | 0.000706 | 0.0685 | -2.185 |
| Safb2 | scaffold attachment factor B2 | 0.0000528 | 0.0647 | -2.162 |
| RGD1562114 | chromosome 19 open reading frame 24 | 0.000295 | 0.0647 | -2.134 |
| Miip | migration and invasion inhibitory protein | 0.000516 | 0.0647 | -2.131 |
| Cep95 | centrosomal protein 95 | 0.000166 | 0.0647 | -2.098 |
| Elmo3 | engulfment and cell motility 3 | 0.000661 | 0.0685 | -2.098 |
| Haus7 | HAUS augmin like complex subunit 7 | 0.000365 | 0.0647 | -2.093 |
| Abcb6 | ATP binding cassette subfamily B member 6 (Langereis blood group) | 0.000411 | 0.0647 | -2.042 |
| Clasrp | CLK4-associating serine/arginine rich protein | 0.000322 | 0.0647 | -1.988 |
| Spata7 | spermatogenesis associated 7 | 0.000393 | 0.0647 | -1.982 |
| Nosip | nitric oxide synthase interacting protein | 0.000277 | 0.0647 | -1.978 |
| Tra2a | transformer 2 alpha homolog | 0.000259 | 0.0647 | -1.967 |
| Mmgt2 | membrane magnesium transporter 2 | 0.000605 | 0.0662 | -1.96 |
| Trit1 | tRNA isopentenyltransferase 1 | 0.000244 | 0.0647 | -1.951 |
| Tpcn2 | two pore segment channel 2 | 0.000157 | 0.0647 | -1.916 |
| Mtif2 | mitochondrial translational initiation factor 2 | 0.000575 | 0.0647 | -1.905 |
| Deaf1 | DEAF1, transcription factor | 0.000306 | 0.0647 | -1.894 |
| Rpap3 | RNA polymerase II associated protein 3 | 0.000203 | 0.0647 | -1.859 |
| Clk2 | CDC like kinase 2 | 0.000574 | 0.0647 | -1.85 |
| Zfp775 | zinc finger protein 775 | 0.000548 | 0.0647 | -1.846 |
| Wdr83 | WD repeat domain 83 | 0.000258 | 0.0647 | -1.841 |
| Tbce | tubulin folding cofactor E | 0.000218 | 0.0647 | -1.834 |
| Rps6kb2 | ribosomal protein S6 kinase B2 | 0.000454 | 0.0647 | -1.794 |
| Zc3h7a | zinc finger CCCH-type containing 7A | 0.000287 | 0.0647 | -1.788 |
| Fuz | fuzzy planar cell polarity protein | 0.000238 | 0.0647 | -1.776 |
| Coq10a | coenzyme Q10A | 0.000702 | 0.0685 | -1.774 |
| Armc5 | armadillo repeat containing 5 | 0.000578 | 0.0647 | -1.753 |
| Exoc3l1 | exocyst complex component 3 like 1 | 0.000238 | 0.0647 | -1.747 |
| Tada2a | transcriptional adaptor 2A | 0.000562 | 0.0647 | -1.718 |
| Cenpj | centromere protein J | 0.000258 | 0.0647 | -1.698 |
| Mpnd | MPN domain containing | 0.000492 | 0.0647 | -1.606 |
| Coq5 | coenzyme Q5, methyltransferase | 0.000337 | 0.0647 | -1.587 |
| Tpp1 | tripeptidyl peptidase 1 | 0.000681 | 0.0685 | 1.562 |
| Gpr155 | G protein-coupled receptor 155 | 0.000703 | 0.0685 | 1.578 |
| Nomo1 | NODAL modulator 1 | 0.000618 | 0.0662 | 1.66 |
| Ifngr2 | interferon gamma receptor 2 | 0.000352 | 0.0647 | 1.667 |
| Txndc5 | thioredoxin domain containing 5 | 0.000528 | 0.0647 | 1.708 |
| Slc35e1 | solute carrier family 35 member E1 | 0.000616 | 0.0662 | 1.748 |
| Abcd1 | ATP binding cassette subfamily D member 1 | 0.000526 | 0.0647 | 1.75 |
| Minpp1 | multiple inositol-polyphosphate phosphatase 1 | 0.000153 | 0.0647 | 1.804 |
| Manba | mannosidase beta | 0.000164 | 0.0647 | 1.824 |
| Atp1a1 | ATPase Na+/K+ transporting subunit alpha 1 | 0.000288 | 0.0647 | 1.877 |
| Mfsd1 | major facilitator superfamily domain containing 1 | 0.000696 | 0.0685 | 1.9 |
| M6pr | mannose-6-phosphate receptor, cation dependent | 0.000517 | 0.0647 | 1.909 |
| Tapbpl | TAP binding protein like | 0.000581 | 0.0647 | 1.909 |
| Grn | granulin precursor | 0.000477 | 0.0647 | 1.917 |
| Tmed10 | transmembrane p24 trafficking protein 10 | 0.000409 | 0.0647 | 1.959 |
| Scarb2 | scavenger receptor class B member 2 | 0.000435 | 0.0647 | 1.995 |
| Tapbp | TAP binding protein | 0.000311 | 0.0647 | 2.017 |
| Arsb | arylsulfatase B | 0.000519 | 0.0647 | 2.065 |
| Acvr1 | activin A receptor type 1 | 0.000627 | 0.0664 | 2.077 |
| Ddost | dolichyl-diphosphooligosaccharide--protein glycosyltransferase non-catalytic subunit | 0.000419 | 0.0647 | 2.096 |
| Lrrc8d | leucine rich repeat containing 8 VRAC subunit D | 0.0000176 | 0.0502 | 2.11 |
| Suco | SUN domain containing ossification factor | 0.000474 | 0.0647 | 2.125 |
| Tspan14 | tetraspanin 14 | 0.0004 | 0.0647 | 2.132 |
| Lrrc8a | leucine rich repeat containing 8 VRAC subunit A | 0.000564 | 0.0647 | 2.19 |
| Slc43a2 | solute carrier family 43 member 2 | 0.000222 | 0.0647 | 2.225 |
| Ctsc | cathepsin C | 0.000376 | 0.0647 | 2.225 |
| Sft2d2 | SFT2 domain containing 2 | 0.000387 | 0.0647 | 2.242 |
| Tmtc4 | transmembrane and tetratricopeptide repeat containing 4 | 0.000706 | 0.0685 | 2.242 |
| Reep3 | receptor accessory protein 3 | 0.000308 | 0.0647 | 2.309 |
| Erlin2 | ER lipid raft associated 2 | 0.0000141 | 0.0502 | 2.42 |
| Tmx2 | thioredoxin related transmembrane protein 2 | 0.0000845 | 0.0647 | 2.49 |
| Golim4 | golgi integral membrane protein 4 | 0.000099 | 0.0647 | 2.5 |
| Ptprg | protein tyrosine phosphatase, receptor type G | 0.000496 | 0.0647 | 2.519 |
| Cd14 | CD14 molecule | 0.000449 | 0.0647 | 2.521 |
| Hsd11b1 | hydroxysteroid 11-beta dehydrogenase 1 | 0.000316 | 0.0647 | 2.544 |
| Ikbip | IKBKB interacting protein | 0.000461 | 0.0647 | 2.653 |
| Sdc1 | syndecan 1 | 0.000451 | 0.0647 | 2.723 |
| Fbn1 | fibrillin 1 | 0.000712 | 0.0685 | 2.972 |
| App | amyloid beta precursor protein | 0.000571 | 0.0647 | 3.06 |
| Lbr | lamin B receptor | 0.000432 | 0.0647 | 3.306 |
| Nt5e | 5'-nucleotidase ecto | 0.0000548 | 0.0647 | 3.413 |
| Fzd7 | frizzled class receptor 7 | 0.000147 | 0.0647 | 3.697 |

**Table S5:** Description of some of the molecules in Table 1

| **Ingenuity Canonical Pathway** | **Molecule** | **Description** |
| --- | --- | --- |
| Molecular Mechanisms of Cancer | FZD1 (P<0.005) | Frizzled transmembrane domain protein. Involved in Wnt/β-Catenin signaling pathway. |
|  | CDK10 (P<0.005) | Serine/Threonine kinase. Phosphorylates ETS transcription factor involved in protein degradation and proliferation. |
|  | APC | Adenomatous Polyposis Colon gene tumor suppressor protein involved particularly in colon cancer. Antagonist to Wnt pathway. |
| Epithelial Adherens Junction Signaling | ACVR1 (P<0.005) | Activin A Receptor Type 1. Member of TGFβ family involved in membrane signaling. Specifically involved in complex with bone morphogenesis. This complex interacts with multiple STAT proteins. |
|  | SNAI2 | Snail zinc finger transcription factor. Transcriptional repressor of E-cadherin transcription in breast cancer. Stimulates mesenchymal phenotype. |
| Th1 and Th2 Activation Pathway | IFNGR2 (P<0.005) | Interferon Gamma Receptor 2. Non-ligand β chain of gamma interferon binding receptor. Defects in this receptor cause susceptibility to mycobacterial infections in man. |
|  | NFKB | Proinflammatory protein involved in transcription of DNA and cytokine production. |
| Antigen Presentation Pathway | TAPBP (P<0.005) | TAP-associated glycoprotein. Transmembrane glycoprotein that mediates interaction between newly assembled major histocompatibility complex (MHC) molecules and the transporter associated with antigen processing (TAP) required for transport of antigenic peptides across the endoplasmic reticulum. |
| LXR/RXR Activation | CD14 (P<0.005) | Surface antigen preferentially expressed on monocytes/macrophages. Part of innate immune system that mediates response to bacterial LPS. |
|  | Srebf1 (P<0.05) | Sterol regulatory element binding transcription factor 1. Transcription factor that binds to the sterol regulatory element 1 found flanking low density lipoprotein receptor gene and other genes involved in sterol metabolism. |
|  | APOE | Apolipoprotein E binds with fats (lipids) in body to form lipoproteins implicated in Alzheimer’s and cardiovascular disease. |
